# Platinum and microspherule peaks as chronostratigraphic markers for onset of the Younger Dryas at Wakulla Springs, Florida

**DOI:** 10.1038/s41598-023-50074-8

**Published:** 2023-12-20

**Authors:** Christopher R. Moore, Mark J. Brooks, James S. Dunbar, C. Andrew Hemmings, Kurt A. Langworthy, Allen West, Malcolm A. LeCompte, Victor Adedeji, James P. Kennett, James K. Feathers

**Affiliations:** 1 South Carolina Institute of Archaeology and Anthropology, University of South Carolina, P.O. Box 400, New Ellenton, SC 29809 USA; 2 Aucilla Research Institute Inc., 555 North Jefferson Street, Monticello, FL 32344 USA; 3 CAMCOR, University of Oregon, 1443 E 13Th Ave, Eugene, OR 97403 USA; 4 Comet Research Group, Prescott, AZ USA; 5 Center of Excellence in Remote Sensing Education and Research, Elizabeth City State University, Elizabeth City, NC 27909 USA; 6 Department of Natural Sciences, Elizabeth City State University, Elizabeth City, NC 27909 USA; 7 Department of Earth Science and Marine Science Institute, University of California, Santa Barbara, CA 93106 USA; 8 Luminescence Dating Laboratory, University of Washington, 125 Raitt Hall, Seattle, WA 98195-3412 USA

**Keywords:** Geology, Sedimentology, Anthropology, Archaeology, Solid Earth sciences

## Abstract

Anomalous peak abundances of platinum and Fe-rich microspherules with high-temperature minerals have previously been demonstrated to be a chronostratigraphic marker for the lower Younger Dryas Boundary (YDB) dating to 12.8 ka. This study used Bayesian analyses to test this hypothesis in multiple sequences (units) of sandy, weakly stratified sediments at Wakulla Springs, Florida. Our investigations included platinum geochemistry, granulometry, optically stimulated luminescence (OSL) dating, and culturally dated lithics. In addition, sediments were analyzed using scanning electron microscopy and energy dispersive x-ray spectroscopy to investigate dendritic, iron-rich microspherules previously identified elsewhere in peak abundances at the onset of the Younger Dryas (YD) cool climatic episode. Our work has revealed this abundance peak in platinum and dendritic spherules in five sediment sequences at Wakulla Springs. A YDB age of ~ 12.8 ka for the platinum and spherule chronostratigraphic datum in these Wakulla Springs sequences is consistent with the archaeological data and OSL dating. This study confirms the utility of this YDB datum layer for intersequence correlation and for assessing relative ages of Paleoamerican artifacts, including those of likely Clovis, pre-Clovis, and post-Clovis age and their possible responses to environmental changes known to have occurred during the Younger Dryas cool climatic episode.

## Introduction

Early work by Jones^1^; Jones and Tesar^2^ at Wakulla Springs demonstrated that deeply buried archaeological deposits are present adjacent to the spring and have great potential to assist with the understanding of Florida’s early Paleoamerican occupation. In 2017 and 2018, a geoarchaeological study of Wakulla Springs sites (8WA329 and 8WA1221) was undertaken to describe the site sediments, interpret formation processes and archaeostratigraphy, and establish a geochronology of multiple archaeological deposits containing Paleoamerican artifact assemblages. Our geoarchaeological investigations included the excavation of multiple sediment columns specifically to study the granulometry and platinum geochemistry of select excavation unit profiles. The sandy sediments at Wakulla Springs contain insufficient organic material required for radiocarbon dating, and hence, samples were collected for optically stimulated luminescence (OSL) dating. Also, sediments from two unit profiles were examined for magnetic iron-rich microspherules.

In investigations of abrupt climate changes during the last Glacial episode, the Allerød/Younger Dryas boundary (the onset of YD or GS-1) was dated in the Greenland Ice Core record to 12,896 ± 4 years (maximum counting error of 138 years) (Rasmussen et al*.*^3^). Prior to this, Petaev et al*.*^4^ identified a ca.12,800-year-old platinum anomaly peak at the YD onset in the Greenland ice sheet. They proposed that the peak abundances were produced by a cosmic impact event. Later in 2017 and again in 2019, this same Pt anomaly was observed in multiple sequences across North America by Moore et al*.*^5,6^ and at a site in Syria^7–10^. We here investigate whether a platinum anomaly is present in the Wakulla Springs site and whether it can be employed as a chronostratigraphic marker for identifying the lower Younger Dryas Boundary (YDB) at ca. ~ 12,800 cal BP. Furthermore, we investigated whether the platinum anomaly can assist in determining depths at which early Paleoamerican artifacts may be located. We also analyzed sediment samples for the presence of iron-rich microspherules that have also been reported in association with sediments dating to the YD onset, both across North America and globally, and are also proposed to provide evidence of a cosmic impact or airburst event^5–21^. This investigation focused on testing the utility of peak platinum and microspherule anomalies as chronostratigraphic markers rather than their potential origins. See Supplementary Table 1*, “List of Important YDB Papers (pro and con).”* Below, we present results based on the analysis of multiple data sets we generated from 7 separate sequences collected during several years of fieldwork at Wakulla Springs sites 8WA329 and 8WA1221.

## Study site

**Figure 1:**
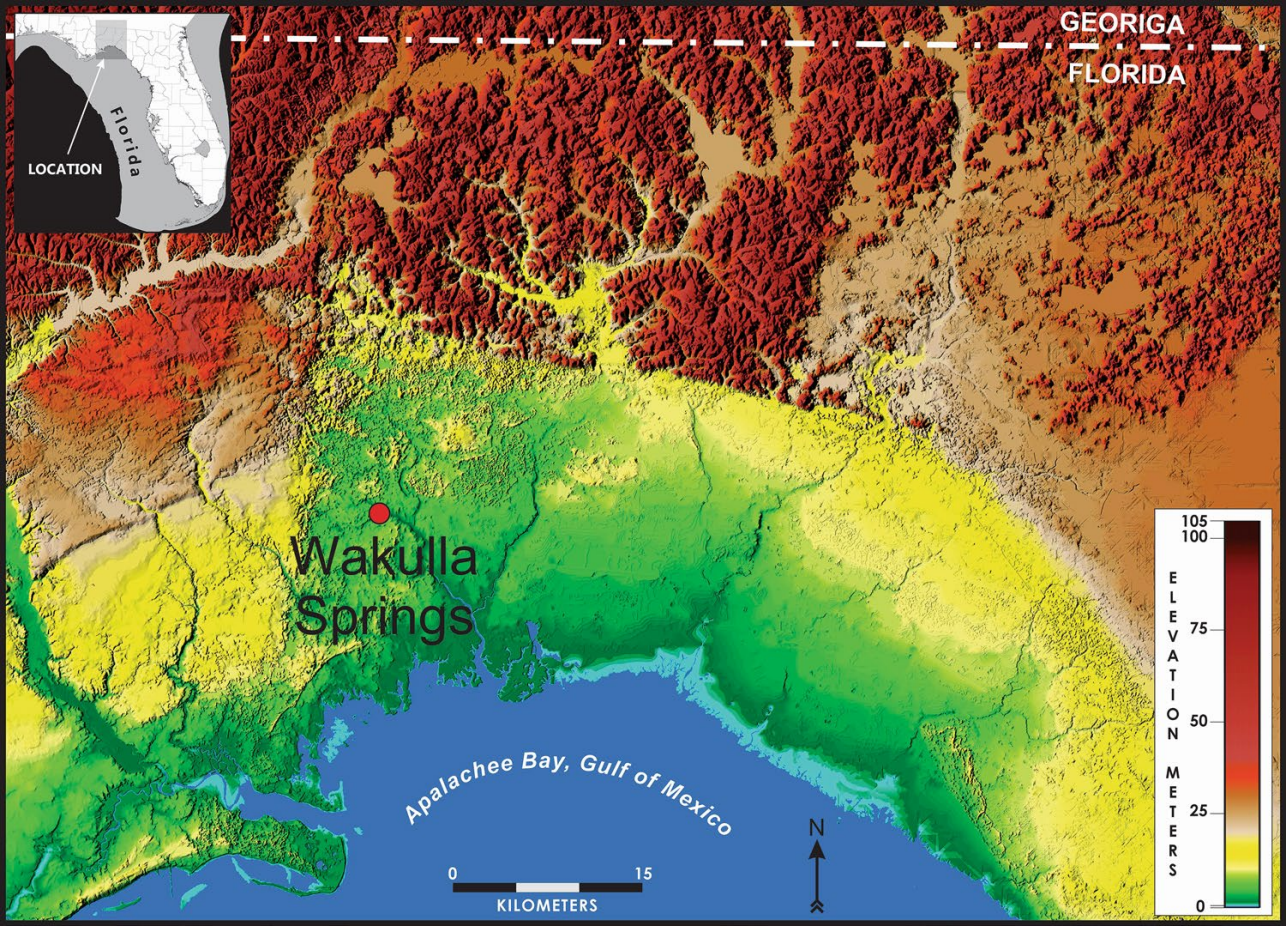
Wakulla Springs Site location, northern Florida. LiDAR map was produced in Global Mapper (v.19.1), https://www.bluemarblegeo.com/global-mapper/.

Wakulla Springs State Park lies approximately 15 miles south of Tallahassee in Wakulla County, Florida (Fig. 1). The Wakulla River is derived from the springhead which flows at a rate of approximately 154 cubic feet per second (US Geological Survey; USGS website). Springs are common throughout the karst regions of Florida and numerous springs occur in this area. It is uncertain if the spring was completely exposed during the early human Paleoamerican occupation of the springs; however, abundant evidence of extinct vertebrate remains near the spring, including the American mastodon (*Mammut americanum*) and southern mammoth (*Mammuthus columbi*), support the “watering hole” model originally proposed by Wilfred Neill^22^ and expanded by Dunbar^23,24^.

Investigations at the nearby Page-Ladson site in the Aucilla River indicate that inland water tables fluctuated with late Pleistocene climate oscillations. Substantially cooler and drier conditions during Glacial and Heinrich intervals resulted in reduced freshwater availability for animals and early Paleoamericans (Dunbar^25,26^; Thulman^27^; Webb^28^). During Modern-analog climate conditions of the last glacial recession, water tables rose to near-modern levels and rivers began flowing again.

Ponds, lakes, and streams would have been less abundant across the landscape during the drier last glacial episode compared with the Holocene, when inland water tables were higher and had reached a relatively steady state. The resulting decrease in available surface waters during drier climatic conditions of the latest Pleistocene made Wakulla Springs an ideal environment for exploitation by Florida’s early inhabitants. This would have been especially the case during drought conditions marking the transition from Heinrich I to the Bølling/Allerød Interstadial in Florida (Dunbar^23^).

The Wakulla Springs Archaeological and Historical District (8WA315) encompasses 61 archaeological sites distributed over nearly 3000 acres. Almost every cultural or temporal group represented in the northwest region of Florida left evidence of at least an ephemeral occupation of Wakulla Springs (Milanich^29^). Excavations by the Aucilla Research Institute, Inc. at Wakulla Springs in 2017 and 2018 included multiple blocks consisting of 2-m × 2-m grid squares. These grid areas were placed in areas determined in 2015 to exhibit artifact concentrations west and north of the lodge. An important objective of the work at Wakulla Springs is to better define chronologically the early Paleoamerican Clovis, Suwannee, and Simpson technocomplexes widespread in Florida but which lack precise chronological control (Supplementary Fig. 23). The work at Wakulla Springs also figures prominently in the debate over the pre-Clovis occupation of Florida (e.g., Page Ladson Site on the Aucilla River) (Halligan et al*.*^30^; Dunbar^23,24^).

In our research, excavation units were divided into quadrants, designated southwest, northwest, northeast, and southeast and each was excavated and screened separately. The upper layer of organic material and gray humus, called the “duff” layer, was removed as one 30-cm-thick level and screened through 1/4′′ and 1/16″ mesh to recover all archaeological material. Each unit was excavated by quadrant in arbitrary 15-cm-thick levels until reaching sediment below the disturbed duff levels, at which point 10-cm-thick and eventually 5-cm-thick levels were excavated. A geoarchaeological sampling of completed unit profiles included sediment columns that were sampled continuously at 2.5-cm intervals. Geoarchaeological analyses included OSL dating, sediment geochemistry, granulometry, and analysis of processed sediment samples for iron-rich microspherules.

## Ages of sequences

### Luminescence dating (OSL)

**Figure 2:**
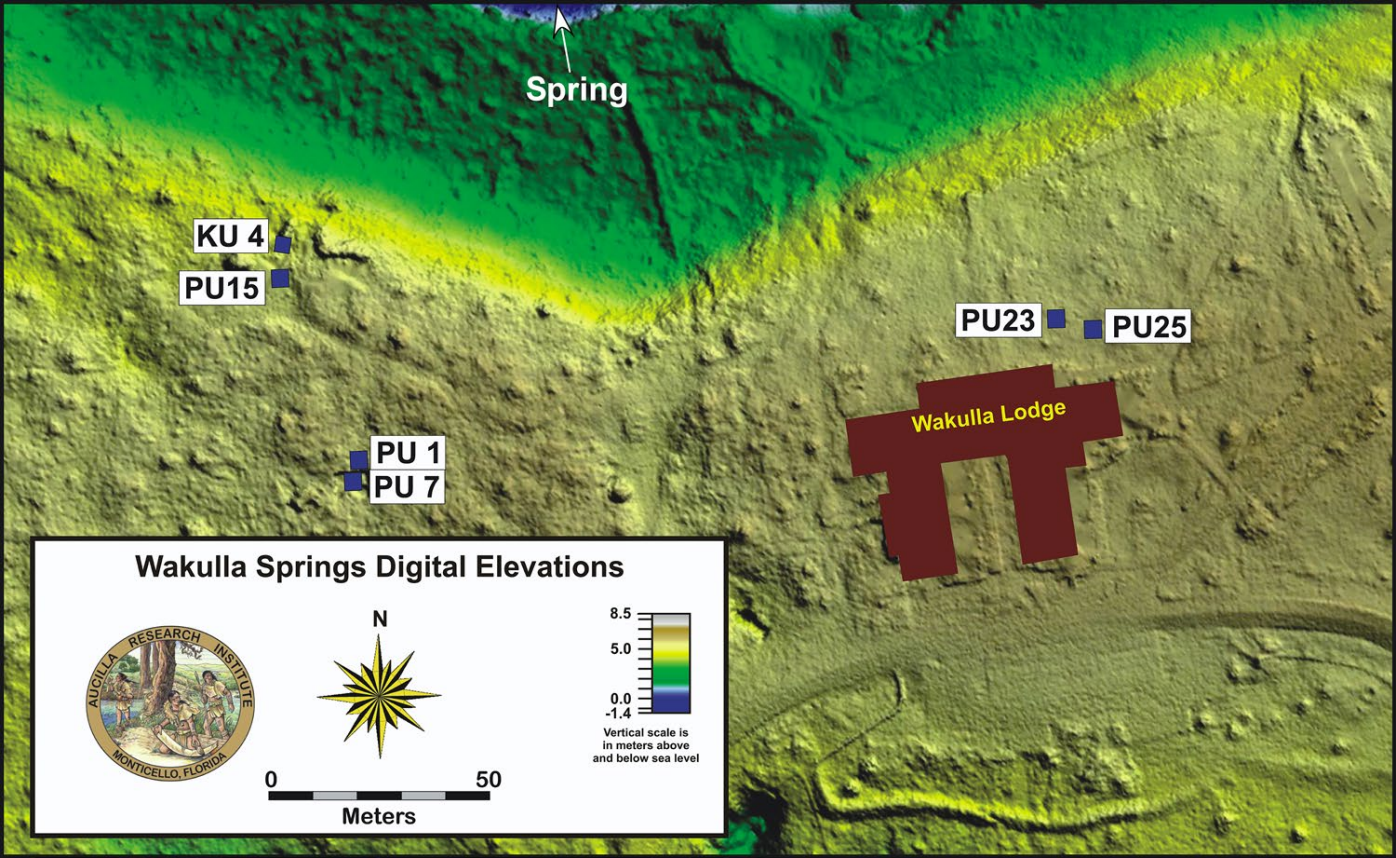
Archaeological Test Units sampled for this study. WH1 ~ 1 km to the east. LiDAR map was produced in Global Mapper (v.19.1), https://www.bluemarblegeo.com/global-mapper/.

Luminescence dating was conducted on 17 samples from 6 Test Units at 8WA329 and 8WA1221 (Fig. 2) at the Luminescence Dating Laboratory, University of Washington. Luminescence dates sediments to their last exposure to light. It is based on the build-up of absorbed energy in minerals, such as quartz and feldspar, from natural radioactivity. This energy is released, in part, by light called luminescence when exposed to the sun, a process called bleaching. Upon burial, the absorbed energy accumulates again and can be measured in the laboratory by exposure to artificial light. The measured luminescence intensity is translated into what is called an equivalent dose by measuring its sensitivity to laboratory doses. Dividing the equivalent dose by the natural dose rate yields an age. The equivalent dose can be measured on individual grains of quartz or feldspar producing a distribution of equivalent dose values. For well-bleached, unmixed samples, this distribution should be rather narrow, especially if possible differences in dose rate at the scale of single grains are taken into account as was done here.

The results on the Wakulla Springs samples, using fine quartz sand grains, show a broad distribution. Since eolian sediments are usually well bleached, this suggests significant mixing of sand grains, making estimation of the true burial age difficult. The structure of the distributions was evaluated using a finite mixture model (Galbraith and Roberts^31^), which uses maximum likelihood to divide the distribution into components, each of which is statistically consistent with a single value. The model is most effective where the dose rate varies little with depth, as is the case for these sequences. The model requires the input of an over-dispersion value considered typical of a single-age distribution. Over-dispersion is a measure of spread and can be thought of as the percentage of grains whose equivalent dose values cannot be accounted for by measurement error. Over-dispersion values of 10 to 15% were used for these samples, slightly more than that obtained in dose recovery experiments. Dose recovery involves measuring the equivalent dose on single grains that have been given the same dose. The two most abundant components are illustrated in the radial graphs (Supplementary Figs. 24–40) in the supplementary material “*OSL Radial Graphs.*” It is important to assay whether the components represent different ages of the grains rather than variable dose rates at the scale of single grains. Variable dose rates to quartz grains are often caused by differential distribution of beta emitters, notably ^40^K in potassium feldspars. The potential of this was modeled for these samples following Chauhan et al*.*^32^, similar to that described in Feathers et al*.*^33^. By this model, it was found that the structure of the distributions was too broad to be explained by differing dose rates. Thus, the best explanation is that the resulting differences in values do indeed represent different ages, best accounted for by post-depositional mixing. The different components do not necessarily represent discrete groups, although the radial graphs for many samples suggest modality. The components could just mean groups isolated from a continuum by the restraints of the model.

All samples exhibit multiple age components, which is common for such sandy sediment sequences (Moore et al.^34^). This mixing almost certainly resulted from bioturbation and physical processes, as well as anthropogenic disturbance by prehistoric and even modern occupants. The most likely age estimates based on OSL dating, collected from PU1 (n = 2), KU4 (n = 3), PU15 (n = 2), PU7 (n = 3), PU23 (n = 4), and WH1 (n = 3) are given in Table 1. Radial graphs for all samples are shown in *Supplementary Information “OSL Radial Graphs”*.

**Table 1:** Dosimetry Data and Basis for Age for OSL Dates Discussed in the Text.

UW lab#	Site	Unit	Depth (m)	Ppm U	Ppm Th	% K	Dose rate (Gy/ka)	N (accepted)	D_e_(Gy) CAM	Od (%)	D_e_(Gy)	D_e_(Gy)	Age (ka)	Basis for age
											FMM 1^st^	FMM 2^nd^		
											(%)	(%)		
UW3689	8WA329	PU1	0.9	3.37 ± 0.21	5.79 ± 0.92	0.08 ± 0.01	0.97 ± 0.07	489 (129)	4.30 ± 0.33	74.7 ± 6.0	2.73 ± 0.19	4.05 ± 0.25	10.3 ± 1.2	FMM
											−34	−33		3rd
UW3690	8WA329	PU1	1.3	2.43 ± 0.12	6.41 ± 0.67	0.05 ± 0.01	0.87 ± 0.06	582 (151)	9.85 ± 0.69	78.1 ± 5.3	7.72 ± 0.39	18.9 ± 1.22	20.6 ± 2.74	FMM
											−34	−22		2nd
UW3691	8WA329	KU4	0.9	1.33 ± 0.09	1.15 ± 0.38	0.06 ± 0.01	0.57 ± 0.04	584 (162)	6.90 ± 0.46	75.3 ± 5.2	6.82 ± 0.29	3.41 ± 0.14	12.0 ± 1.29	CAM
											−40	−39		
UW3692	8WA329	KU4	0.96	0.80 ± 0.08	2.58 ± 0.62	0.09 ± 0.01	0.67 ± 0.04	981 (151)	7.47 ± 0.46	64.0 ± 5.0	6.44 ± 0.42 (40)	4.02 ± 0.33	9.66 ± 1.01	FMM
												−32		1st
UW3693	8WA329	KU4	1.11	1.83 ± 0.13	4.00 ± 0.73	0.02 ± 0.01	0.89 ± 0.06	576 (127)	11.2 ± 0.76	63.8 ± 5.4	18.2 ± 1.01	5.07 ± 0.21	20.5 ± 1.88	FMM
											−37	−32		1st
UW3789	8WA329	PU7	0.9	1.46 ± 0.10	2.14 ± 0.57	0.07 ± 0.01	0.68 ± 0.05	677 (105)	8.48 ± 0.67	69.9 ± 6.2	7.73 ± 0.73	4.51 ± 0.44	11.3 ± 1.43	FMM
											−38	−35		1st
UW3790	8WA329	PU7	1.07	1.54 ± 0.12	3.63 ± 0.74	0.06 ± 0.01	0.77 ± 0.06	582 (117)	9.40 ± 0.66	64.7 ± 5.6	9.69 ± 0.49	5.75 ± 0.41	12.5 ± 1.2	FMM
											−44	−38		1st
UW3788	8WA329	PU7	1.3	1.19 ± 0.10	2.67 ± 0.62	0.07 ± 0.01	0.56 ± 0.05	487 (103)	11.2 ± 0.66	47.6 ± 5.0	12.5 ± 0.58	6.01 ± 0.34	22.4 ± 2.37	FMM
											−56	−29		1st
UW3787	8WA329	PU15	1.025	4.58 ± 0.32	14.04 ± 1.65	0.23 ± 0.02	1.15 ± 0.07	777(94)	22.6 ± 2.11	82.1 ± 7.5	34.2 ± 1.17 −68	4.11 ± 0.38 −13	12.8 ± 1.18	FMM 2nd
UW3786	8WA329	PU15	1.2	2.21 ± 0.17	7.32 ± 1.09	0.21 ± 0.01	1.21 ± 0.08	575 (88)	14.8 ± 1.45	82.1 ± 7.5	39.5 ± 2.38	6.86 ± 0.47	12.9 ± 1.35	FMM
											−28	−28		2nd
UW3813	8WA329	PU23	0.9	1.72 ± 0.13	5.52 ± 0.85	0.04 ± 0.01	0.73 ± 0.06	393 (70)	6.65 ± 0.63	65.7 ± 7.8	5.69 ± 0.35	3.28 ± 0.33	9.09 ± 1.19	CAM
											−52	−25		
UW3814	8WA329	PU23	1.03	1.29 ± 0.09	1.48 ± 0.48	0.08 ± 0.01	0.54 ± 0.05	389 (87)	6.84 ± 0.57	69.6 ± 6.4	6.19 ± 0.27 (38)	11.8 ± 0.64	12.6 ± 1.55	CAM
												−30		
UW3815	8WA329	PU23	1.2	1.90 ± 0.12	2.30 ± 0.50	0.03 ± 0.01	0.54 ± 0.05	494 (72)	13.8 ± 1.30	70.0 ± 7.4	17.1 ± 0.89	34.3 ± 2.66	25.7 ± 3.44	CAM
											−36	−25		
UW3816	8WA329	PU23	1.85	1.06 ± 0.08	1.54 ± 0.44	0.01 ± 0.01	0.47 ± 0.04	881 (113)	31.9 ± 1.77	43.0 ± 4.9	32.6 ± 1.94	58.5 ± 6.53	76.2 ± 9.31	CAM
											−74	−15		
UW3819	8WA1221	WH1	1	2.63 ± 0.18	6.09 ± 0.93	0.02 ± 0.03	0.81 ± 0.07	487 (70)	5.94 ± 0.56	68.8 ± 7.5	3.45 ± 0.18	9.54 ± 0.53	11.7 ± 1.26	FMM
											−52	−38		2nd
UW3820	8WA1221	WH1	1.3	0.76 ± 0.07	1.15 ± 0.41	0.02 ± 0.01	0.42 ± 0.04	780 (94)	10.3 ± 0.88	68.8 ± 7.1	11.4 ± 0.77	3.79 ± 0.28	27.3 ± 3.40	FMM
											−49	−28		1st
UW3821	8WA1221	WH1	0.35	1.83 ± 0.14	4.53 ± 0.83	0.03 ± 0.03	0.73 ± 0.06	1174 (82)	2.30 ± 0.21	64.4 ± 7.7	1.50 ± 0.11	3.63 ± 0.30	2.74 ± 0.37	CAM

U, Th and K contents are determined from alpha counting and flame photometry. Some adjustments to these values for calculating the dose rate are made for many samples so that the values agree with results from beta counting. The discrepancy is thought due to disequilibrium in the U decay chain. The dose rate is also adjusted for 10 ± 5% moisture content. N is the total number of grains measured. In parentheses are the number of grains that passed all acceptance criteria. Equivalent dose (De) is determined by both the central age model (CAM) and the finite mixture model (FMM) (Galbraith and Roberts 2012). OD is the over-dispersion, a measure of spread. De for the FMM is only given for the two most abundant components with the percentage of grains assigned to that component in parentheses. For UW3689, the age is based on the third most abundant component, which is 10.0 ± 0.84 Gy with 16% of the grains.

The different age components are used to evaluate the age of each sample. Other information, such as stratigraphy and artifacts, are used to select components most likely to represent the depositional age. We also have well-dated YDB microspherule and platinum peaks at more than 40 sites across 5 continents that, when present, provide useful chronostratigraphic anchors for selecting OSL age components (e.g., Wittke et al*.*^14^; Moore et al.^5,6^). The ages can thus be constrained by artifacts, stratigraphy, and the platinum and microspherule peaks. This evidence converges to produce a useful chronology.

**Figure 3:**
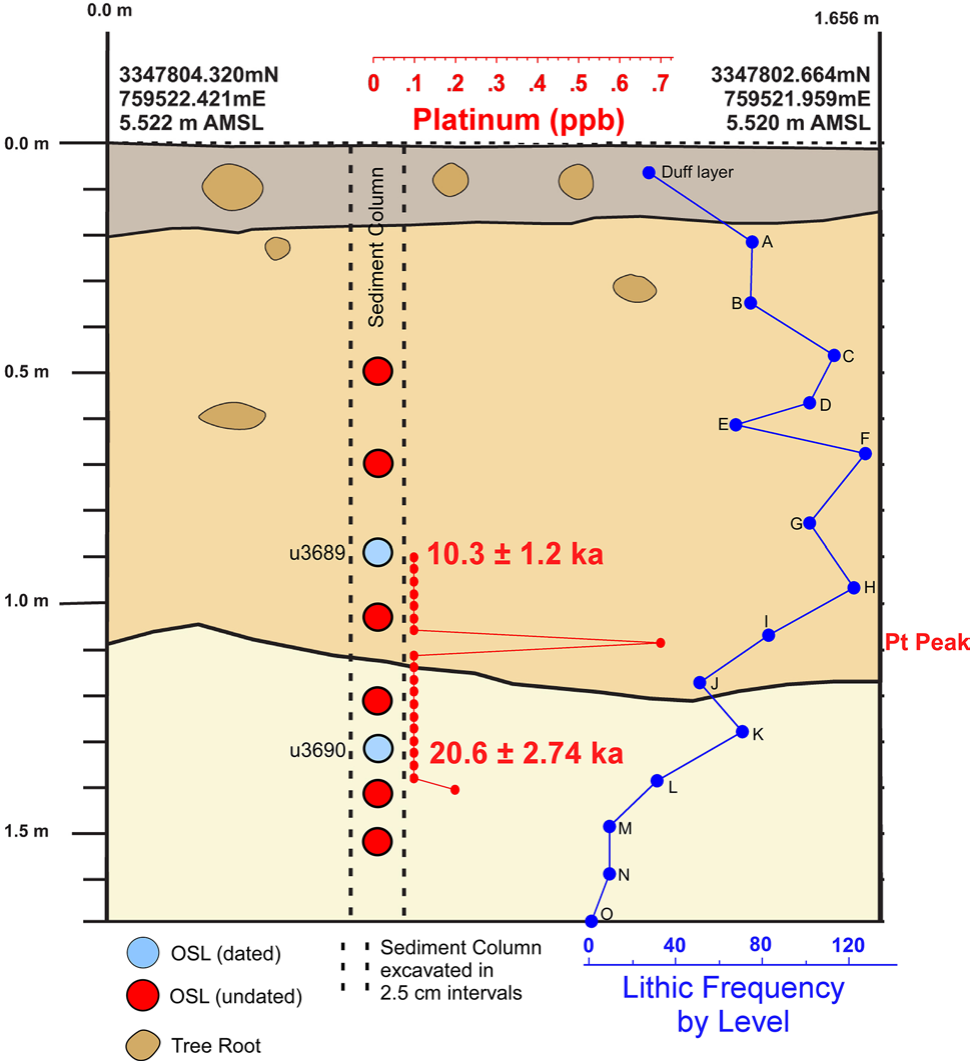
Paleo Unit 1 (PU1) profile and sampling levels at site 8WA329 showing platinum (Pt) abundance record in ppb (error =  ± 0.1 ppb) (Supplementary Table 2), OSL age estimates (Table 1), and generalized archaeostratigraphic data (**A**–**O**) representing multi-modal lithic artifact frequencies. Sediment zones as observed visually are indicated by color changes.

PU1 grain ages are mixed, suggesting a high degree of vertical mixing, with a large proportion of young grains even at depths of 90 and 130 cm below the surface (cmbs). Nevertheless, grain groupings in each of these samples provide ages that are consistent with sample depth and generalized archaeostratigraphy (Fig. 3 and Table 1). A grouping from 90 cmbs has an age of 10.3 ± 1.2 ka, consistent with the archaeological evidence. Additionally, one grain component at 130 cmbs has an age of 20.6 ± 2.74 ka.

**Figure 4:**
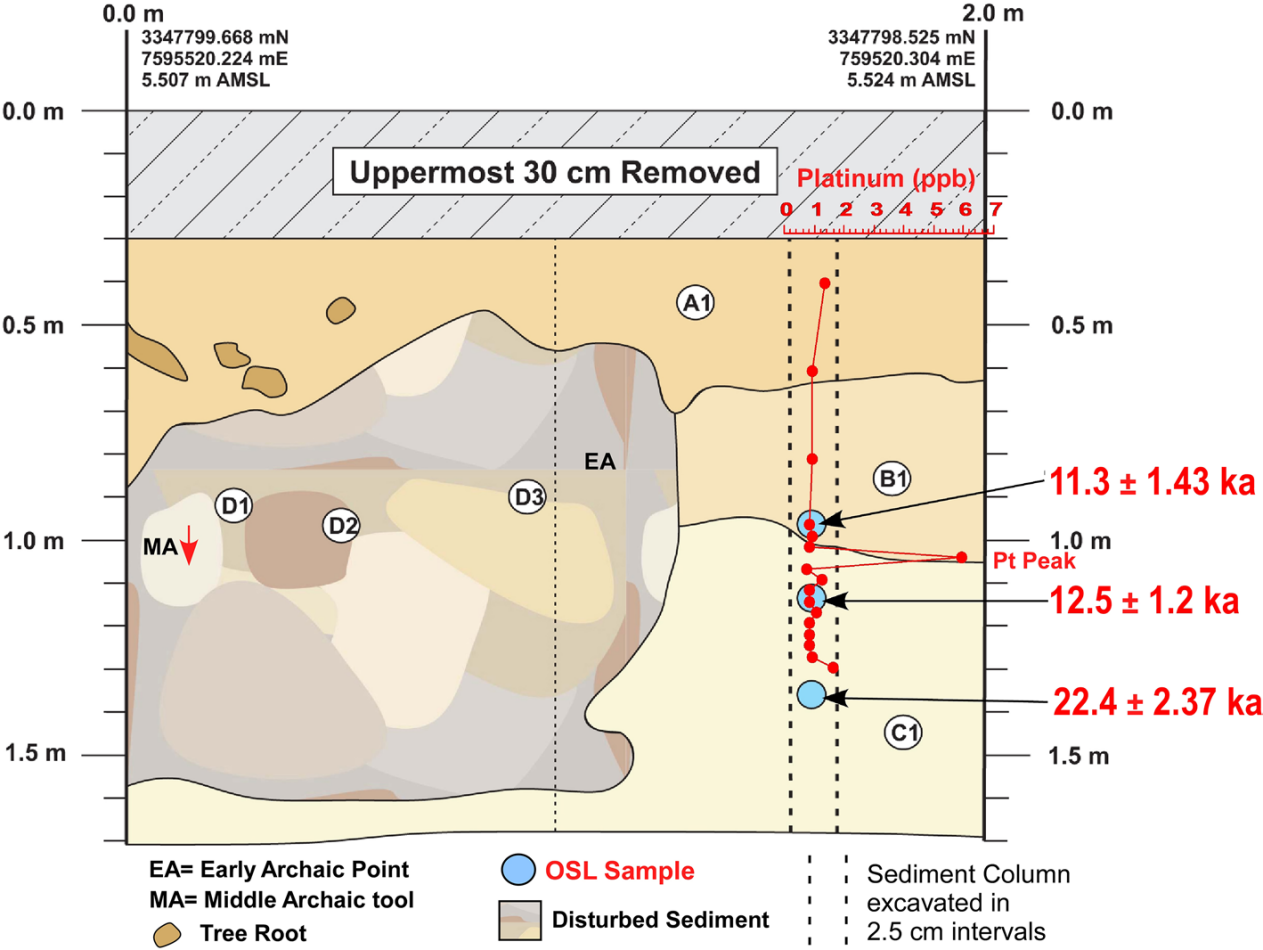
Paleo Unit 7 (PU7) profile at site 8WA329 showing platinum (Pt) abundance record in ppb (error =  ± 0.1 ppb) (Supplementary Table 3), OSL age estimates (Table 1), and position of temporally diagnostic artifacts. The red arrow indicates the direction of inferred vertical displacement of temporally diagnostic artifacts. Sediment zones as observed visually are indicated by color changes.

PU7 age estimates occur in regular stratigraphic order ranging from Early Archaic (early Holocene) to Last Glacial Maximum (LGM) in age (Fig. 4 and Table 1). The youngest sample at 93 cmbs has a component in which 37% of the grains provide an age of 11.3 ± 1.43 ka, consistent with the archaeological evidence. For the middle OSL sample (110 cmbs), the most abundant component (44% of the grains) provides an age of 12.5 ± 1.2 ka that is consistent with and overlaps the Younger Dryas onset time frame. The deepest OSL sample (133 cmbs), is stratigraphically older. Its largest component (56% of the grains) provides an age of 22.4 ± 2.3 ka.

**Figure 5:**
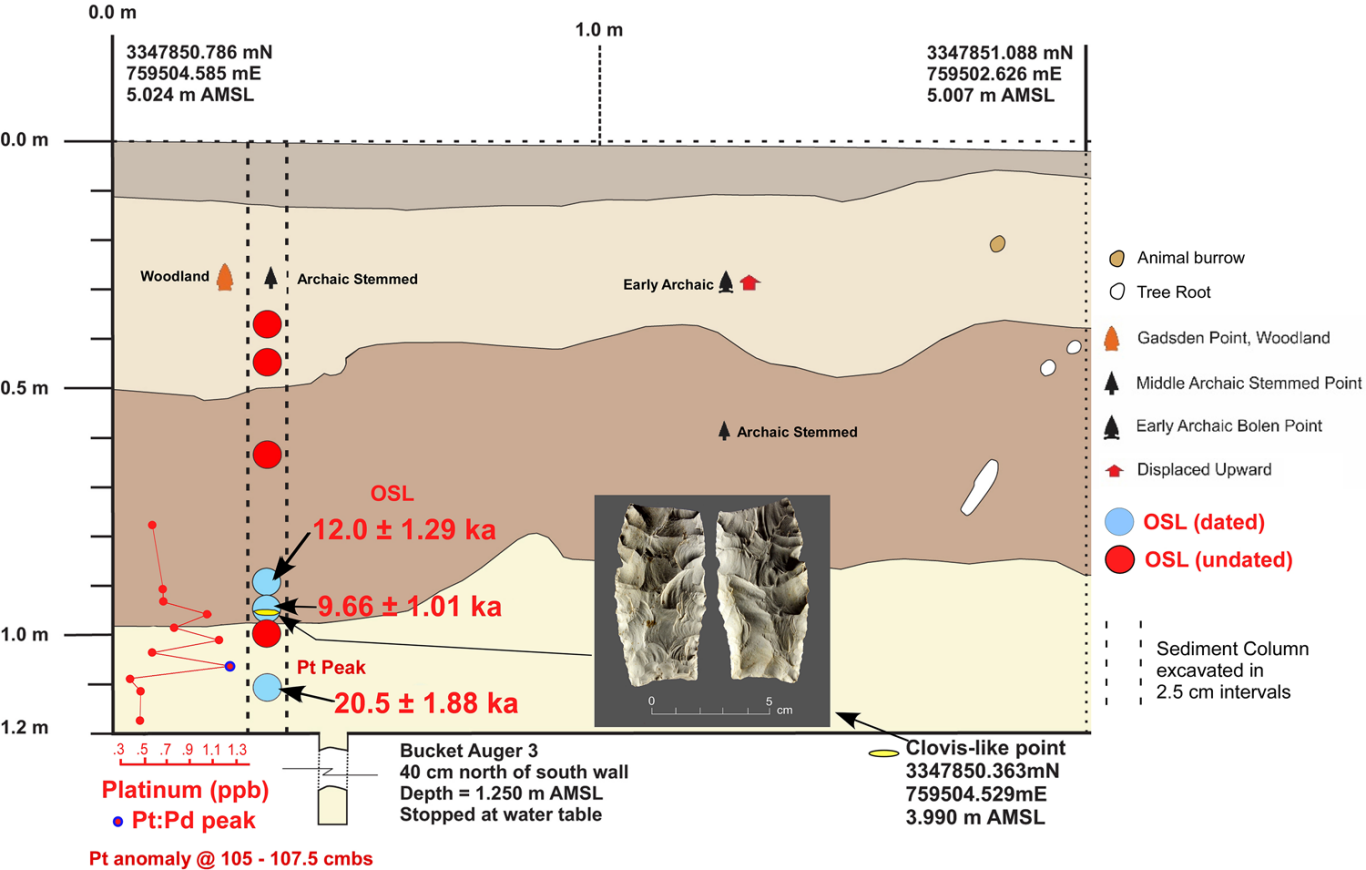
Kennard Unit 4 (KU4) profile at site 8WA329 showing platinum (Pt) abundance in ppb (error =  ± 0.1 ppb) (Supplementary Table 4), OSL age estimates (Table 1), and position of temporally diagnostic artifacts. Sediment zones as observed visually are indicated by color changes.

For KU4, the equivalent dose values of the deeper two samples (96 cmbs and 1.11 m below the surface (mbs) are bimodal in distribution. The shallower sample (at 90 cmbs) has a more continuous distribution. The most likely age estimate for the 90 cmbs sample (12.0 ± 1.29 ka) is based on the central age model (which computes the central tendency of the whole distribution). For the sample at 96 cmbs, the older grain component provides an age of 9.66 ± 1.01 ka (10.67 to 8.65 ka) which is associated with but post-dates a Clovis-like point recovered at that depth (Fig. 5 and Table 1). For both the 90 and 96 cmbs samples, only about 40% of grains are consistent with these ages. The deepest OSL sample (1.11 m below surface) exhibits mixed ages that include a distinctly older component of 20.5 ± 1.88 ka (representing 37% of the grains) and a younger component of ca. 9 ka that is clearly out of stratigraphic order. The 20.5 ka burial age estimate is preferred for the deepest sample due to the stratigraphic consistency for the two younger OSL ages higher in the profile, and the presence of ca. 20 ka OSL dates at similar depths in other excavation units. Very few older grains occur in this sample, suggesting that the 20.5 ka age is the maximum limit. Several grains of this age are even upwardly mixed into the other two younger OSL samples. Discounting these older grains, 50–75% of the grains are consistent with the ca. 10–12 ka ages for the two younger samples.

**Figure 6:**
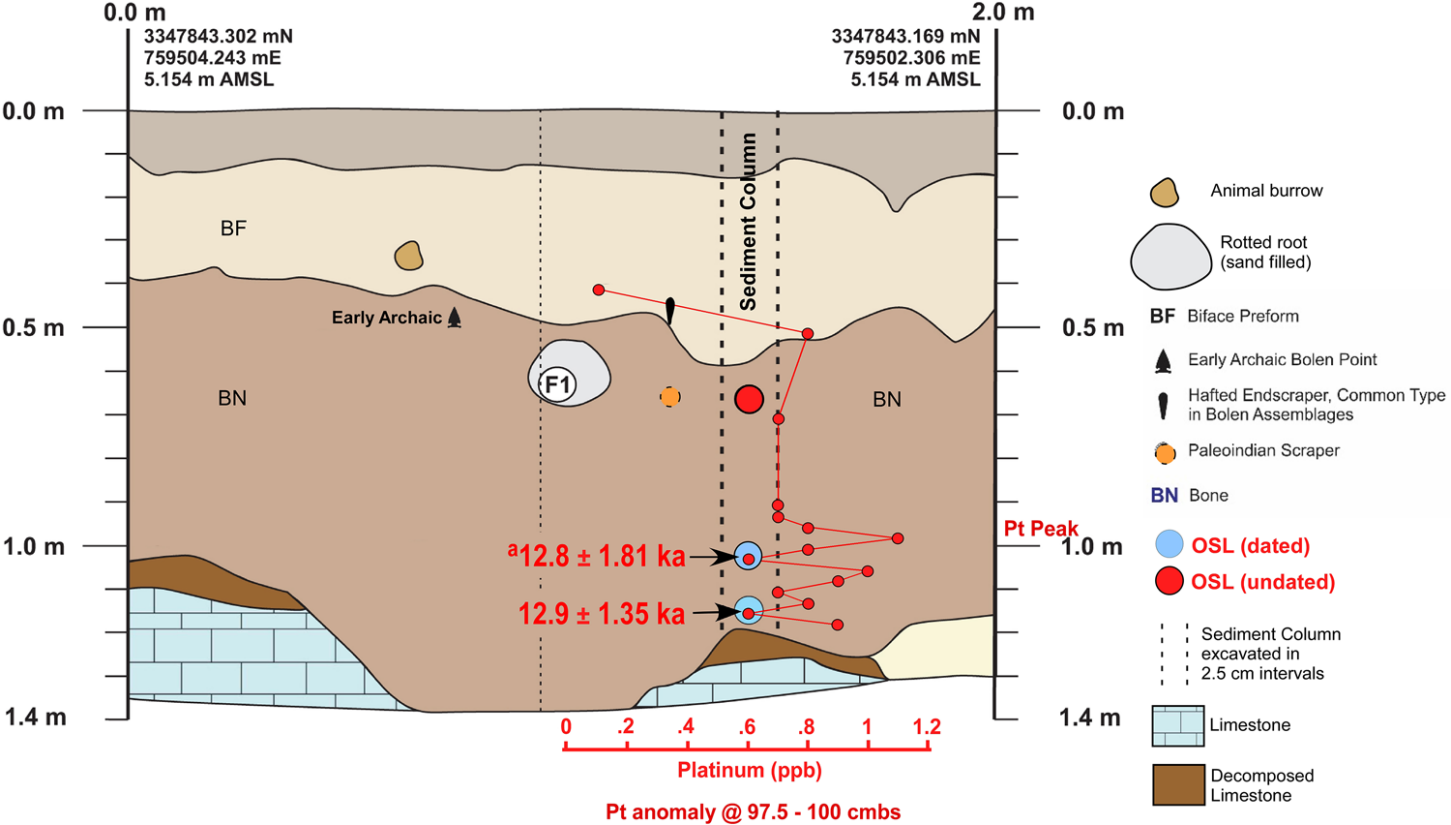
Paleo Unit 15 (PU15) profile at site 8WA329 showing platinum (Pt) abundance in ppb (error =  ± 0.1 ppb) (Supplementary Table 5), OSL age estimates (Table 1), and position of temporally diagnostic artifacts. Sediment zones as observed visually are indicated by color changes. ^a^OSL date is from the adjacent wall of the excavation unit.

For the PU15 sample at 120 cmbs (just above the *in-situ* weathered limestone), the most common component is the oldest, providing an age estimate of 32.6 ± 3.0 ka (Fig. 6 and Table 1), but only accounting for 28% of the grains. However, another component for this sample (20–24% of total grains) provides an age of 12.9 ± 1.35 ka. A second OSL date at 1.025 mbs provides a similar age estimate of 12.8 ± 1.18 ka with 20–24% of the total grains.

**Figure 7:**
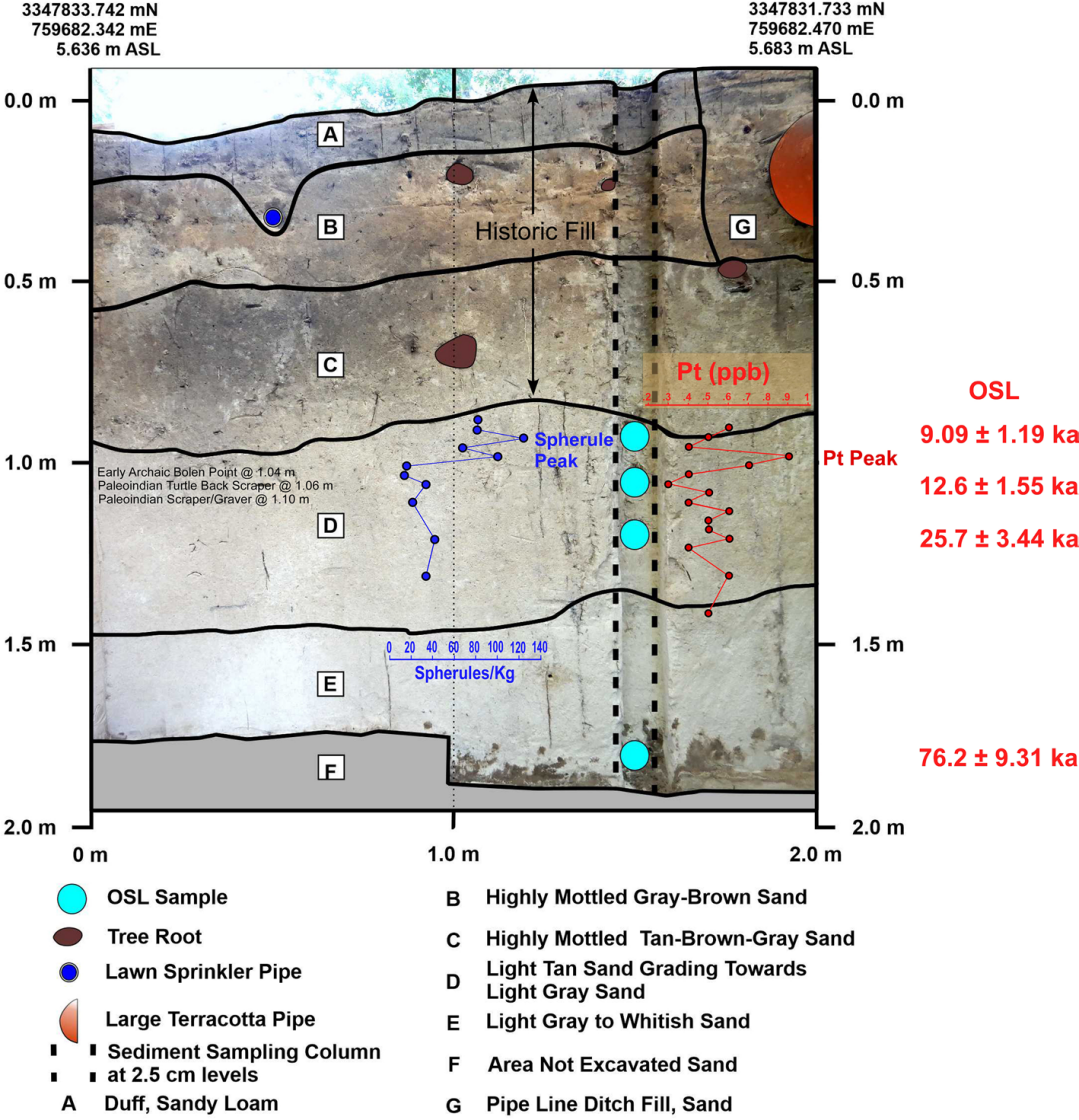
Paleo Unit 23 (PU23) profile at site 8WA329 showing platinum (Pt) abundance in ppb (error =  ± 0.1 ppb) (Supplementary Table 6), OSL age estimates (Table 1), microspherule abundances (spherules/Kg) (Supplementary Table 9), and depths for temporally diagnostic artifacts.

PU23 OSL samples were collected at 90, 103, 120, and 185 cmbs. Most likely burial age estimates are 9.09 ± 1.19 ka for the 90 cmbs sample, 12.6 ± 1.55 ka for the 103 cmbs sample, 25.7 ± 3.44 ka for the 120 cmbs sample, and 76.2 ± 9.81 ka for the deepest sample at 185 cmbs (Fig. 7 and Table 1). The two youngest age estimates are based on the central age model, for which more than 50% of the grains are consistent, while the lower two are based on the most abundant component. OSL age estimates are broadly consistent with the archaeostratigraphy and are in proper stratigraphic order. The 12.6 ka age overlaps the YD onset for the sample collected at 103 cmbs.

OSL samples analyzed for WH1 (8WA1221) include those collected at 35, 100, and 130 cmbs (Supplementary Fig. 15 and Table 1). The sample at 35 cmbs produced a central age model estimate of 2.07 ± 0.25 ka. The sample at 100 cmbs was bimodal and produced ages of 4.25 ± 0.45 and 11.7 ± 1.26 ka. The deepest sample at 130 cmbs was also bimodal and produced age estimates of 9.13 ± 1.17 ka and 27.3 ± 3.4 ka. Based on the depths of OSL ages from 8WA329, ages for the lower two samples at 8WA1221 are ~ 11.7 and ~ 27.4 ka. However, lacking temporally diagnostic artifacts, the true burial age remains unclear. If we accept the youngest age estimates for the lower two OSL samples, this may indicate deep sands at WH1 and suggest that YD age sediments and Paleoamerican artifacts may be deeper than 1.3 m at this location. Only further testing will help to resolve these questions.

## Results

### Platinum chemostratigraphy and archaeostratigraphy of units

Platinum analyses were performed on sediment samples from units KU4, PU1, PU7, PU15, PU23, PU25, and WH1 to test for the presence of a platinum peak anomaly previously widely documented in North American sedimentary sequences in association with the lower Younger Dryas boundary (YDB) dated at ~ 12,800 cal BP (Moore et al*.*^5,6^) (Fig. 2). Based on previous archaeological excavations at Wakulla Springs (Jones^1^; Jones and Tesar^2^), contiguous sediment samples were collected at 2.5-cm intervals from unit profiles spanning the inferred Younger Dryas onset level (ca. 12,835–12,735 cal BP) and stratigraphic levels above and below this. Archaeostratigraphic data and OSL dates provided chronostratigraphic context for inferring the interval bracketing the YD onset and for constraining the interval for contiguous sediment sampling. For PU1, contiguous samples were tested between 90 and 140 cmbs. For PU7, contiguous samples were tested between 95 and 130 cmbs. For KU4, contiguous samples were tested between 90 and 117.5 cmbs, while PU15 contiguous samples spanned 90 to 120 cmbs. Intermittent sample intervals were tested for some units higher in the profile to provide background values for Pt.

The results reveal the presence of a distinct platinum peak in 6 of the 7 unit profiles tested (Supplementary Tables 2–8). A single large Pt anomaly was identified in PU1 at 107.5 to 110 cmbs and in PU7 at 100–102.5 cmbs (Figs. 3 and 4). In KU4, there is a moderate Pt anomaly peak at 105 to 107.5 cmbs and several smaller peaks at the same depth and slightly below a Clovis point variant recovered at 96 cmbs (Fig. 5). PU15 has a moderately high Pt anomaly peak at 97.5 to 100 cmbs and several smaller peaks slightly deeper (Fig. 6). The archaeostratigraphic data and OSL dating suggest that these Pt peaks are all coeval.

**Figure 8:**
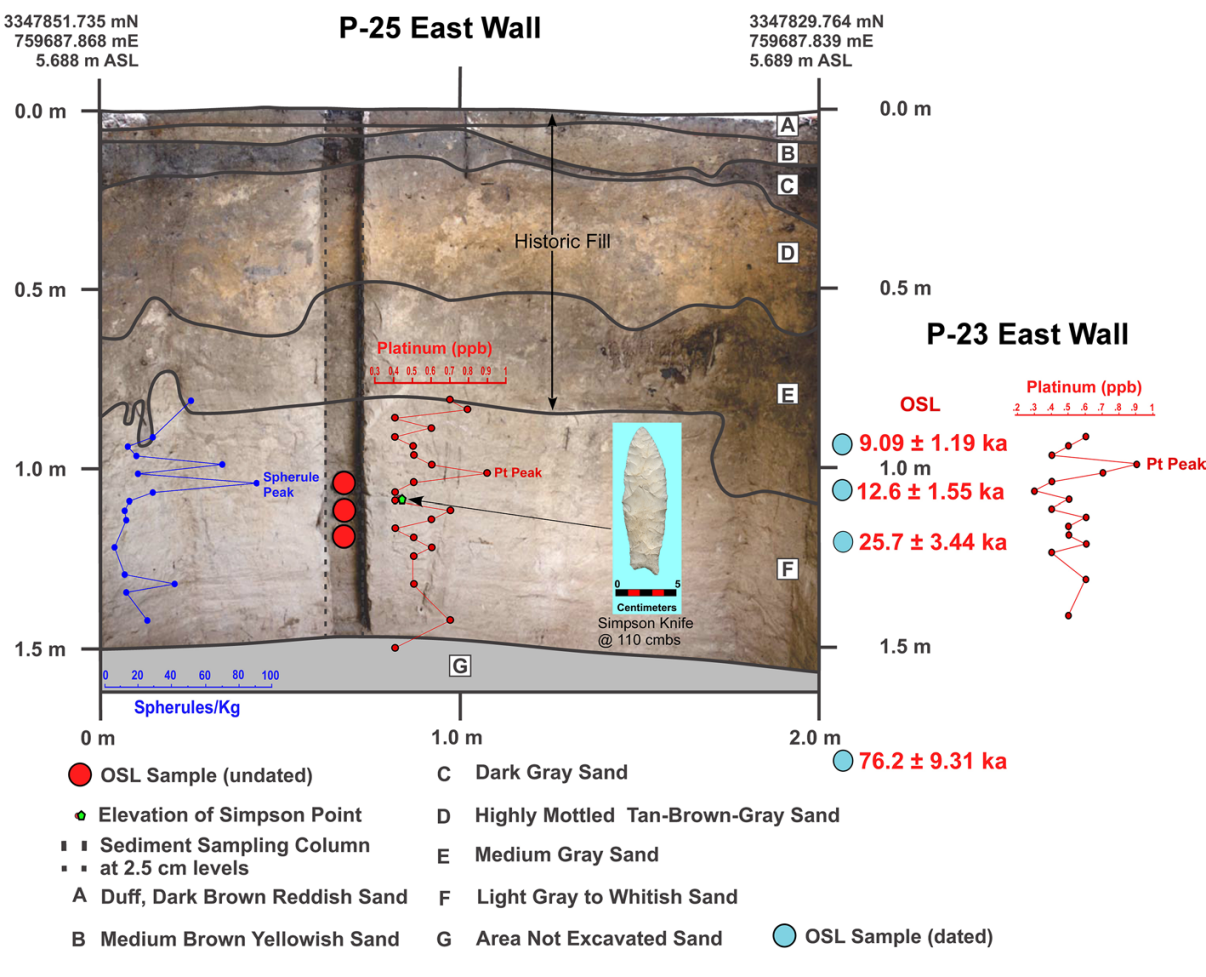
Paleo Unit 25 (PU25) profile at site 8WA329 showing platinum (Pt) abundance in ppb (error =  ± 0.1 ppb) (Supplementary Table 7), OSL age estimates (Table 1), from PU23 (for stratigraphic comparison), microspherule abundances (spherules/kg) (Supplementary Table 10), and stratigraphic position of an *in-situ* Early Paleoamerican Simpson Point.

Two of the units (PU23 and PU25) reveal smaller Pt peaks at depths consistent with the YD onset (Figs. 7–8 and Supplementary Figs. 10–14; Supplementary Tables 6 and 7). OSL dates from PU23 (Table 1 and Fig. 7) provide age estimates that are consistent with the YDB at the same depth as the Pt peak (97.5 to 100 cmbs). PU25 OSL samples were not dated; however, OSL dates for PU23 a few meters away from P25 provide age constraints on the sediments for PU25 (Fig. 8). In addition, sediment geochemistry revealed a moderate Pt anomaly in PU25 at a depth of 100 and 102.5 cmbs, consistent with the Pt peak in PU23. PU25 also has an *in-situ* Paleoamerican Simpson Point recovered ~ 10 cm below the Pt anomaly.

Only one unit studied (WH1, about a kilometer to the south) failed to exhibit a clear Pt peak as in all the other profiles. Instead, this unit revealed minor platinum increases randomly distributed throughout the sequence (Supplementary Fig. 15 and Supplementary Table 8) possibly due to significant post-depositional mixing.

### Fe-rich microspherules

**Figure 9:**
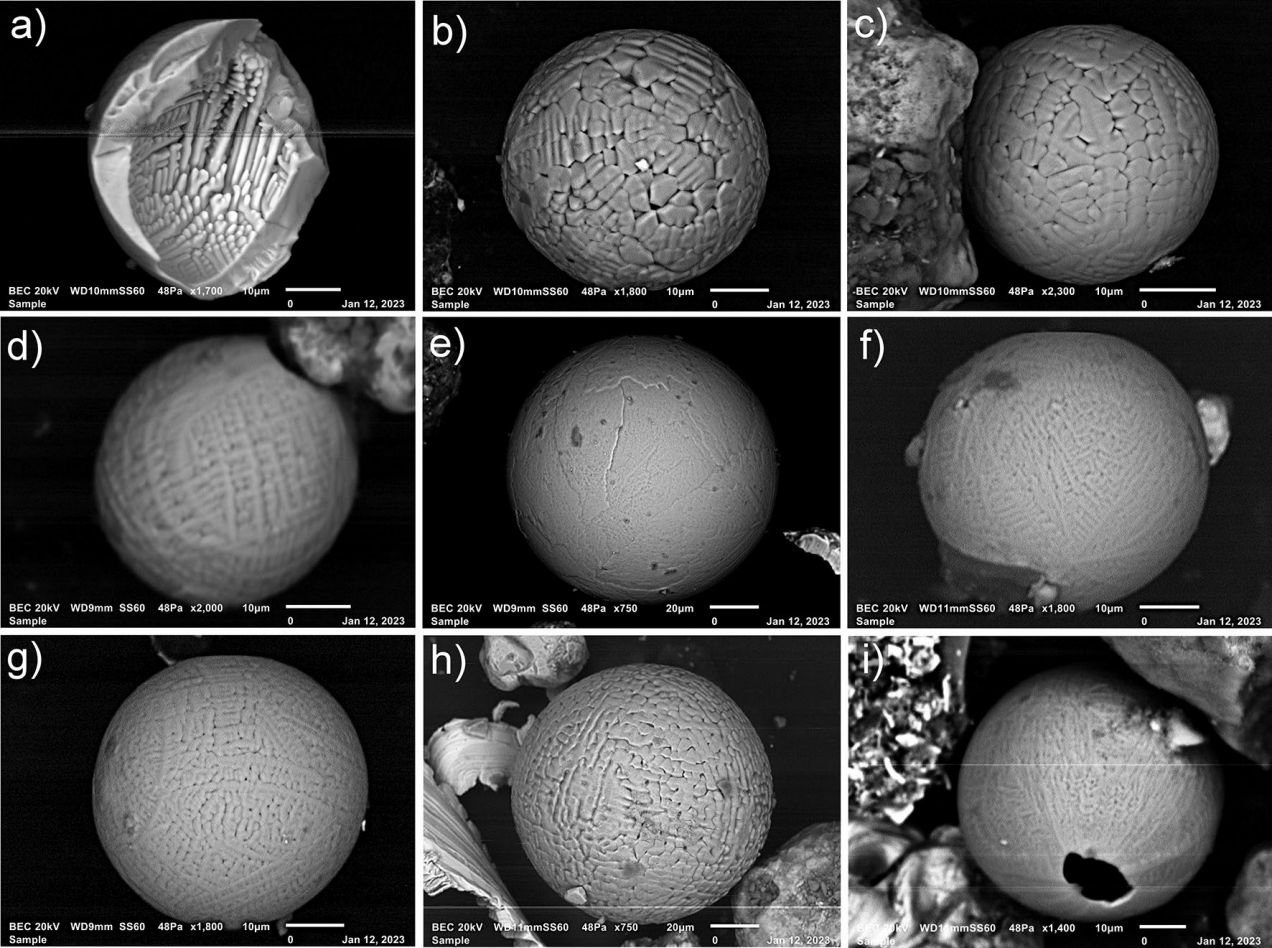
SEM images of iron-rich microspherules (**a**–**i**) from PU25 (102.5—105 cmbs). See “Microspherule EDS Data” in Supplementary Figs. 16–22. EDS elemental map data and point EDS measurements suggest that significant oxygen depletion had occurred on exposed interior surfaces of the broken spherule (Fig. 9a) (Supplementary Fig. 16).

Sediment samples were processed from PU23 and PU25 and examined using a binocular microscope in search of silt to fine sand-sized iron-rich magnetic microspherules. This revealed abundant microspherule peaks in both units at the same depths as the platinum peak anomalies (Figs. 7, 8, 9a–i, 19 and Supplementary Tables 9–10). Much smaller numbers of spherules were found both above and below the spherule peaks. A similar pattern is observed for secondary platinum peaks both above and below the largest Pt peak for several of these units. This is to be expected for highly bioturbated sandy sediment sequences (Moore et al*.*^34^). Analyses of spherules using scanning electron microscopy (SEM) and energy-dispersive x-ray spectroscopy (EDS) show these to be predominantly Fe-oxide–rich (FeO, Fe_3_O_4,_ or Fe_2_O_3_), with dendritic or quench textures due to melting and rapid cooling (Firestone^11^; Wittke^14^; LeCompte^15^). Many spherules are hollow and thin-walled, indicative of outgassing during high-temperature melting. Compositionally, Fe-rich microspherules from Wakulla Springs average 67% Fe, 25% oxygen, with small amounts of Si (average = 0.85%) and Al (average = 0.4%) along with traces of REEs and PGEs (see Supplementary Table 11).

### High-temperature minerals in spherules

Several previous investigations identified various minerals as key indicators in forming high-temperature iron-rich microspherules^12,19,38^. To investigate, we used SEM–EDS to identify any potentially high-temperature minerals. Analyses were performed at two university laboratories, which produced consistent results. We acquired 58 measurements on spherules, all of which were composed of magnetite (Fe_3_O_4_, melting point =  ~ 1590 °C), low oxygen wüstite (FeO, ~ 1590 °C), and titanomagnetite (TiFe_2_O_4_, ~ 1625 °C). The Wakulla spherules were enriched in Pt in 27/58 SEM–EDS analyses (Pt melting point = 1768 °C), Ir (50/58 analyses; 2466 °C), and other PGEs Spherules were also variously enriched in Cr (47/58 analyses; melting point = 1907 °C), Co (12/58 analyses; 1495 °C), and Ni (39/58 analyses; 1455 °C) (See Supplementary Table 11 for EDS data of high-temperature minerals and Supplementary Table 12 for melting points).

### Bayesian analyses

**Figure 10:**
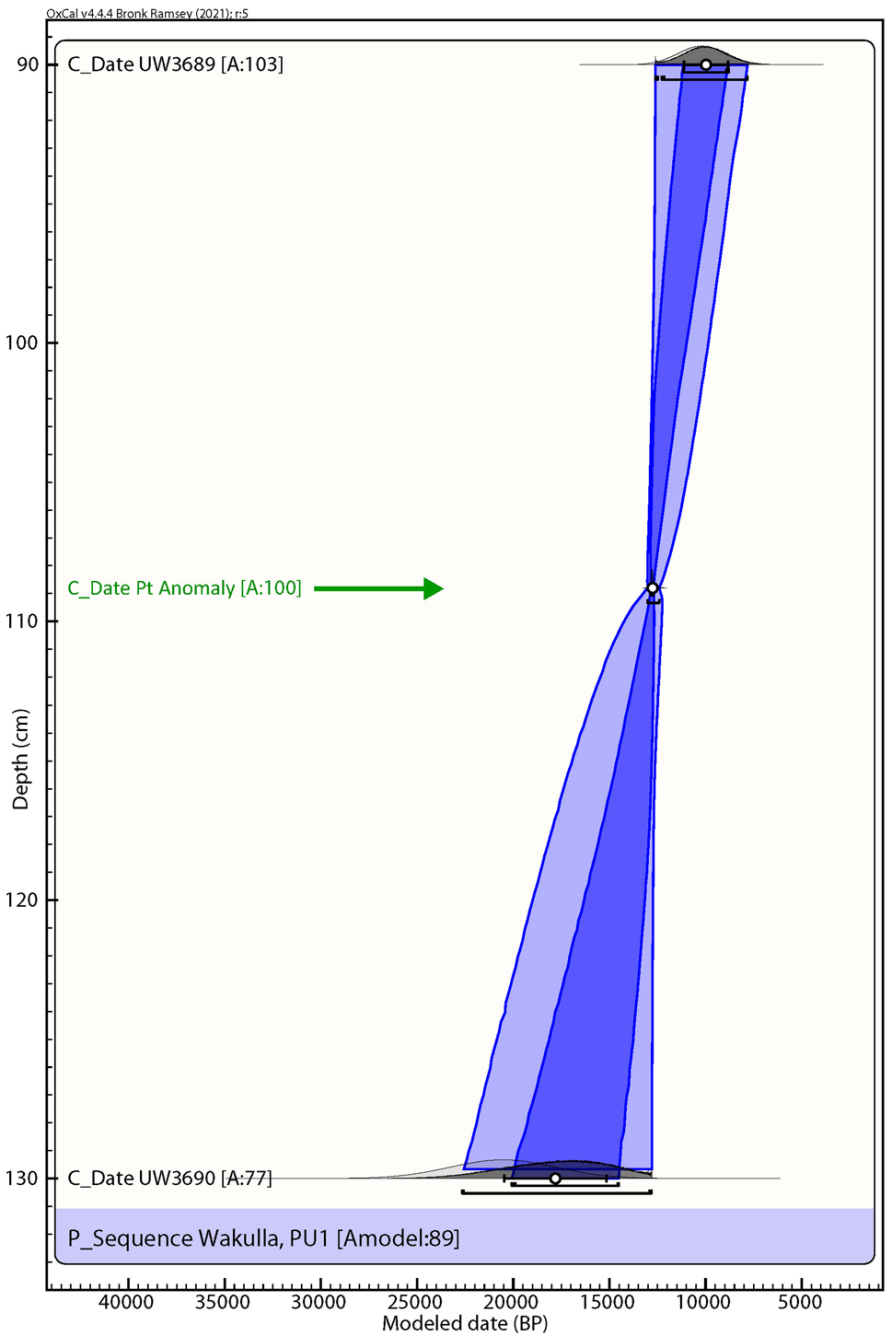
Bayesian analyses to test the utility of YDB Pt anomaly in Paleo Unit 1 (PU1). Age-depth model using 2 OSL dates (black text; Table 1, Fig. 3) and the Pt peak (green text and arrow). The Pt anomaly has a previously determined datum age range of 12,785 ± 50 cal BP (12,835–12,735 cal BP)^4,5^. The Pt peak’s chronostratigraphic position is statistically supported with an Agreement Index of 100 (lower limit ≥ 60), confirming its utility as a chronostratigraphic marker.

**Figure 11:**
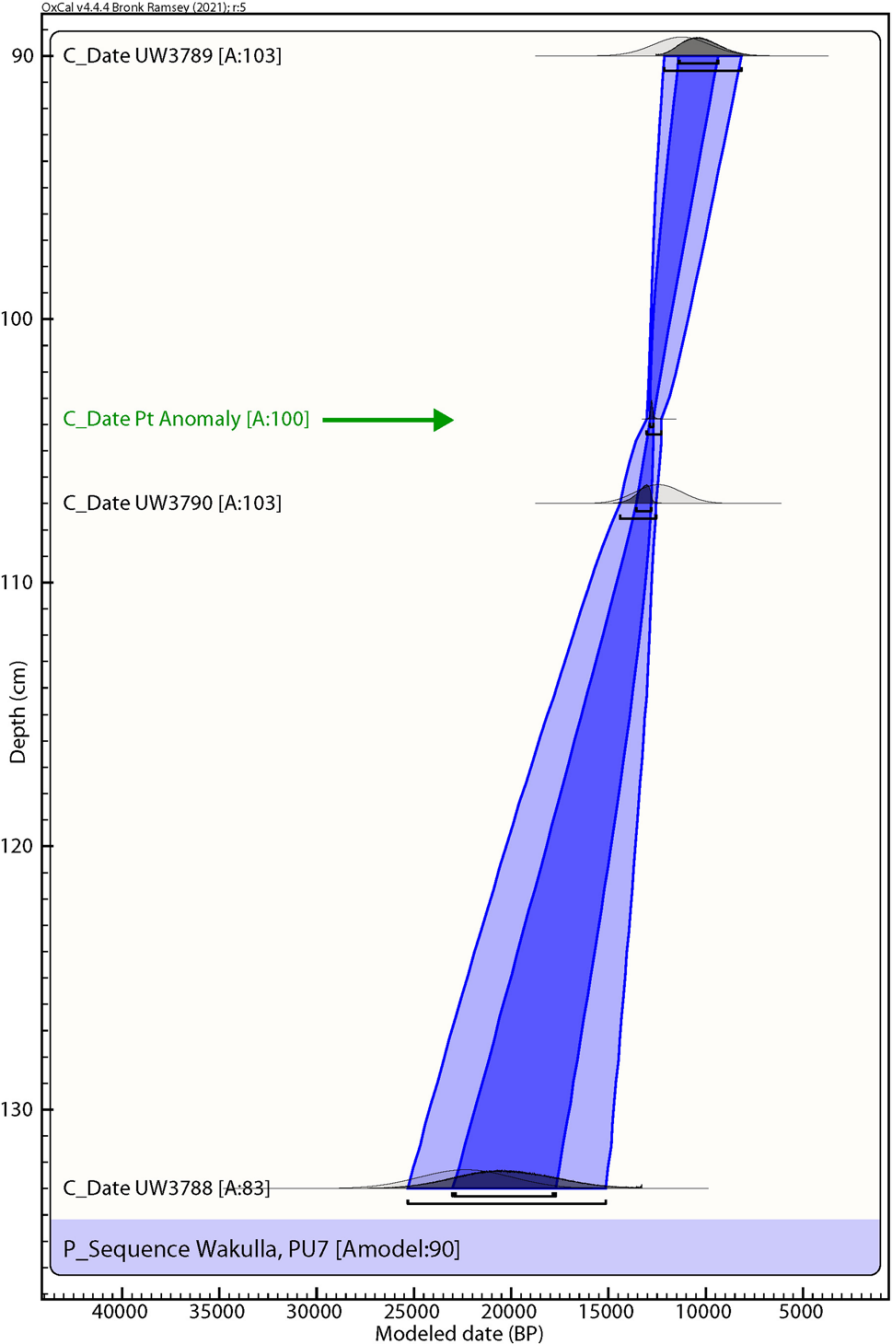
Bayesian analyses to test the utility of YDB Pt peak in Paleo Unit 7 (PU7). Age-depth model using 3 OSL dates (black text; Table 1, Fig. 4) and the Pt peak (green text and arrow). The Pt anomaly has a previously determined datum age range of 12,785 ± 50 cal BP (12,835–12,735 cal BP)^4,5^. The Pt peak’s chronostratigraphic position is statistically supported with a high Agreement Index of 100 (lower limit ≥ 60), confirming its utility as a chronostratigraphic marker.

**Figure 12:**
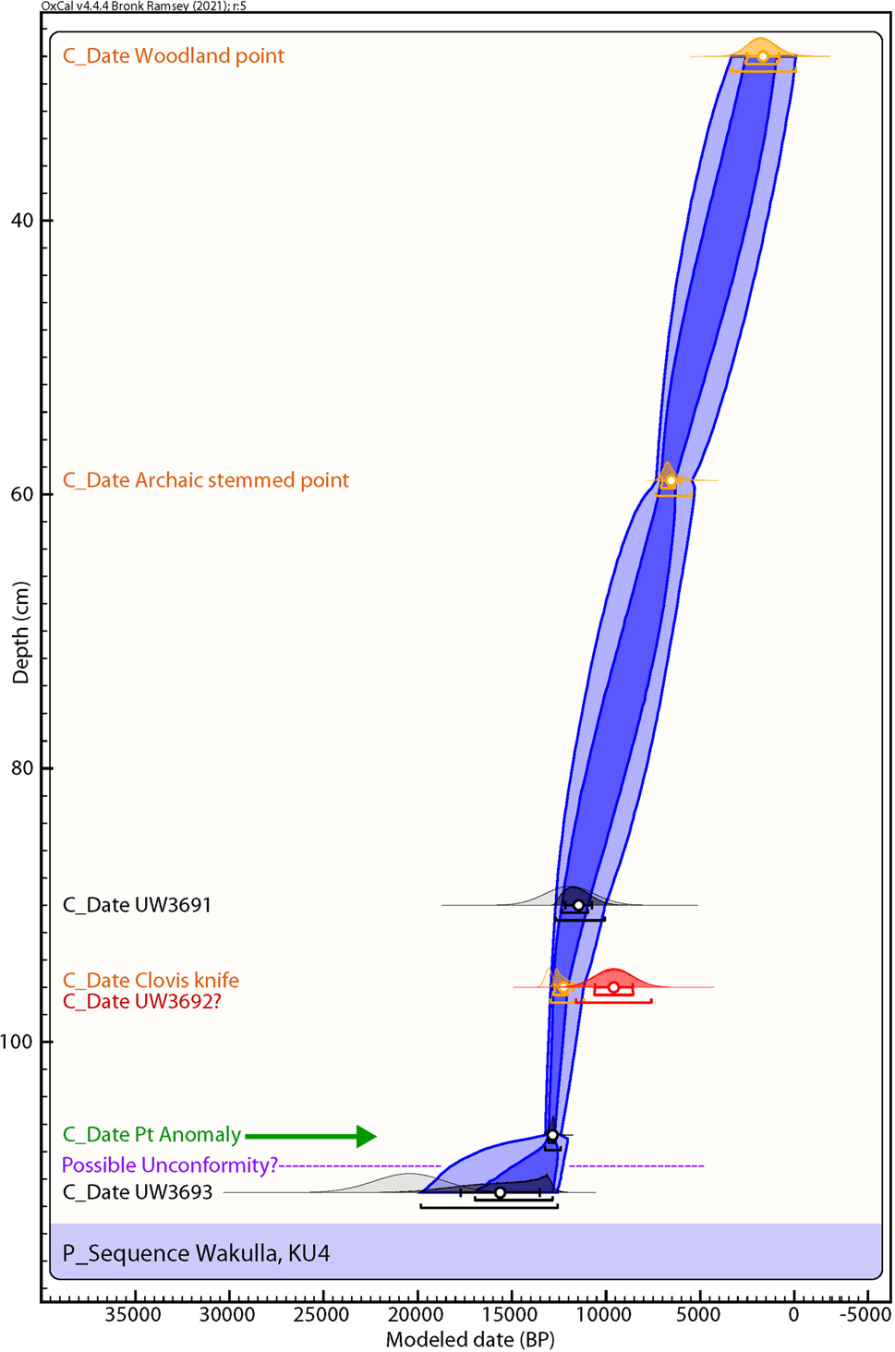
Bayesian analyses to test the utility of YDB Pt peak in Kennard Unit 4 (KU4). Age-depth model using 3 OSL dates (black text; Table 1, Fig. 5), the Pt peak (green text and arrow), and the cultural age ranges of 3 culturally identifiable lithic artifacts (orange text; Fig. 5). OxCal rejected one young OSL date (red) as statistically unlikely (low probability = 10). A probable unconformity (purple text), below the Pt peak is suggested by an OSL date with a low Agreement Index of 36. The Pt peak’s chronostratigraphic position is statistically supported with a high Agreement Index of 98 (lower limit ≥ 60), confirming its utility as a chronostratigraphic marker.

**Figure 13:**
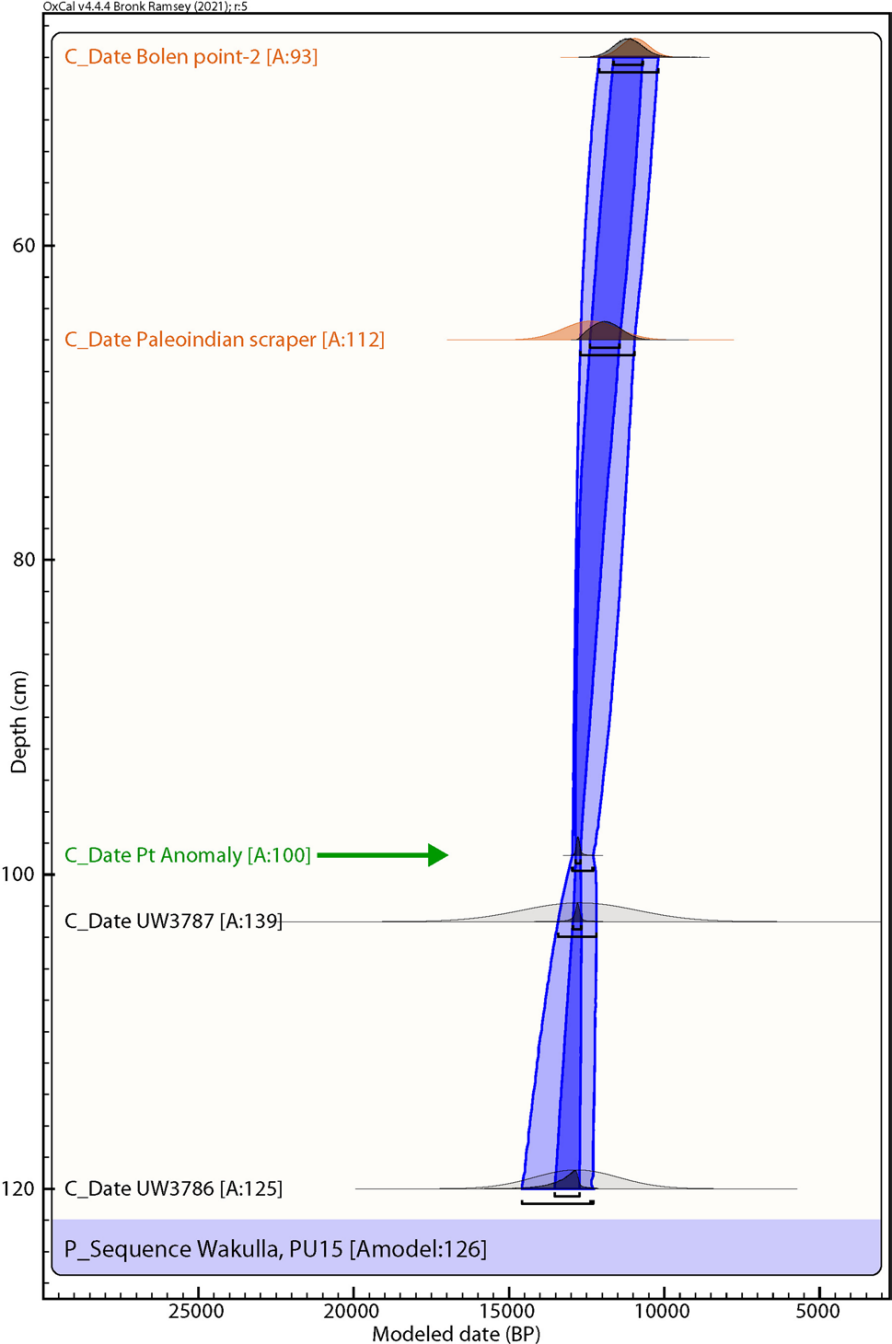
Bayesian analyses to test the utility of YDB Pt peak in Paleo Unit 15 (PU15). Age-depth model using 2 OSL dates (black text; Table 1, Fig. 6), the Pt peak (green text and arrow), and the cultural age ranges of 2 culturally identifiable lithic artifacts (orange-brown text; Fig. 6). The Pt peak’s chronostratigraphic position is statistically supported with a high Agreement Index of 100 (lower limit ≥ 60), confirming its utility as a chronostratigraphic marker.

**Figure 14:**
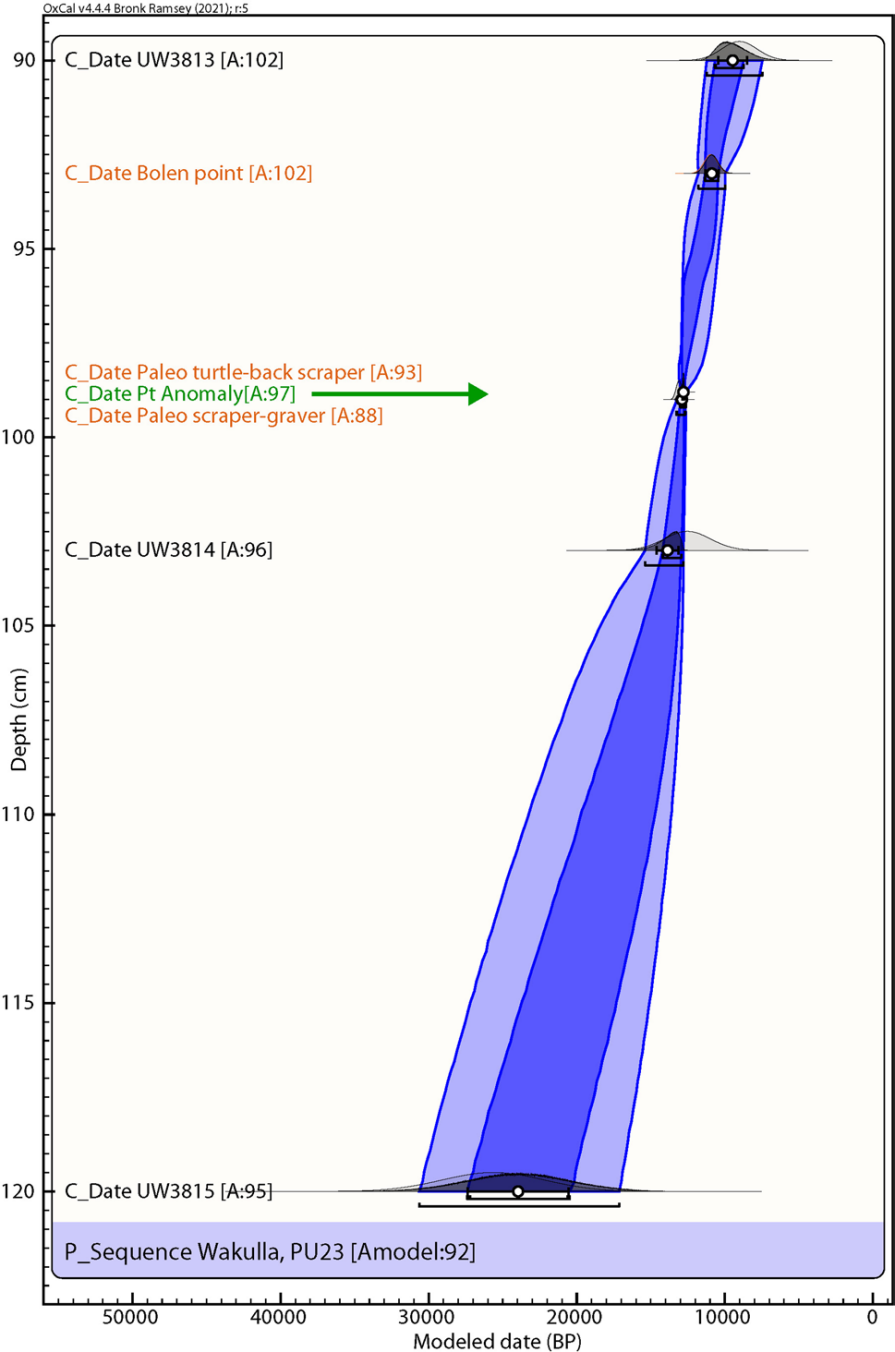
Bayesian analyses to test the utility of YDB Pt peak in Paleo Unit 23 (PU23). Age-depth model using 3 OSL dates (black text; Table 1, Fig. 7), the Pt peak (green text and arrow), and the cultural age ranges of 3 culturally identifiable lithic artifacts (orange text; Fig. 7). The Pt peak’s chronostratigraphic position is statistically supported with a high Agreement Index of 95 (lower limit ≥ 60), confirming its utility as a chronostratigraphic marker.

We used Bayesian analyses to test the hypothesis that the Pt and dendritic microspherule layer may serve as a chronostratigraphic marker (Figs. 10, 11, 12, 13, 14). For five sequences at Wakulla, separated by up to 150 m, we acquired one or more OSL date for each section, collected culturally datable artifacts (lithics), and identified a layer containing anomalously high concentrations of platinum and iron-rich microspherules. We performed Bayesian analysis using the program OxCal v4.4.4, r:5^35^ (InCal20^36^) for each profile. We plotted OSL dates, the age of the Pt anomaly 12,785 ± 50 cal BP (12,835–12,735 cal BP) as determined from GISP2 and other well-dated YDB sites^4,5,37^, and the age ranges of culturally identifiable lithics. The OSL dates had high uncertainties, typically ranging from 1000 to 2000 years, making for low precision and accuracy for the age-depth models produced using OSL alone. In contrast, the Bayesian analyses incorporating the ages of the Pt anomaly and lithics displayed much higher statistical certainty of ± 85–200 years. The Pt peaks’ chronostratigraphic positions are statistically supported by the Bayesian analyses, with a high Agreement Index of 95 or greater for all unit profiles tested (lower acceptable limit is ≥ 60), confirming its utility as a chronostratigraphic marker for the YD onset. The data tables and coding used in the Bayesian modeling, are provided in the *Supplementary Information* “Bayesian Data.” Supplementary Tables 13–17.

### Sediment depositional history of units: granulometry

An understanding of environments of sediment deposition is important for the interpretation of archaeostratigraphy, including determining relative levels of buried occupation surfaces or zones (Moore et al.^34^). This is especially crucial for the relatively uniform sediment sequences of the Southeast Coastal plain that lack observable depositional subdivisions dominated by medium to fine quartz sands with minimal texture differences. Nevertheless, sequences are often marked by either general upward fining or coarsening of sediment sequences that represent discrete depositional episodes. Importantly, the top of each of these depositional episodes represents a former (now buried) surface that may have been available for prehistoric occupation (Brooks and Sassaman^39^).

Below, we briefly describe changes in granulometry data for four unit profiles at Wakulla Springs: KU4, PU1, PU23, and PU25 using bivariate plots of mean grain size and skewness statistical parameters. Bivariate analysis of sand fraction statistical parameters is often employed in search of evidence for stratigraphic integrity for potential subdivisions in sedimentary sequences (Moore et al*.*^34^). Presence of non-overlapping contiguous clusters with changing depth can reveal coherent sedimentary layers that are otherwise visually undetectable. Our statistical analyses of sand and silt/clay fractions and related interpretations of depositional environments are summarized in the *Supplementary Information “Granulometry Data”*.

#### KU4 granulometry

**Figure 15:**
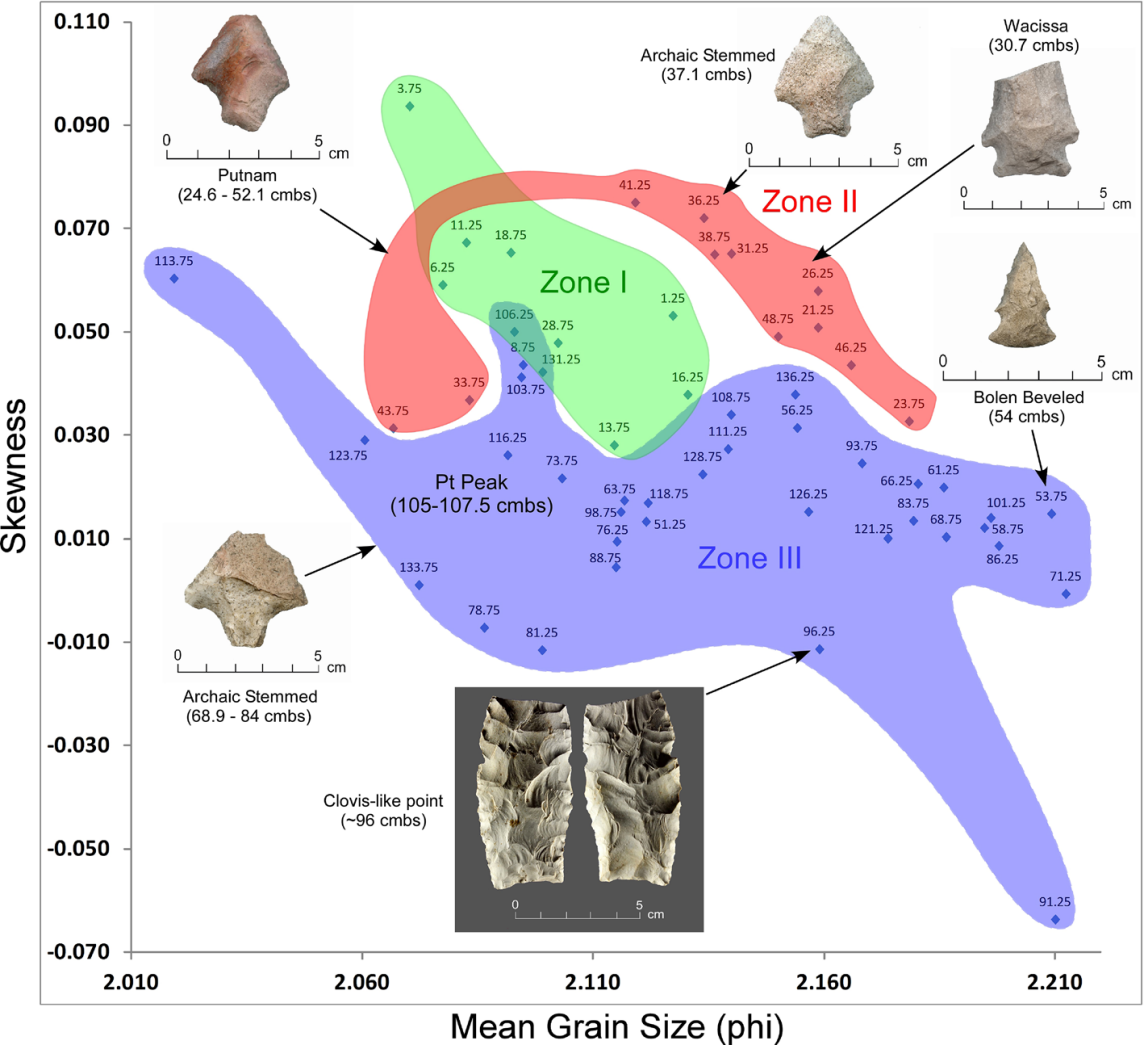
Bivariate plot of mean grain size and skewness for sediments from KU4 at Wakulla Springs (8WA329) showing sedimentary zones and sample depths, the location of the Pt peak, and Late Archaic, Early Archaic, and Paleoamerican temporally diagnostic hafted bifaces. Note: Colored zones represent contiguous samples by depth (cmbs).

A plot of mean grain size and skewness by sample depth (Fig. 15) reveals three broadly definable sediment stratigraphic zones with minimal overlap (Zones I-III). This suggests that sediments within at least portions of KU4 generally maintained their stratigraphic integrity despite post-depositional bioturbation by tree roots, burrowing animals, and/or ants. Supporting this interpretation, temporally-diagnostic artifacts are mostly found to be in the correct chronostratigraphic order, except for an upwardly displaced Early Archaic Bolen point (Fig. 5). The YDB Pt peak in stratigraphic Zone III (Fig. 5) occurs near the base of this excavation unit, ~ 10 cm below a Clovis-like point (Supplementary Table 4).

#### PU1 granulometry

**Figure 16:**
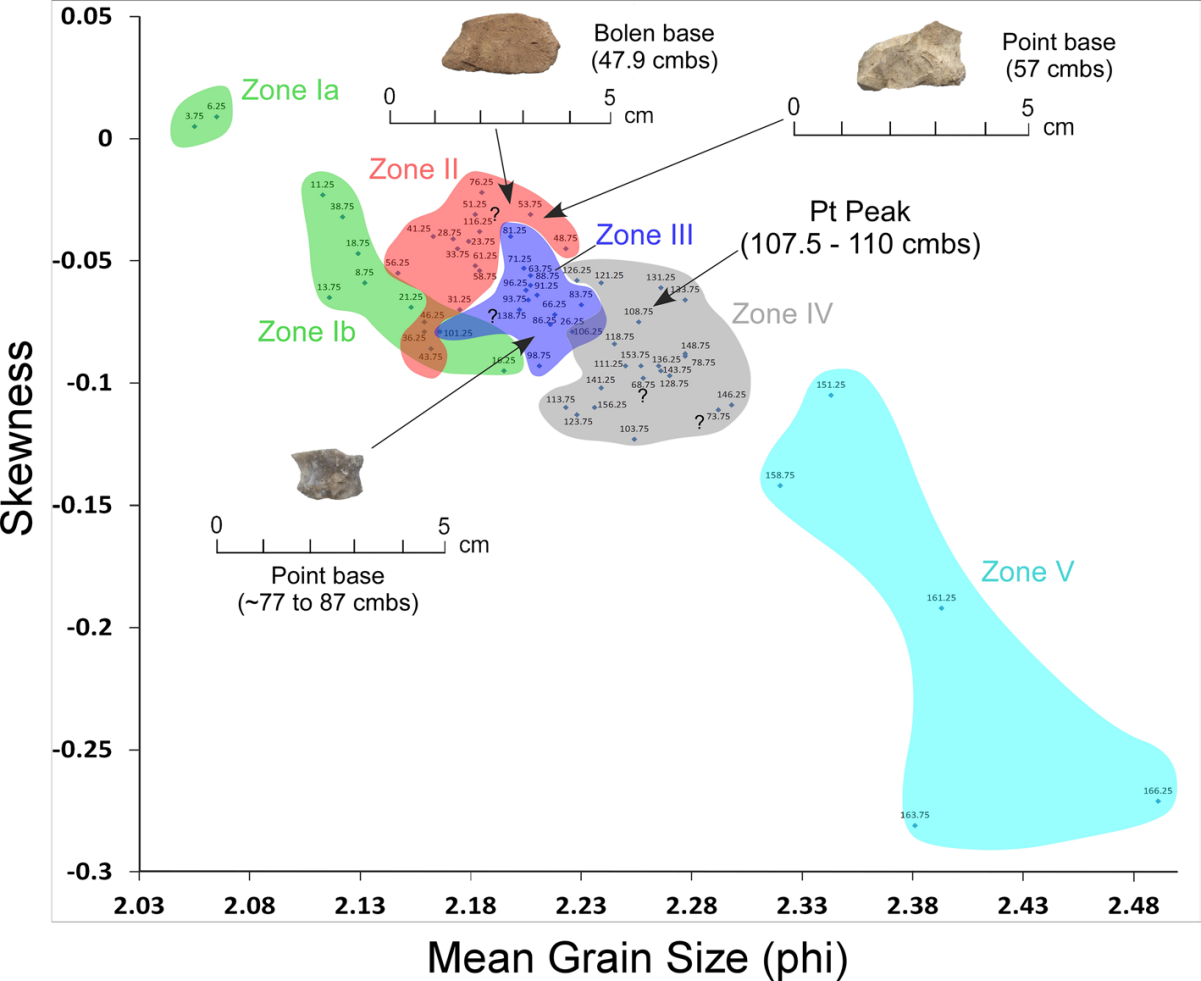
Bivariate plot of mean grain size and skewness for sediments from PU1 at Wakulla Springs (8WA329) showing sedimentary zones and sample depths, the Pt peak, and Late Archaic and Early Archaic temporally diagnostic hafted bifaces. Note: Colored zones represent contiguous samples by depth (cmbs).

A bivariate plot for PU1 of mean grain size and skewness by sample depth (Fig. 16) reveals five broadly definable sediment stratigraphic zones (Zones I-V) exhibiting little overlap. As with KU4, this analysis suggests stratigraphic integrity for PU1, although clear evidence exists for bioturbation and krotovina (filled-in animal burrows). In addition, the vertical distribution of temporally-diagnostic hafted bifaces (Supplementary Fig. 9) provides evidence of artifact displacement. An Early Archaic Bolen Point was displaced upward in Zone II and a small unweathered and heat-treated stemmed point in Zone III was displaced downward suggesting limited reworking. Nevertheless, the multimodal lithic stratigraphic frequencies (Fig. 3) suggest relatively intact archaeostratigraphy, with multiple buried surfaces and/or zones shown by the granulometry and artifact data. PU1 exhibits a platinum peak in Zone IV indicating the YDB layer at ca. ~ 12,800 cal BP (Fig. 3). Overall, the sedimentology, multiple artifact frequency modes by level, and the presence of the Pt peak support stratigraphic integrity in PU1 with limited and spatially-heterogeneous post-depositional disturbance.

#### PU23 and PU25 granulometry

**Figure 17:**
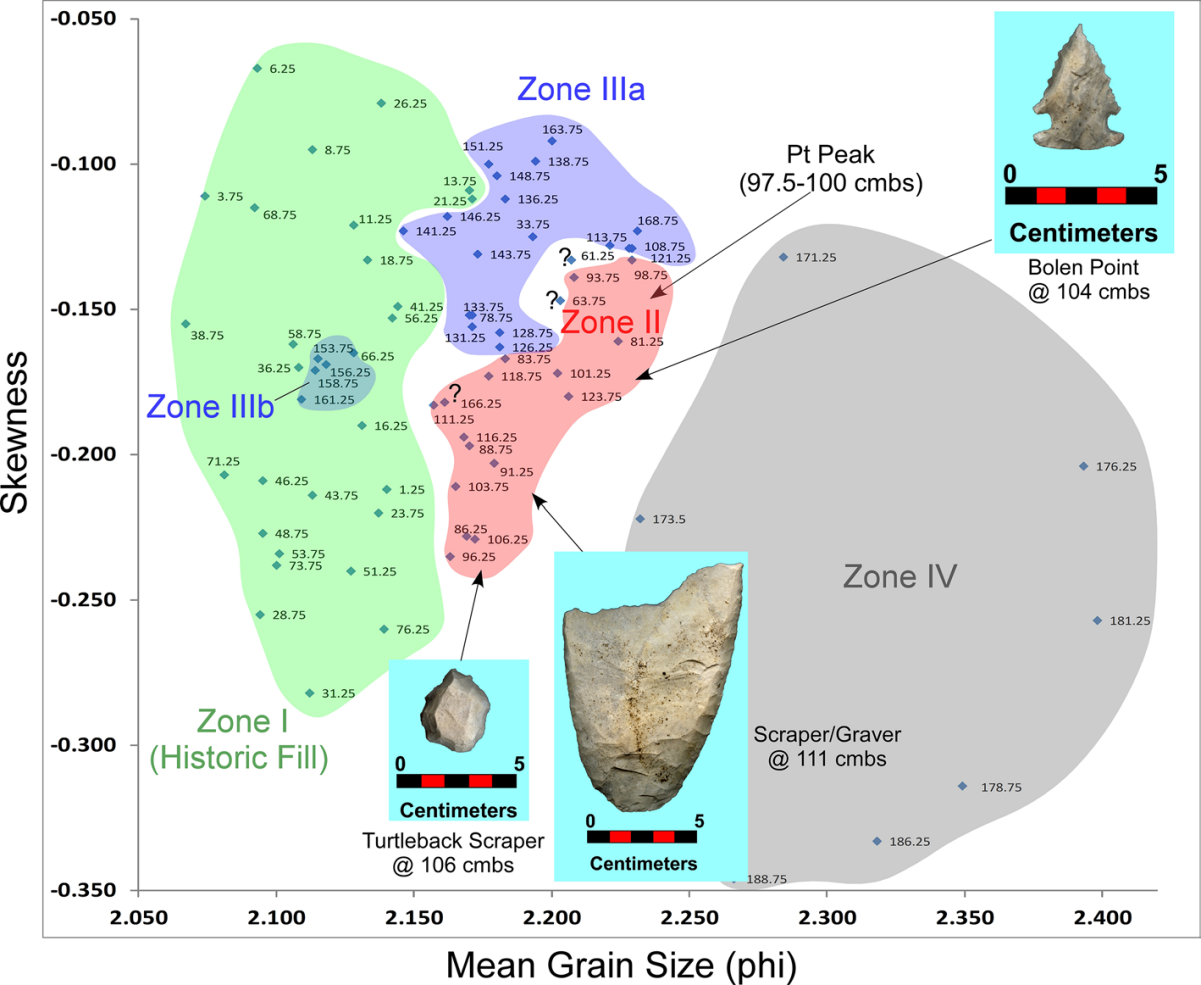
Bivariate plot of mean grain size and skewness for sediments from Paleo Unit 23 (PU23) at Wakulla Springs Lodge (8WA329) showing sedimentary zones and sample depths, Pt peak, and Early Archaic [Bolen Point] and Paleoamerican temporally diagnostic lithic artifacts. Note: Colored zones represent contiguous samples by depth (cmbs).

**Figure 18:**
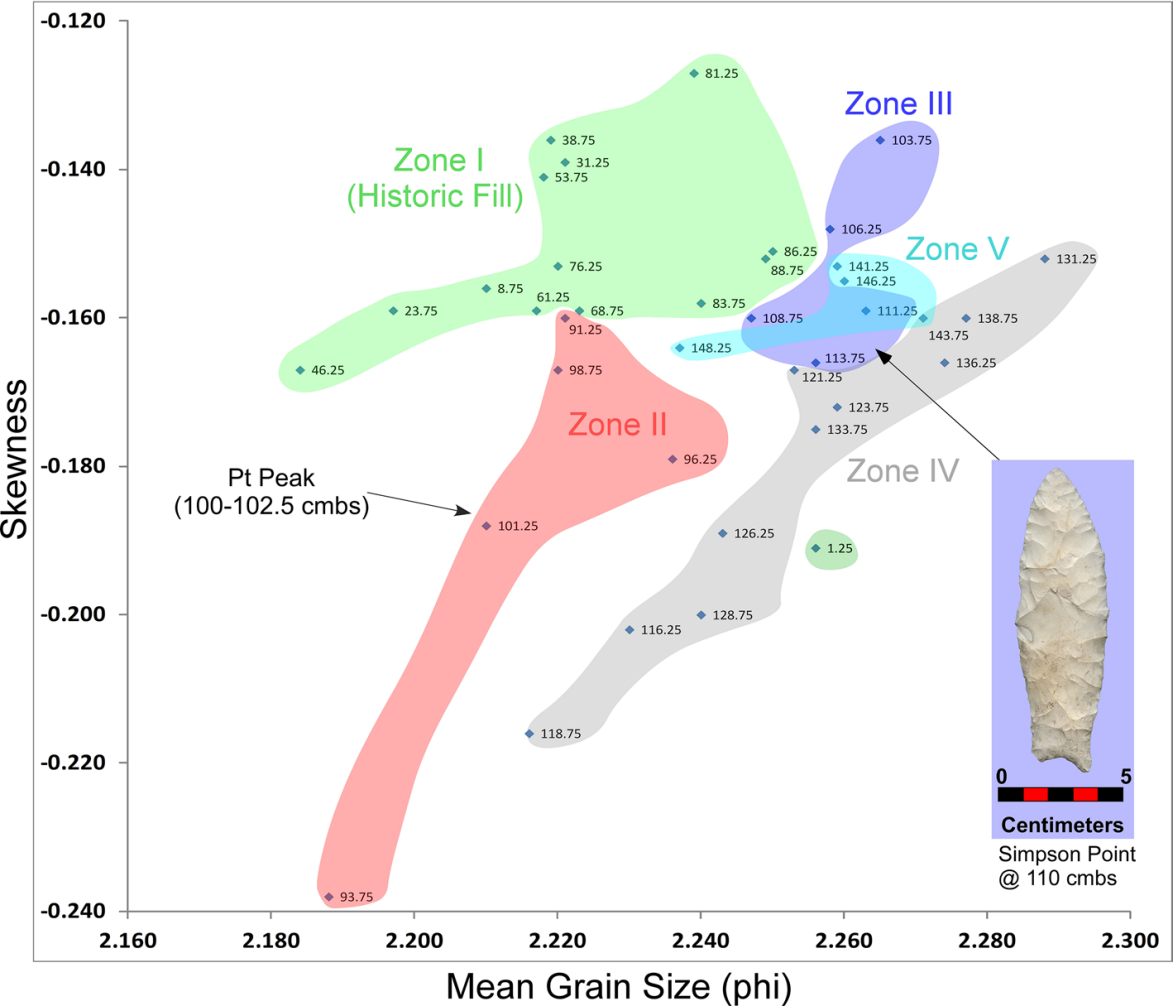
Bivariate plot of mean grain size and skewness for sediments from Paleo Unit 25 (PU25) at Wakulla Springs (8WA329), showing sedimentary zones and sample depths, Pt peak, and location of *in-situ* Paleoamerican Simpson Point at 110 cmbs. Note: Colored zones represent contiguous samples by depth (cmbs).

The bivariate plots for both PU23 and PU25 (Figs. 17 and 18) reveal a sequence of relatively discrete stratigraphic zones. PU23 exhibits 4 broadly definable sediment stratigraphic zones with little overlap (Fig. 17). Zone I represents sediment within the upper ~ 80 cm of modern leveling and fill which are readily distinguished from underlying *in-situ* sediments (Zones II through IV). Zone II represents sediment samples from ~ 80 to 125 cmbs, which contain most artifacts in this unit, including an Early Archaic Bolen Point (104 cmbs), a large Paleoamerican unifacial scraper/graver (111 cmbs), and a Turtleback Scraper (106 cmbs). Zone II (Fig. 17) also exhibits the Pt peak at 97.5–100 cmbs which is close to but slightly offset from an OSL date of 12.6 ± 1.55 ka at 103 cmbs. The well-established age of 12.8 ka for the Pt peak (Petaev et al*.*^4^, Moore et al*.*^5^) suggests that the Bolen Point was displaced slightly downward into older sediments. This interpretation is consistent with the presence of deeply buried Bolen-age animal bone fragments that were likely deposited in intrusive anthropogenic pits. Based on artifact characteristics and distribution, the large unifacial scraper/graver is Paleoamerican in age and occurs close to the same horizon of an *in-situ* Simpson Point in nearby PU25. This observation is also consistent with the depth of an inferred paleo-surface inferred from sediment changes at ~ 110–112.5 cmbs in PU23 and ~ 107.5–110 cmbs in PU25. Both of these artifacts occurred stratigraphically below the Pt peak in each unit.

The bivariate plot of sediments for PU25 reveals 5 relatively discrete stratigraphic zones with minimal overlap (Fig. 18). As with PU23, Zone I sediments represent historic fill produced by leveling of property near the Wakulla Lodge. Zone II, below this historic fill, contains the Pt peak at 100–102.5 cmbs. The granulometry data (Fig. 18), suggests a useful intact stratigraphic sequence indicating that the age of the Simpson Point is earlier than the YD onset (12.8 ka). The Pt peak (in Zone II), located ~ 10 cm higher than the Simpson Point (in Zone III), suggests a potential early Paleoamerican or even pre-Clovis age for the Simpson Point. Further excavations, including a search for additional early Paleoamerican diagnostic hafted bifaces, should assist with testing this interpretation.

## Discussion

Analyses of sediment samples from multiple archaeological test units at Wakulla Springs indicate that elevated platinum concentrations provide a useful chronostratigraphic datum for the onset of the Younger Dryas (12,835 to 12,735 cal BP), as previously established in Greenland ice cores (Petaev et al*.*^4^), North American sedimentary sequences (Moore et al*.*^5,6^), and numerous other studies globally (Pino et al*.*^16^; Moore et al.^19^; Thackeray et al.^40^). Iron-rich microspherules at the Wakulla Springs site are more broadly distributed but have distinct peaks corresponding to the largest Pt peak anomalies.

**Figure 19:**
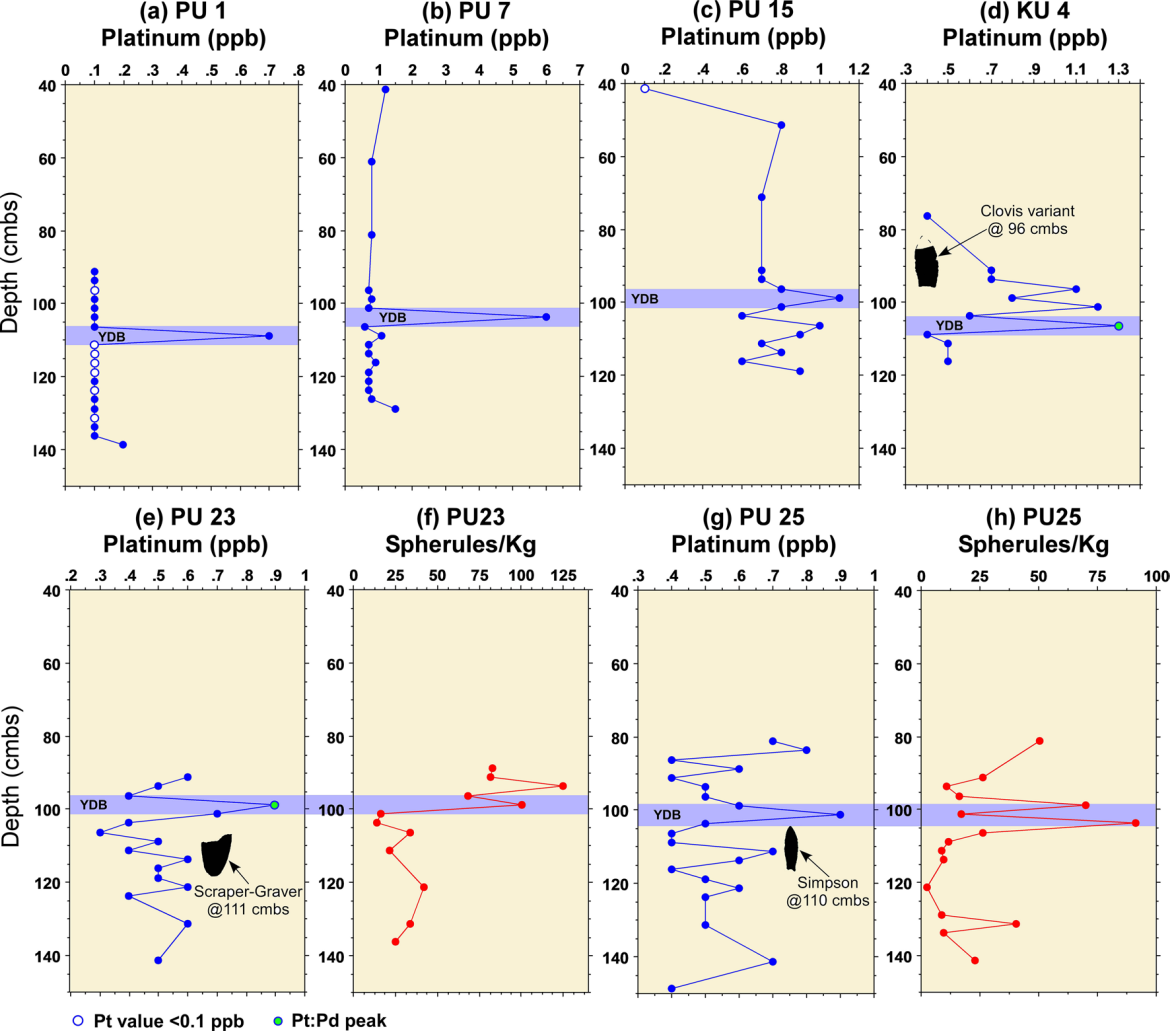
Platinum and microspherule data for 6 Wakulla excavation sequences. Graphs show platinum (Pt) abundance in ppb (error =  ± 0.1 ppb) (**a**–**e**, **g**), iron-rich microspherules/kg for PU23 (**f**) and PU25 (**h**), interpreted level of YDB layer (blue bar), and positions of temporally-diagnostic Paleoamerican artifacts for KU4 (**d**), PU23 (**e**), and PU25 (**g**).

Of the 7 excavation unit sequences tested, 6 exhibit the YDB Pt peak, including two units (PU1 and PU7) with large Pt peaks that are supported with OSL age determinations consistent with a YD onset age (~ 12.8 ka) (Figs. 3, 4, 5, 6, 7, 8, 19). Bayesian analyses of assemblages of OSL dates, platinum peaks, and culturally dated artifacts demonstrate a high Agreement Index of 95 or greater for all profiles tested (minimum acceptable limit of ≥ 60). This finding is in agreement and further reinforces the utility of the widespread Pt anomaly as a chronostratigraphic marker. Depths for Pt peaks average 103 cmbs for all units with only ~ 10 cm of depth variation across all units. Several units (PU15, KU4, and PU25) have smaller, multimodal Pt peaks both above and below the largest Pt peak. These secondary peaks likely resulted from post-depositional bioturbation typical for such sandy sediments; however, in all cases, the highest abundance peak of Pt is consistent with YD onset age based on OSL dating, archaeostratigraphy, and Bayesian modeling. Two of the units were analyzed for iron-rich microspherules and exhibited abundance peaks in close association with the Pt anomalies.

Smaller but still anomalous Pt peaks occur in units PU23, PU25, and PU15. In units PU23, PU1, and PU7, Pt peaks are bracketed by OSL age estimates and archaeostratigraphic data that are consistent with the early Younger Dryas. In addition, analysis of sediments from unit KU4 revealed a Pt peak at 105–107.5 cmbs slightly (10 cm) below the depth of a Clovis-like/Suwannee-type point recovered from that unit in 2017, possibly indicating a small upward displacement of this early Paleoamerican artifact (Fig. 5).

Unit PU25 contains a Paleoamerican Simpson Point ~ 10 cm deeper than the Pt anomaly and may indicate a pre-Clovis age for this variant in Florida, as suggested by Dunbar et al*.*^41^ (Fig. 8). Recovery in 2008 of a Clovis prismatic blade tool at 0.97 m below the surface ~ 10 m away is a consistent age with the other Clovis tools. The Simpson Point in PU25 (Fig. 18) and a large scraper in PU23 (Fig. 17) were both collected ~ 1.1 m below the surface and 10 cm below the Pt anomaly, suggesting that the Clovis and Simpson occupations may have been coeval. However, in other units at Wakulla, the Clovis and Simpson artifacts are separated stratigraphically and chronologically and thus, likely represent two separate cultures. Based on their stratigraphic distribution in our investigation we consider the Simpson Point as being the older of the two. An early temporal placement for Simpson Points in Florida as suggested here indicates that the Vickery Mastodon fossil (Vickery Mastodon) recovered from the nearby Wakulla River (radiocarbon dated to ~ 13,500 cal BP) may be associated with a pre-Clovis Simpson occupation of the springs (Hemmings and Dunbar^42^).

Depths for Pt anomalies vary between ~ 97.5 to 110 cmbs (a range of only 12.5 cm) between all units tested (Fig. 19). Platinum peaks are present in PU1 at 107.5 to 110 cmbs, PU7 at 102.5 to105 cmbs, PU15 at 97.5 to 100 cmbs, KU4 at 105 to 107.5 cmbs, PU23 at 97.5 to 100 cmbs, and PU25 at 100 to 102.5 cmbs. Using the Pt peak as a YDB datum (~ 12.8 ka), average sedimentation rates for each unit range between 0.78 and 0.86 cm of deposition per century. Actual sedimentation rates likely varied considerably, with bursts of eolian and slopewash sedimentation occurring during droughts or periods of excessive precipitation associated with climatic shifts (Moore et al*.*^34^) Given the distance between the excavation units of 2017 and the Wakulla Lodge units of 2018 (Fig. 2), these depths and sedimentation rates are remarkably similar and indicate a level of stratigraphic continuity between excavations and across the landform despite historic land clearance, leveling, and varying topography. It also indicates that future excavations at the Wakulla Lodge are likely to encounter Clovis and pre-Clovis artifacts at depths below ~ 1 m to as deep as 1.5 m depth as Calvin Jones identified in 1995 (Jones^1^ and Jones and Tesar^2^). OSL ages also indicate that in most areas, sediments deeper than ~ 1.3 to 1.5 m below the modern surface are especially old (pre-LGM). The sequences at Wakulla Springs clearly exhibit evidence of vertical sediment mixing based on displaced artifacts and temporally multiple ages of sand grains. Nevertheless, relatively solid archaeostratigraphic integrity, and distinct sedimentary zones (Figs. 15, 16, 17, 18) may reflect changes in the landscape and associated sedimentary processes. These changes likely resulted from millennial-scale climatic changes with episodes of eolian sand sheet deposition (e.g., PU23 and PU25) and localized slope-wash reworking (the KU units near the slope), followed by episodes of landform stability, and subsequent human occupation^34^.

The presence of broad peaks in iron-rich microspherules at or close to platinum peaks for PU23 and PU25 (Fig. 19) is consistent with previous Younger Dryas Impact Hypothesis (YDIH) studies showing dendritic, Fe-rich spherules distributed within stratigraphic units globally dated to the YD onset (e.g., Firestone et al*.*^11^; Wittke et al*.*^14^). Along with the platinum anomalies, the presence of iron-rich microspherules is consistent with previous evidence for an extra-terrestrial impact of a fragmented comet and atmospheric input of platinum-rich dust and predominantly silt-sized microspherules in addition to other reported impact related proxies between 12,835 and 12,735 cal BP (Kennett et al*.*^37^). As discussed by Moore et al*.*^5,6^, the platinum peak anomaly has demonstrated utility as a precise chronostratigraphic marker for the YD onset in sedimentary sequences across North American, South America, South Africa, and Eurasia (Moore et al.^5,6^; Pino et al*.*^16^*;* Thackeray et al.^40^; Moore et al*.*^5,6^). This finding is now replicated for the first time from multiple archaeological sequences at Wakulla Springs, Florida that also display temporally correlated microspherule peaks.

While microspherule peaks are present and coeval with Pt anomalies, the microspherules at Wakulla Springs are more broadly dispersed in lower abundances (Fig. 19f,h) above and below the YD onset due to post-depositional bioturbation and pedogenic modifications (i.e., illuviation/leaching) in sandy sediments that lack strongly developed soil structure. In contrast, the presence of more stratigraphically restricted platinum peaks implies less stratigraphic mobility for nano-scale, platinum-rich dust likely bound up in thin clay skins on sand grains. This argues for the use of platinum testing over searching for microspherules as a rapid and effective approach to locate the chronostratigraphic position of the YD onset, both for paleoenvironmental reconstructions and use in early Paleoamerican studies. Nevertheless, the stratigraphic distribution of microspherules remains a valuable additional proxy for identifying the YD onset, including in sequences where Pt data is not available.

The stratigraphic distribution of Pt anomalies for identifying the YD onset (ca. 12.8 ka) has proved invaluable at Wakulla Springs for inferring the relative chronostratigraphic levels for Clovis versus the Suwannee/Simpson technocomplex and provide a new archeological tool for evaluating the relative age of other Paleoamerican morphometric subtypes common in Florida.

### Origin of high-temperature minerals in microspherules

Here, we consider and compare alternate potential explanations to account for the high-temperature minerals in microspherules recovered from the Wakulla units. These include anthropogenesis, biomass burning, and cosmic impact.

#### Formation by anthropogenesis

In the investigation of dozens of different types of fly ash produced anthropogenically, it was found that Cr is typically enriched < 3 ppm^43,44^, compared to an average of 16,000 ppm (1.6 wt%) for Wakulla, a concentration more than 5000 × larger. Ni in fly ash was at < 550 ppm^43,44^, compared to 6300 ppm (0.63 wt%) at Wakulla. The maximum Pt concentration reported was 0.25 ppm, compared to 1209 ppm at Wakulla, 4800 × larger than fly ash^45^. Concentrations of these minerals are typically low in anthropogenic spherules, such as fly ash^8,9,12,19,38^ thus, it seems unlikely that the Wakulla microspherules are fly ash, and of anthropogenic origin.

#### Formation by biomass burning

Moore et al*.*^19^ investigated the formation of melted YDB spherules by biomass burning, including haystack fires and midden fires. In biomass-burning samples investigated, they observed numerous low-temperature melted grains, including plagioclase and feldspar, with melting points of ~ 1200 °C, consistent with a temperature range of 1155–1290 °C^46^, approximately the melting point of bulk sediment (Supplementary Table 12). However, no Fe oxide microspherules were found which is of significance because these melt at > 1590 °C. This observation suggests that Wakulla microspherules did not form during biomass burning.

#### Formation by cosmic impact

When present in microspherules, minerals containing high concentrations of Cr, Ni, Co, Pt, Ir, and the other PGEs are commonly proposed to represent markers of extraterrestrial events^8,9,12,47–50^. These elements have melting points ranging from 1430° to 2466 °C (Supplementary Table 12), much higher than for melt products of anthropogenesis and biomass burning. Furthermore, the presence of high concentrations of these high-temperature elements is consistent with the Pt and dendritic microspherule abundances considered of impact origin at other sites^6–8^. In addition, wüstite, an unstable, low-oxygen form of iron oxide that tends to oxidize rapidly, is a highly reduced mineral that rarely occurs under natural terrestrial conditions. However, wüstite is common in meteorites and materials produced during impact events under oxygen-deficient conditions^12,16,38^.

Thus, the most plausible hypothesis that matches the evidence is that Wakulla microspherules include cosmic impactor material (e.g., Pt, Ir, Cr, Co, Ni, and wüstite). The peak concentration of sedimentary Pt is closely associated with Pt-enriched microspherules, supporting Pt’s proposed utility as a chronostratigraphic datum.

## Conclusions

A peak abundance anomaly of platinum and iron-rich microspherules has previously been proposed as a chronostratigraphic marker for the lower Younger Dryas Boundary (YDB) dating to ca. 12,835 to 12,735 cal BP. In this study, we tested this hypothesis through analysis of multiple units of sandy, weakly stratified sediment at Wakulla Springs, Florida in search of YDB peaks in platinum and microspherules. In multiple sediment sequences, our work revealed distinct abundance peaks in platinum and dendritic microspherules, found at other sites to mark the YD onset (12.8 ka), thus providing a valuable chronostratigraphic datum. The ~ 12,800-year-old age of the Pt anomaly is statistically supported by Bayesian age/depth modeling with high Agreement Indexes of 95 or higher. Ages inferred for the multiple Wakulla Springs units are based on this chronostratigraphic datum and are generally supported by the archaeological data and OSL dating. This datum has proven useful for intersequence correlation and for assessing relative ages of Paleoamerican artifacts, including those of likely Clovis, pre-Clovis, and post-Clovis age, in addition to evaluating responses to environmental changes known to have occurred during the Younger Dryas climatic episode. OSL dating results are also useful in the determination of the degree of post-depositional bioturbation and therefore also have geoarchaeological value beyond its original intended use.

Post-depositional mixing of sediments through bioturbation may blur platinum and microspherule peaks; however, platinum peaks, when present, appear to be a robust chronostratigraphic datum of the YD onset. We speculate that this is because nano-scale platinum-rich dust is pedogenically bound-up in thin clay coatings on sand grains. In particular, the coarser fraction of sand appears likely to be less mobile stratigraphically due to bioturbation that tends to transport fine sands both higher and lower in the sandy sediments. This size-sorting of different sized sand grains may also explain why multiple age populations of sand grains (indicating mixing) are revealed by the OSL analyses which use the fine sand fraction (i.e., 180–212 micron) for dating. Microspherules appear more mobile stratigraphically due to their sizes which range from coarse silt to fine sand, but still, they broadly correlate with platinum peaks.

In this study, the platinum and microspherule peaks suggest that Paleoamerican Simpson Points may be either coeval with Clovis or pre-Clovis in age and not post-Clovis as has been assumed. Further work will be needed to confirm this, including additional testing of sediment sequences for platinum and microspherules peaks at other sites in Florida. In summary, our work at Wakulla Springs confirms the utility of abundance peaks in platinum and microspherules as a robust chronostratigraphic datum, approximately marking the onset of the Younger Dryas climate episode ~ 12,800 years ago.

## Materials and methods

### Geochemistry (Pt, Pd, Au)

For geochemical analysis, sediment samples were collected in the field directly from cleaned archaeological unit profiles, stored in plastic bags, and allowed to air dry over several days. Bulk sediment samples then were dry sieved using a 63-micron sieve to separate the silt and clay fractions from the sand. The silt and clay fraction were then weighed and repackaged in a plastic bag for shipment to Activation Laboratories Inc. (Actlabs) for “1C-Research” analysis. Sediment samples of ~ 50 g are required for the "1C-Research" analysis.

Activation Laboratories Inc. (Actlabs), used fire-assay (FA) and inductively coupled plasma mass spectrometry (ICP-MS) following Hoffman and Dunn^51^, to measure elemental concentrations of the sediment samples from all sites. Before analysis, each sample is mixed with fire assay fluxes (borax, soda ash, silica, litharge), and silver (Ag) is added as a collector. The mixture is placed in a crucible and preheated at 850 °C, intermediate at 950 °C, and finished at 1060 °C for a total of 60 min. After the crucibles are removed from the assay furnace, the molten slag is poured into a crucible leaving a lead button, which is then preheated to 950 °C to recover the Ag (doré bead) plus Au, Pt, and Pd.

The Ag doré bead is digested in hot (95 °C) HNO_3 _+ HCl with a special complexing agent to prevent the Au, Pd, and Pt from adsorbing onto the test tube. After cooling for 2 h the sample solution is analyzed for Au, Pt, and Pd using a Perkin Elmer Sciex ELAN 9000 ICP-MS. On each tray of 42 samples, there are 2 method blanks, 3 sample duplicates, and 2 certified reference materials. The ICP-MS is recalibrated every 45 samples. Smaller sample splits are used for high chromite or sulfide samples. Measurements are reported in parts per billion (ppb) with a lower limit of detection for Pt at 0.1 ppb.

### SEM–EDS

SEM–EDS analyses were conducted using a JEOL-6000 SEM system at Elizabeth City State University and a ThermoFisher Apreo 2 at the University of Oregon (Supplementary Table 11). For EDS wt% > 1%, uncertainties are ± 10%. For wt% ≤ 1%, uncertainties are approximately ± 50%.

### Luminescence dating (OSL)

OSL samples were collected in light-tight containers (i.e., 2-cm-diameter copper tubing) taken from cleaned sediment sequence exposures. Sample tubes were removed and capped in the field along with bulk moisture/dose rate determination samples taken from each sample location. All samples for luminescence dating from 8WA329 (*n* = 18) and 8WA1221 (n = 3) were submitted to J. Feathers at the University of Washington for single-grain OSL dating. See Supplementary text “Luminescence Dating Methods” for details of OSL dating.

### Granulometry

Methodologically, standard sieve analysis procedures were used (Folk^52^). Bulk samples were first dried then split and weighed. Dry sieving of each sample was conducted using standard USGS sieves (−1 through 4 phi) and consisted of sieving for 10 min before weighing each sieve fraction on a digital scale.

The traditional phi grade scale was used after Folk and Ward^53^:

$$ \Phi = - \log \;2d $$

where *d* = grain diameter in mm.

The GRADISTAT program (Blott and Pye^54^) was used to calculate sand-sized-fraction grain size statistical parameters. This program is integrated into an Excel spreadsheet database and allows rapid analysis of large numbers of samples for grain-size analysis using a variety of methods. As stated above, the standard logarithmic scale (phi scale) of Folk and Ward^53^ graphical methods was used in this analysis. These measures include graphic mean grain size, inclusive graphic standard deviation, inclusive graphic skewness, and graphic kurtosis (Folk^52^; Folk and Ward^53^). The sand fraction and total fines (silt + clay) percentages are also calculated for each sample.

### Microspherule analysis

For analysis of sediment samples for magnetic microspherules, samples are weighed and then ultrasonicated for 30 min with a chemical dispersant (sodium metaphosphate). Following ultrasonication, samples are wet-sieved over a 20-micron sieve and dried under a heat lamp. Processed samples are then size sorted and sediment fractions between < 125 and ≥ 63 microns are spread thinly on a large sheet of paper. A neodymium magnet is placed inside a plastic bag and slowly moved over the size-sorted sediment fractions to extract the magnetic grains if present. This process is repeated three times to extract the majority of magnetic grains. These grains are then examined under a stereo binocular microscope to determine if microspherules are present. When present, microspherule candidates are counted and a sample is placed on carbon SEM tape for analysis by scanning electron microscopy (SEM) and energy-dispersive x-ray spectroscopy (EDS) analysis. Only SEM and EDS analysis is able to confirm the identification of dendritic microspherules (LeCompte et al*.*^15^). The spherule counts and total sediment sample weight are used to estimate spherules/Kg for each sample.

### Supplementary Information

Supplementary Information.

## Data Availability

All data generated or analyzed during this study are included in this published article [and its supplementary information files].

## References

[1] Jones, C. B. *Wakulla Springs Lodge West (formerly WA418, now part of WA329)*. Florida Bureau of Archaeological Research Site File Document 5152 (1971).

[2] Jones, C. B. and Tesar, L. D. *Wakulla Springs Lodge site (8Wa329) in Edward Ball Wakulla Springs State Park Wakulla County, Florida*. Florida Master Site File Report 6602. Florida Bureau of Archaeological Research, Tallahassee, FL (2004).

[3] Rasmussen SO (2014). A stratigraphic framework for abrupt climatic changes during the Last Glacial period based on three synchronized Greenland ice-core records: refining and extending the INTIMATE event stratigraphy. Quatern. Sci. Rev..

[4] Petaev M (2013). Large Pt anomaly in the Greenland ice core points to a cataclysm at the onset of Younger Dryas. Proc. Natl. Acad. Sci. USA.

[5] Moore CR (2017). Widespread platinum anomaly documented at the Younger Dryas onset in North American sedimentary sequences. Scientific Reports.

[6] Moore CR, Brooks MJ, Goodyear AC (2019). Sediment Cores from White Pond, South Carolina, contain a Platinum Anomaly, Pyrogenic Carbon Peak, and Coprophilous Spore Decline at 12.8 ka. Sci Rep.

[7] Moore AMT (2023). Abu Hureyra, Syria, Part 1: Shock-fractured quartz grains support 12,800-year-old cosmic airburst at the Younger Dryas onset. ScienceOpen.

[8] Moore AMT (2023). Abu Hureyra, Syria, Part 2: Additional evidence supporting the catastrophic destruction of this prehistoric village by a cosmic airburst ∼12,800 years ago. ScienceOpen.

[9] Moore, A. M. T. *et al.* Abu Hureyra, Syria, Part 2: Additional evidence supporting the catastrophic destruction of this prehistoric village by a cosmic airburst ∼12,800 years ago, SUPPLEMENTARY DATA. https://zenodo.org/record/8284724 (ed Zenodo) (Zenodo, Zenodo, 2023).

[10] Moore AMT (2023). Abu Hureyra, Syria, Part 3: Comet airbursts triggered major climate change 12,800 years ago that initiated the transition to agriculture. ScienceOpen.

[11] Firestone RB (2007). Evidence for an extraterrestrial impact 12,900 years ago that contributed to the megafaunal extinctions and the Younger Dryas cooling. Proc. Natl. Acad. Sci. USA.

[12] Bunch TE (2012). Very high-temperature impact melt products as evidence for cosmic airbursts and impacts 12,900 years ago. Proc. Natl. Acad. Sci. USA.

[13] Israde-Alcantara I (2012). Evidence from central Mexico supporting the Younger Dryas extraterrestrial impact hypothesis. Proc. Natl. Acad. Sci. USA.

[14] Wittke JH (2013). Evidence for deposition of 10 million tonnes of impact spherules across four continents 12,800 y ago. Proc. Natl. Acad. Sci. USA.

[15] LeCompte MA (2012). Independent evaluation of conflicting microspherule results from different investigations of the Younger Dryas impact. Proc. Natl. Acad. Sci. USA.

[16] Pino M, Abarzúa AM, Astorga G (2019). Sedimentary record from Patagonia, southern Chile supports cosmic-impact triggering of biomass burning, climate change, and megafaunal extinctions at 12.8 ka. Sci. Rep..

[17] Andronikov AV (2015). Geochemical evidence of the presence of volcanic and meteoritic materials in Late Pleistocene lake sediments of Lithuania. Q. Int..

[18] Andronikov AV (2016). Implications from chemical, structural and mineralogical studies of magnetic microspherules from around the lower Younger Dryas boundary (New Mexico, USA). Geogr. Ann. A.

[19] Moore AMT, Kennett JP, Napier WM (2020). Evidence of Cosmic Impact at Abu Hureyra, Syria at the Younger Dryas Onset (~128 ka): High-temperature melting at >2200 °C. Sci. Rep..

[20] Wolbach WS (2018). Extraordinary biomass-burning episode and impact winter triggered by the Younger Dryas cosmic impact <12800 years ago. 1. Ice cores and glaciers. J Geol.

[21] Wolbach WS (2018). Extraordinary biomass-burning episode and impact winter triggered by the Younger Dryas cosmic impact 12,800 years ago. 2. Lake, marine, and terrestrial sediments. J. Geol..

[22] Neill WT (1964). The Association of Suwannee points and extinct animals in Florida. Florida Anthropologist.

[23] Dunbar, J. S. Resource Orientation of Clovis and Suwannee Age Paleoindian Sites in Florida. *In Clovis Origins and Adaptions*, edited by R. Bonnichsen and K. Turnmire, pp. 185–213. Center for the Study of the First Americans, Corvallis, Or. (1991).

[24] Dunbar JS (2016). Artifacts and Technology, Chapter 6.

[25] Dunbar, J. S. The Effect of Geohydrology and Natural Resource Availability on Site Utilization at the Fowler Bridge Mastodon Site (8Hi383c/uw) in Hillsborough County, Florida. In Report of Phase II Underwater Archaeological Testing at the Fowler Bridge Mastodon Site (8Hi393c/uw) Hillsborough County, Florida., pp. 63–106. The Department of State, Tallahassee (1981).

[26] Dunbar, J. S. Pleistocene-Holocene Climate Change: Chronostratigraphy and Geoclimate of the Southeast United States, Chapter 5. In *First Floridians and Last Mastodons: the Page-Ladson Site on the Aucilla River*, edited by S. D. Webb, pp. 103–158. Springer Press (2006).

[27] Thulman DK (2009). Freshwater availability as the constraining factor in the middle paleoindian occupation of North-Central Florida. Geoarchaeology.

[28] Webb DS (1974). Pleistocene Mammals of Florida.

[29] Milanich JT (1994). Archaeology of Precolombian Florida.

[30] Halligan JJ (2016). Pre-Clovis occupation 14,550 years ago at the Page-Ladson site, Florida, and the peopling of the Americas. Science Advances.

[31] Galbraith RF, Roberts RG (2012). Statistical aspects of equivalent dose and error calculation and display in OSL dating: An overview and some recommendations. Quatern. Geochronol..

[32] Chauhan, N., Selvam, *et al.* Distribution of natural beta dose to individual grains in sediments. *Proceedings of the Indian National Science Academy* (2021).

[33] Feathers, J. K., Evans, M., Stratford, D. J., and de la Peña, P. Exploring complexity in luminescence dating of quartz and feldspars at the Middle Stone Age site of Mwulu’s Cave (Limpopo, South Africa). *Quaternary Geochronology* (2020).

[34] Moore CR, Goodyear AC, Moore CR (2018). Regional Manifestations of Late Quaternary Climate Change and Archaeological Site Burial along the South Atlantic Coastal Plain. Early Human Life on the Southeastern Coastal Plain.

[35] Ramsey CB (2009). Bayesian Analysis of Radiocarbon Dates. Radiocarbon.

[36] Reimer PJ (2020). The IntCal20 Northern Hemisphere Radiocarbon Age Calibration Curve (0–55 cal kBP). Radiocarbon.

[37] Kennett, J. P. *et al.* Bayesian chronological analyses consistent with synchronous age of 12,835–12,735 Cal B.P. for Younger Dryas boundary on four continents. *Proc Nat Acad Sci* **112**, E4344–4353 (2015). 10.1073/pnas.1507146112 PMC4538614 26216981

[38] Bunch TE (2021). A Tunguska sized airburst destroyed Tall el-Hammam a Middle Bronze Age city in the Jordan Valley near the Dead Sea. Sci. Rep..

[39] Brooks, Mark J., and Sassaman, Kenneth E. Point Bar Geoarchaeology in the Upper Coastal Plain of the Savannah River Valley, South Carolina: A Case Study. In *Archaeological Geology of North America*, edited by N. P. Lasca and J. E. Donahue, pp. 183–197. Geological Society of America, Centennial Special Volume 4. Boulder, Colorado (1990).

[40] Thackeray FJ, Scott L, Pieterse P (2019). The Younger Dryas interval at Wonderkrater (South Africa) in the context of a platinum anomaly. Palaeontol Afr.

[41] Dunbar, J. S. *et al. The Wakulla Springs Mysterious Waters: a report of archaeological investigations*. DHR Grants Office by the Aucilla Research Institute for DHR Grant18.h.sc.300.041. Florida Master Site File manuscript 26243, Tallahassee, Florida (2019).

[42] Hemmings, A. C. and Dunbar J. S. Chapter 17 Data Regarding North Florida Paleoclimate of the Allerød and Early Younger Dryas from Wakulla Springs Underwater Site 8WA24a pages 386–394 in *The Wakulla Springs Mysterious Waters: a report of archaeological investigations*. DHR Grants Office by the Aucilla Research Institute for DHR Grant 18.h.sc.300.041. Florida Master Site File manuscript 26243, Tallahassee, Florida (2019).

[43] Uliasz-Bocheńczyk, A. & Mokrzycki, E. The elemental composition of biomass ashes as a preliminary assessment of the recovery potential. *gospodarka surowcami mineralnymi* **34**, 115–132 (2018).

[44] Wierońska, F., Makowska, D., Strugała, A. & Bytnar, K. Analysis of the content of nickel, chromium, lead and zinc in solid products of coal combustion (CCPs) coming from Polish power plants in *IOP Conference Series: Earth and Environmental Science.* 012029 (IOP Publishing).

[45] Tirez, K. *et al.* A multi-element determination of critical elements (REE, PGM,…) in solid (waste) materials by SFICP-MS and EDXRF in *Proceeding of second symposium on urban mining.* 19–21.

[46] Thy P, Segobye AK, Ming DW (1995). Implications of prehistoric glassy biomass slag from east-central Botswana. J. Archaeol. Sci..

[47] Bonté P, Jehanno C, Maurette M, Brownlee D (1987). Platinum metals and microstructure in magnetic deep sea cosmic spherules. J. Geophys. Res.: Solid Earth.

[48] Rudraswami, N., Parashar, K. & Shyam Prasad, M. Micrometer‐and nanometer‐sized platinum group nuggets in micrometeorites from deep‐sea sediments of the Indian Ocean. *Meteorit. Planet. Sci.* **46**, 470–491 (2011).

[49] Rudraswami N (2014). Refractory metal nuggets in different types of cosmic spherules. Geochimica et Cosmochimica Acta.

[50] Genge MJ, Davies B, Suttle MD, van Ginneken M, Tomkins AG (2017). The mineralogy and petrology of I-type cosmic spherules: Implications for their sources, origins and identification in sedimentary rocks. Geochim Cosmochim Acta.

[51] Hoffman, E. L. & Dunn, B. Sample preparation and bulk analytical methods for PGE. CIM Special Volume 54: The Geology, Geochemistry and Mineral Beneficiation of Platinum Group Elements. Edited by Louis J. Cabri, 1–11 (2002).

[52] Folk RL (1974). Petrology of Sedimentary Rocks.

[53] Folk RL, Ward WC (1957). Brazos River Bar: A Study in the Significance of Grain Size Parameters. J. Sediment. Petrol..

[54] Blott SJ, Pye K (2001). Grain size statistics program. Earth Surface Processes Landforms.

