# Supplementary material for: Platinum and microspherule peaks as chronostratigraphic markers for onset of the Younger Dryas at Wakulla Springs, Florida
Source: Sci Rep. 2023 Dec 20;13:22738. doi: 10.1038/s41598-023-50074-8 (PMC10733423; doi:10.1038/s41598-023-50074-8)
Supplement: Supplementary file 1 — Supplementary Information. [file 41598_2023_50074_MOESM1_ESM.pdf]

Supplementary Information: Platinum and Microspherule Peaks as Chronostratigraphic Markers for Onset of the Younger Dryas at Wakulla Springs, Florida

Christopher R. Moore,[1*] Mark J. Brooks,[1] James S. Dunbar,[2] C. Andrew Hemmings,[2] Kurt Langworthy,[3] Allen West,[4] Malcolm A. LeCompte,[5] Victor Adedeji,[6] James P. Kennett,[7] and James K. Feathers[8]

DRAFT: DO NOT CITE WITHOUT THE AUTHORS' PERMISSION

[1]South Carolina Institute of Archaeology and Anthropology, University of South Carolina, P.O. Box 400, New Ellenton, SC 29809, USA; [2]Aucilla Research Institute Inc., 555 North Jefferson Street, Monticello, FL 32344, USA; [3]CAMCOR, University of Oregon, 1443 E 13th Ave, Eugene, OR, 97403, USA; [4]Comet Research Group, Prescott, AZ USA; [5]Center of Excellence in Remote Sensing Education and Research, Elizabeth City State University, Elizabeth City, NC 27909; [6]Department of Natural Sciences, Elizabeth City State University, Elizabeth City, NC, 27909, USA; [7]Department of Earth Science and Marine Science Institute, University of California, Santa Barbara, CA 93106; [8]University of Washington, Luminescence Dating Laboratory, 125 Raitt Hall, Seattle, 98195-3412, WA, USA.

*To whom correspondence should be addressed. E-mail: MOORECR@mailbox.sc.edu

Regional Environments and Soil Distribution

Edward Ball Wakulla Springs State Park is located within one of the karst regions of northern Florida with varying topographic characteristics shown in Supplementary Figure 1. Within the areas of 8WA329 and 8WA1221 (excavated during 2017 and 2018) there is a range of soil types[1] (Supplementary Figure 2) which are described as follows:

Otela fine sand, 0-5 percent slope. Otela fine sands consist of moderately well-drained soils on nearly level to sloping low knolls, broad uplands, and side slopes adjacent to stream channels on the Coastal Plain. They formed in sandy and loamy marine or eolian sediment. Slopes range from 0 to 5 percent. A perched water table is above the subsoil during wet periods and at a depth of more than 72 inches for the remainder of the year. Soil reaction ranges from very strongly acidic to moderately alkaline. Texture ranges include loamy fine sand, fine sand, sand, sandy clay loam, sandy loam, and fine sandy loam.

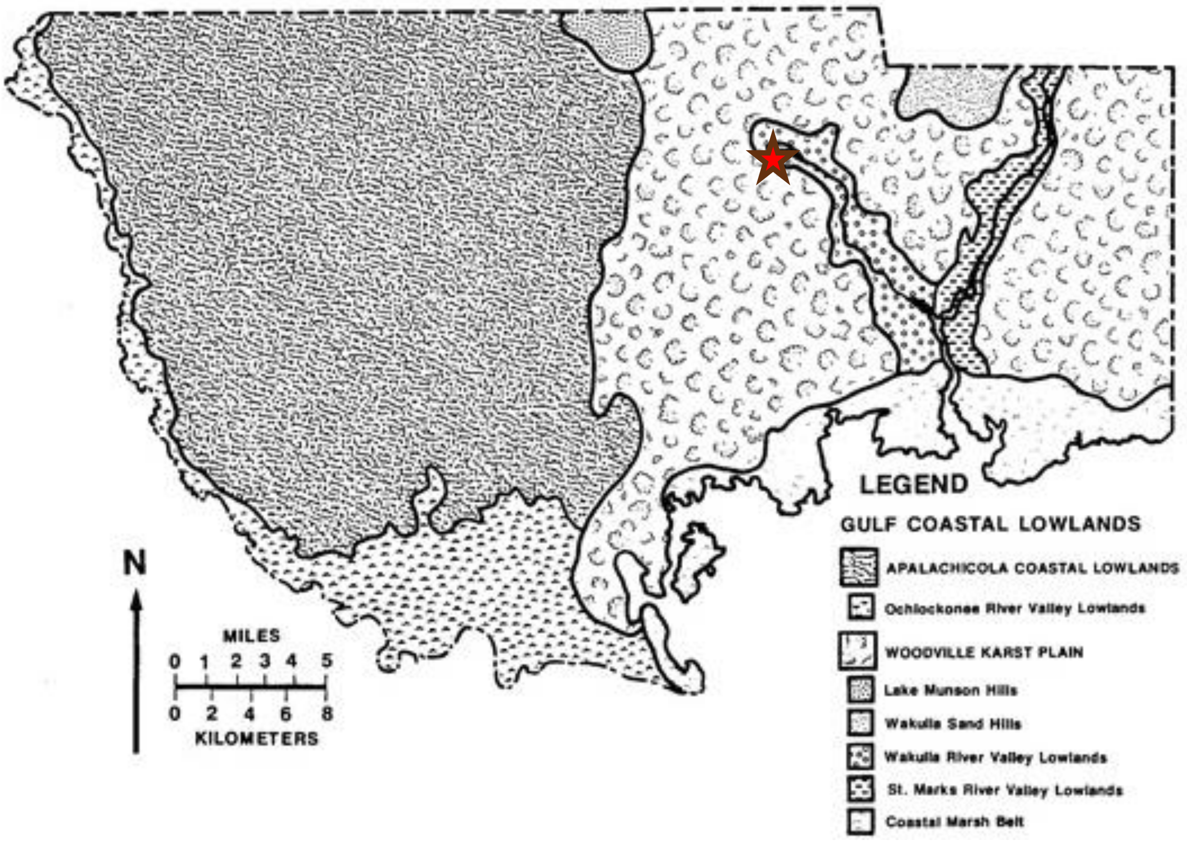

Supplementary Figure 1. Topographic Characteristics of Wakulla County area. The location of Edward Ball Wakulla Springs State Park is indicated by a red star. The map is public domain and adapted from USDA Soil Survey: 4, Soil Survey of Wakulla County, Florida (1991)[1]. (https://books.google.com/books?id=N28QxAEACAAJ&pg=PP1#v=twopage&q&f=false).

Ridgewood fine sand, 0-5 percent slope. Ridgewood fine sands consist of somewhat poorly drained, rapidly permeable soils on uplands. They formed in thick beds of sandy marine deposits. Slopes range from 0 to 5 percent. The water table is 24 to 40 inches for 2 to 4 months or more during most years, rising for brief periods with rain and decreasing below 40 inches when extremely dry. Soil reaction ranges from very strongly acidic to medium acid. The texture is sand or fine sand.

Tooles-Nutall fine sands. Tooles-Nutall fine sand is a complex that consists of very poorly to poorly drained, nearly level soils in flatwoods. The water table is high, within 0-20 inches/year, and in some cases, floods occur for up to 6 months of the year. Soil reaction is very strongly acidic to moderately alkaline. Textures include fine sand, sand, sandy, sandy clay loam, and sandy clay.

Moriah-Pilgrims fine sands. Moriah-Pilgrims fine sands are a complex consisting of somewhat poorly drained, slowly to moderately permeable, nearly level soils of low uplands and flatwoods. They formed in sandy and loamy marine sediments. Slopes range from 0 to 2 percent. The water table is within a depth of 18 to 36 inches for 2 to 5 months in most years. Soil reaction ranges from extremely acidic to moderately alkaline with increasing depth. Textures include sand, fine sand, sandy clay loam, sandy clay, and sandy loam.

As noted, within the areas excavated in spring 2017, Ridgewood fine sand, Tooles-Nutall fine sands, Moriah-Pilgrims fine sand, and Otela limestone substratum-Ortega fine sands are the dominant soil types. Vegetation in the southern grid area (see Supplementary Figure 2) is predominantly hardwoods and cypress in the areas nearest the river and pine flatwoods further south. In the northern grid area, upland hardwoods predominate, including hickory and oak.

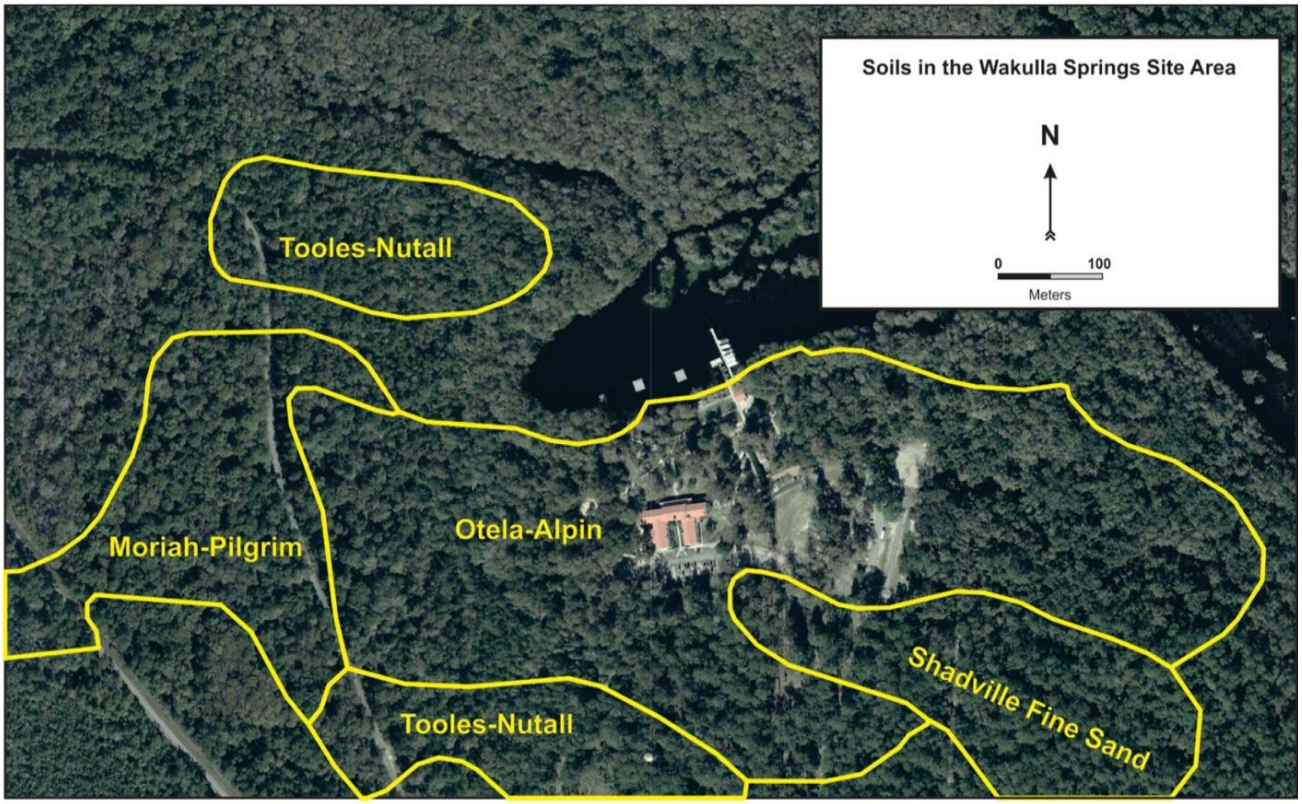

Supplementary Figure 2. Map of Soil Distributions, Wakulla Springs State Park. The map was produced in Global Mapper (v.19.1), https://www.bluemarblegeo.com/global-mapper/ using an aerial image from the Florida Department of Transportation (FDOT) (https://fdotewp1.dot.state.fl.us/AerialPhotoLookUpSystem/) and soil layers from the USDA Soils Survey:4 (https://websoilsurvey.nrcs.usda.gov/app/)[1].

Supplemental Granulometry

KU 4 Granulometry. A single sediment column was collected in continuous 2.5-cm intervals from the KU4 profile in the Kennard excavation block (see Figure 2 in the main paper). An unusual characteristic of the sediments from 8WA329 is the bimodal size distribution of the sediments with low abundances of very coarse and coarse sand in each 2.5-cm column sample. However, we predicted on geomorphic grounds that deposition would have been dominated by eolian processes, which is consistent with observation. As indicated by the mean grain size and cumulative sand fraction data (Supplementary Figures 3-5), the coarsest material is at the base of the column from 120 to 137.5 cmbs and the top of the column from 0 to 45 cmbs. While speculative, the coarser material at the base may have been inherited from the weathering limestone surface immediately below, and the material from higher up in the column may have been inherited through slopewash from similarly derived material upslope. More on this is below in the discussion of PU1 results.

In KU4, the sediments are moderately sorted and mostly positively skewed. This is consistent, or at least not inconsistent, with eolian deposits, although most eolian deposits are somewhat better sorted. The coarsest samples tend to exhibit the best sorting, presumably due to the winnowing of finer sediments. Sediment fractions are dominated by medium and fine sand. From the base of the sediment column up to near 65 cmbs, the percentage of medium sand declines slightly, and the percentages of fine sand increase slightly, but still in lower frequencies than the medium sand. Above 65 cmbs, the medium sand increases in frequency, and the fine sand declines. Overall, the net effect is a slightly coarsening upward sequence, which is consistent with many eolian deposits (Ahlbrandt and Fryberger[2]:21, 26-27, 29-31, and 33; Davis[3]:447).

PU1 Granulometry. PU1 is located about 50 m upslope from KU4 to the south (Figure 2 in the main paper). Like KU4, PU1 is dominated by primary eolian depositional processes with localized, small-scale (vertically and laterally) eolian and slopewash reworking of surface sediments. Unlike KU4, PU1's upcolumn sedimentary parameters (statistical and fractionation) are less variable and much more consistent and predictable, hence more readily interpreted for landform evolution, site formation, and surface burial processes.

Sedimentological parameters considered include mean grain size, sorting, skewness, and kurtosis. (Supplementary Figure 6). Kurtosis measures the relationship between sorting in the central portion of the curve to the proportion in the tails (Folk[4]). The mean grain size coarsens upward overall and by individual depositional events; the clearer upcolumn cyclic variation provides greater confidence than with KU4 for interpreting the former locations of buried surfaces. Included in this interpretation is a scrutiny of the sediment fractionation data (below).

The PU1 sorting (standard deviation) upcolumn is consistently slightly better than KU4, and the PU1 skewness is consistently slightly negative, whereas KU4's skewness is slightly negative only in the middle of the column. With values <1.00, both PU1 and KU4 sediments are slightly platykurtic (flat curves) rather than leptokurtic (peaked distribution/curve with values >1.00.

In addition to often exhibiting a coarsening upward depositional trend, eolian deposits generally exhibit better sorting than shown for PU1 and KU4 sediments, as well as positive skewness (Ahlbrandt and Fryberger[1]:39; Davis[2]:185-186, 199, 427). The slight variation from the norm for eolian deposits may be attributed to the bimodal distribution of the sediments (Supplementary Figure 7), possibly indicating the addition of local, coarser-grained sediment. Greater sorting is contingent on not having too little or too much rigor (energy) and not having intense, short-duration processes (e.g., storms) that cause

much sediment movement too rapidly for sorting to occur. Similarly, the negative skewness of the PU1 and KU4 samples was produced by an abundance of coarse particles in the coarse end (tail) of the curves. Collectively, these data and the platykurtic distributions implicate a very local source(s) contributing a coarse component. In the immediate vicinity, surface and near-surface weathering of sandy limestone is the most likely contributor.

Percentage ranges among the sand fractions are slightly variable between PU1 and KU4, presumably due to variable sourcing and down-slope winnowing processes (Supplementary Figure 8). From the standpoint of eolian deposition, the dominant wind direction varies seasonally from southwesterly to northwesterly. So, given that the PU1 and KU4 locations are in a north-south direction rather than from west to east, there would likely be no direct transport of eolian sediments between these sites. On the other hand, since KU4 is located downslope from PU1, slopewash reworking of sediments was possible from KU4 to PU1.

PU1 samples are dominated by fine sand (31-40%), whereas the KU4 profile downslope to the north is dominated by medium sand (35-39%). KU4 samples generally have slightly more very coarse and coarse sand (9-14% vs. 8-10%), and PU1 and KU4 samples both contain very fine sand (16-25%).

PU23 and PU25 Granulometry. The PU22-23 and PU24-25 excavation units were dominated by eolian depositional processes, accompanied by localized, small-scale (vertical and lateral) eolian and slopewash reworking of surficial sediments. For these units, the primary goal of the granulometric analyses is to assist with the archaeostratigraphic correlation between the two nearby units (Figures 7 and 8 in the main paper). This correlation is complicated because of significant disturbance depths by modern leveling and filling. Consequently, the original ground surface elevation is unknown, making correlation more difficult, and hence, the granulometric, archaeological, chronometric, and geochemical data are especially important.

The PU23 and PU25 sediment columns were sampled in continuous 2.5-cm intervals from the present ground surface to the depth of excavation (190 cmbs for PU23 and 150 cmbs for PU25). All samples from the PU23 sediment column were processed, including those from the upper 80 cm that appear to have been disturbed by modern leveling and filling. Thus, there is a need to identify (Supplementary Figures 10-14) the disturbed-undisturbed boundary defined on sedimentological grounds. For the PU25, every third sample in the upper 80 cm was processed. However, the following presentation of data and their interpretation only considers samples below 80 cmbs for both PU23 and PU25.

The statistical parameters (mean grain size, sorting/standard deviation, skewness, kurtosis) for PU23 and 25 are presented in Supplementary Figures 10 and 13. In both sections, the mean grain size generally coarsens upwards in each depositional episode. The resulting clear upcolumn cyclic variations provide a solid base for identifying the formerly buried surfaces.

The samples in both columns are moderately sorted, with the coarsest samples exhibiting the greatest sorting, presumably due to the winnowing of finer sediments. The skewness of all samples is consistently slightly negative. With values <1.00, all samples are slightly platykurtic (flat curves) rather than leptokurtic (peaked distribution/curve with values >1.00).

The sediment fractionation data for PU23 and 25 are presented in Supplement Figures 11, 12, and 14. Only undisturbed sediments below 80 cmbs are considered. The sand fraction percentages vary slightly between PU23 and PU25 due to changes in sourcing and down-slope winnowing processes. In both sequences, medium and fine sands dominate, with 8.5-11.0% of very coarse-to-coarse sands and 15-25% of very fine sands. For PU23, large increases in the very coarse, coarse, and medium sand fractions at depths between 130-180 cmbs suggest underlying paleochannel sands or, more likely, increased coarser material derived from weathering of the limestones.

Both columns generally exhibit slight upward increases in very coarse, coarse, medium, and fine sands and a slight decrease in very fine sands. Thus, there is a slight upward coarsening, which is consistent with many, if not most, eolian depositional sequences.

Statement on Pedogenic Modifications and Illuviation of Spherules and Pt

Analysis of granulometric data for sediment profiles examined for microspherules and Pt (e.g., Supplementary Figures 3-14) reveal no relations between changes in sand or mud (silt+clay) fractions and Pt peaks and/or Fe-rich microspherule peaks (Supplementary Figure 12). Indeed, a distinct peak in the mud percentage occurs ~20 cm above the Pt and microspherules peak. Thus, while we recognize the ubiquitous presence of fly ash spherules in surface sediments from industrial pollution, there is no evidence of potential anthropogenic spherules or Pt being reworked downward through the soil and concentrated at lithologic breaks reflected by the detailed granulometry (Supplementary Figure 12). Moore *et al.*[4] similarly showed no correlation between pedogenic concentrations of mud and Pt anomalies from multiple sites across North America with well-dated YDB Pt anomalies (see Supplementary Information; Figure 16 in Moore *et al.*[5]). In addition, we show that spherules associated with Pt anomalies at Wakulla Springs have a very different chemistry than fly ash spherules (see main paper).

Soil horizons examined in this study include typical Coastal Plain sequences of Ap, E, and Bw soil horizons over weathered limestone. Surficial sands are leached medium to fine sands reworked by both aeolian and slope-wash processes. For PU23 and PU25, historic fill in the upper ~80 cm of sediment was obvious from the presence of mottled soils and historic artifacts. The contact between disturbed historic fill and underlying undisturbed sediments is abrupt. Very thin clay skins were observed on sand grains with slightly elevated silt and clay seen in the middle of the excavation profiles, representing a very weakly developed Bw horizon. Ages from luminescence dating are consistent with ages and depths for Paleoamerican artifacts reported for sandy Coastal Plain sites across the Southeastern United States (Moore *et al.*[6]). See Dunbar *et al.*[7,8] for discussions of local geology, soils, and prehistoric and historic contexts.

Luminescence Dating Methods

Luminescence dating is based on the accumulation of natural background ionizing radiation (i.e., absorbed dose) through time. The equivalent dose (D_e) is measured in the lab by artificially stimulating the acquired luminescence signal and calibrating that signal against a known dose. The measured equivalent dose is related to the time of burial by the equation:

$$\text{Age}(t) = D_e\ (\text{Gy})/D_r(\text{Gy}/t)$$

where D_e represents laboratory demined equivalent dose in greys (Gy) {unit representing absorbed dose in J/kg}, D_r represents average dose rate in greys, and t represents a unit of time. Equivalent dose (D_e), which is a measure of the total absorbed dose through time, is determined using the single-aliquot regenerative dose (SAR) protocol (Feathers[9]. Murray and Wintle[10]; Wintle and Murray[11]).

In the lab, luminescence samples were taken under red light conditions by first removing the potentially exposed sediment at the end of the collection tube. The remaining unexposed material is separated into size fractions by sieving. If the material contains abundant silt or clay, the sample is wet-sieved through a 90-µm screen. Otherwise, it is sieved dry. The greater than 90-µm fraction is treated with HCl and H_2O_2, rinsed three times with water, and dried. It is then dry sieved to retrieve the 180- to 212-µm fraction. This fraction is etched for 40 minutes in HF and then rinsed with water, HCl, and water again. After drying, it is passed through the 180-µm screen to remove any degraded feldspar. The material caught in the screen is density-separated using a lithium metatungstate solution of 2.67 specific gravity.

A combined total of 10908 grains were measured for all seventeen samples, but using a set of criteria for evaluating the signals from each grain, only 1721 grains were accepted for analysis (a 17.0%

acceptance rate). The distribution of D_e values was evaluated by the central age and finite mixture models (Galbraith and Roberts[12]). The finite mixture model sorts the distribution into single-aged components illustrated by radial graphs in Appendix B. The dose rate was estimated by alpha counting, beta counting, and flame photometry, taken on bulk samples. Some issues with dose rate for particular samples remain to be resolved (see discussion in Appendix B). Moisture content was taken as 10% ±5% and was used for all samples in dose rate adjustments. Over-dispersion values are very high for all samples, likely because of the significant post-depositional movement of sand grains. OSL ages are reported in years before AD 2018 to 1-sigma standard deviation.

Supplemental Figures

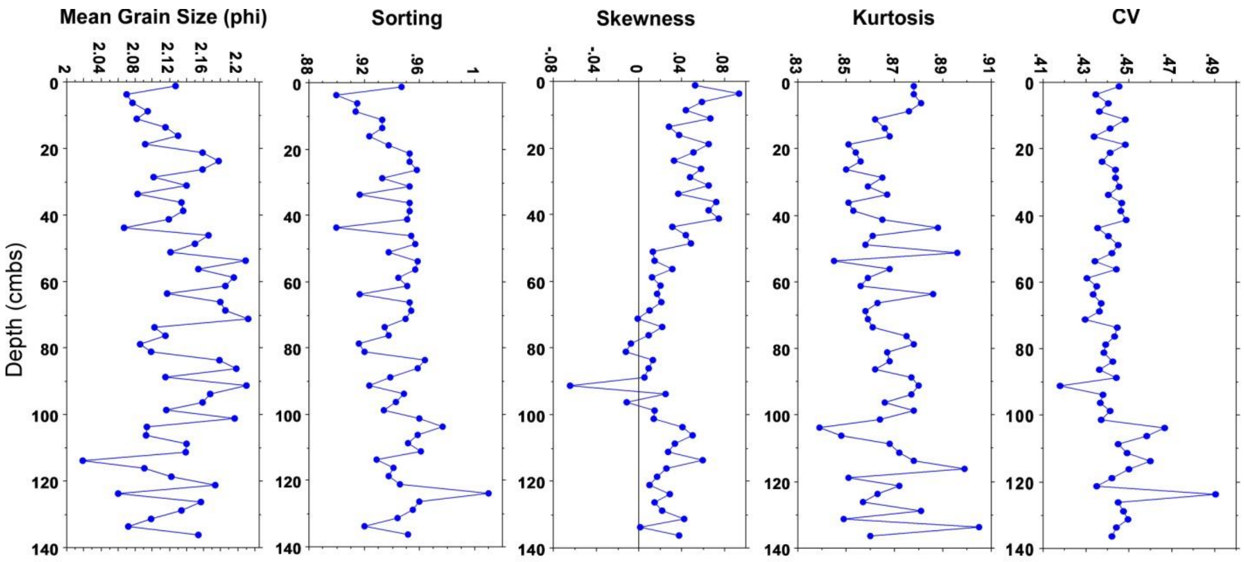

Supplementary Figure 3. Sand fraction statistical parameters for KU4. CV = Coefficient of Variation (calculated as Standard Deviation [sorting] as a percent of the mean).

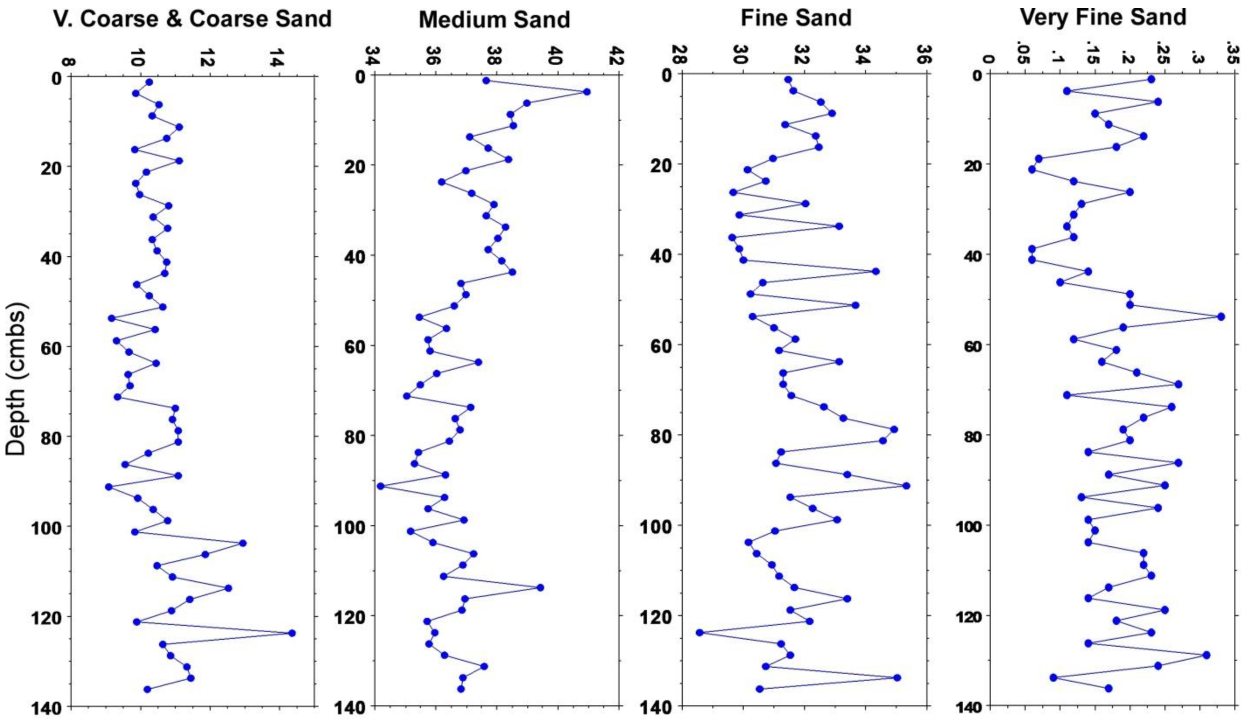

Supplementary Figure 4. Sand fraction data for KU4.

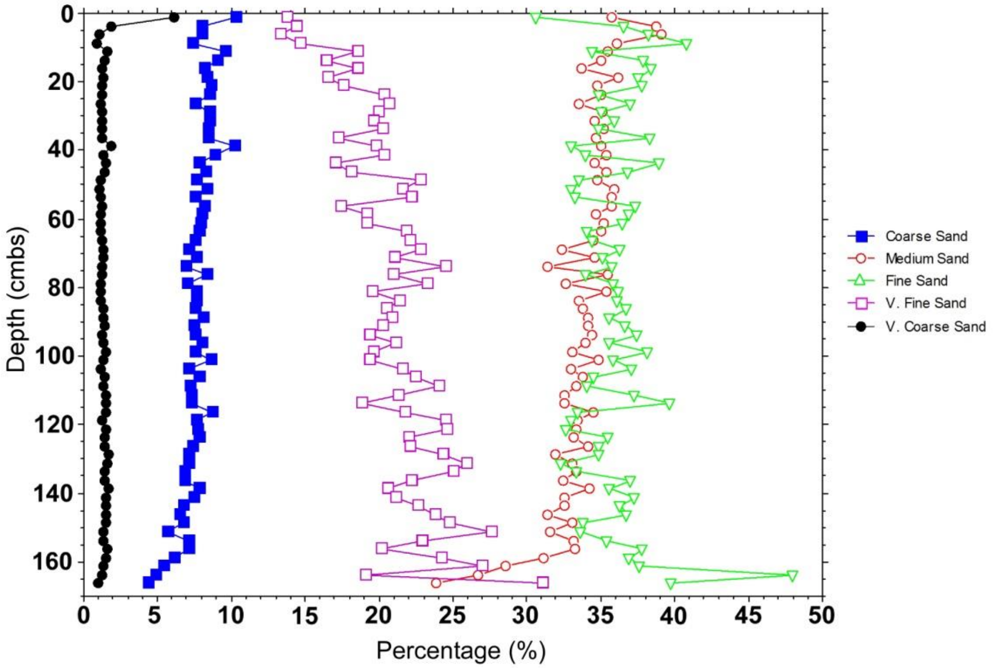

Supplementary Figure 5. Cumulative sand fraction data for KU4.

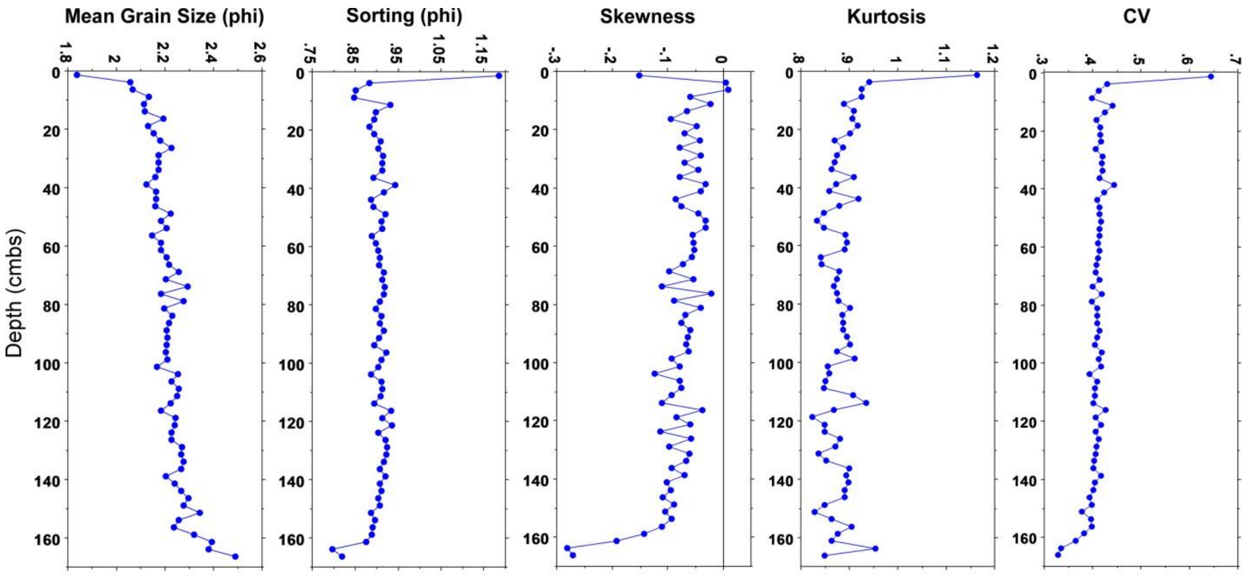

Supplementary Figure 6. Sand fraction statistical parameters for PU1. CV = Coefficient of Variation (calculated as Standard Deviation [sorting] as a percent of the mean).

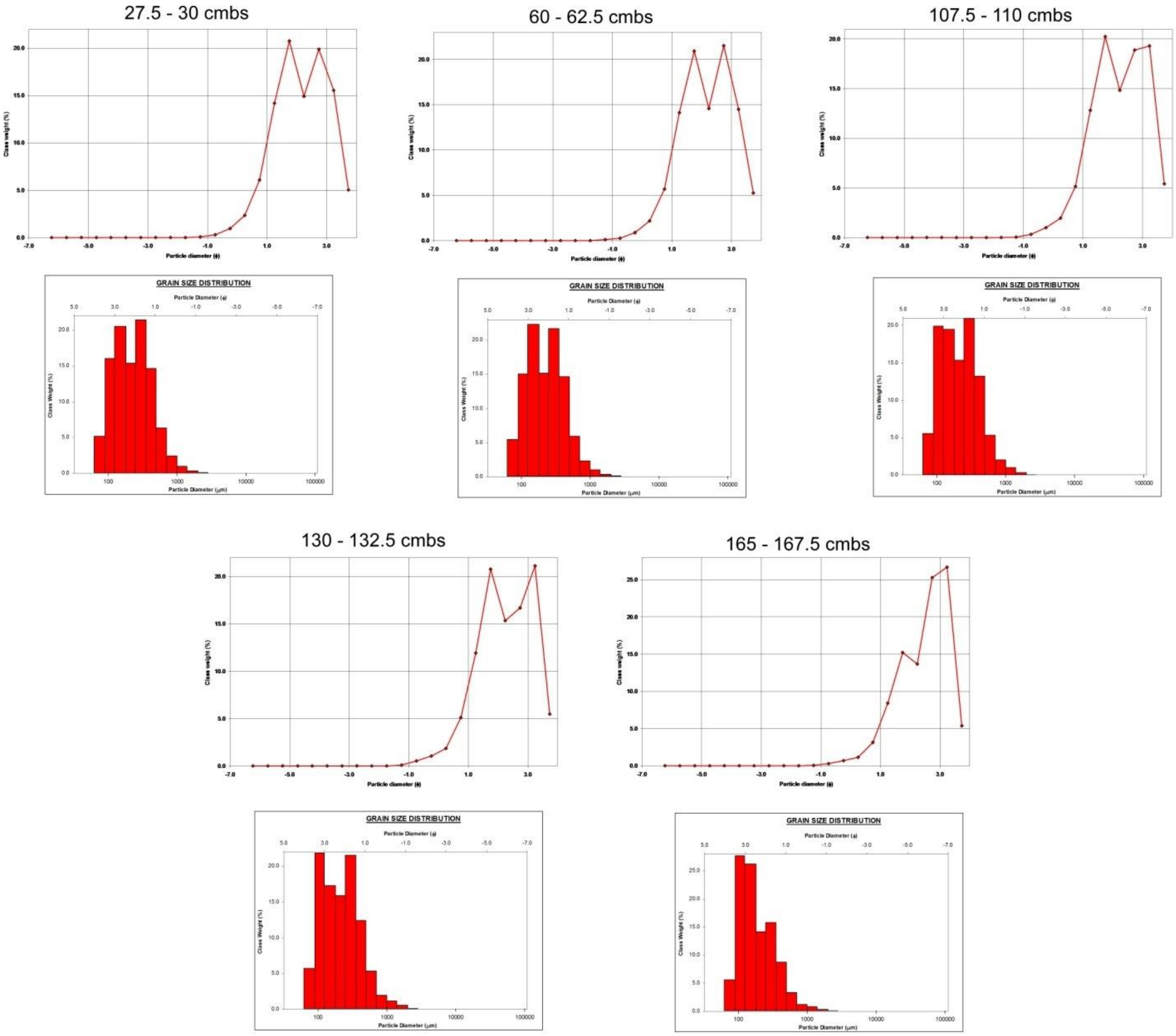

Supplementary Figure 7. Histograms show the bimodal character of sediment samples from PU1, which is typical for all sediments at 8WA329.

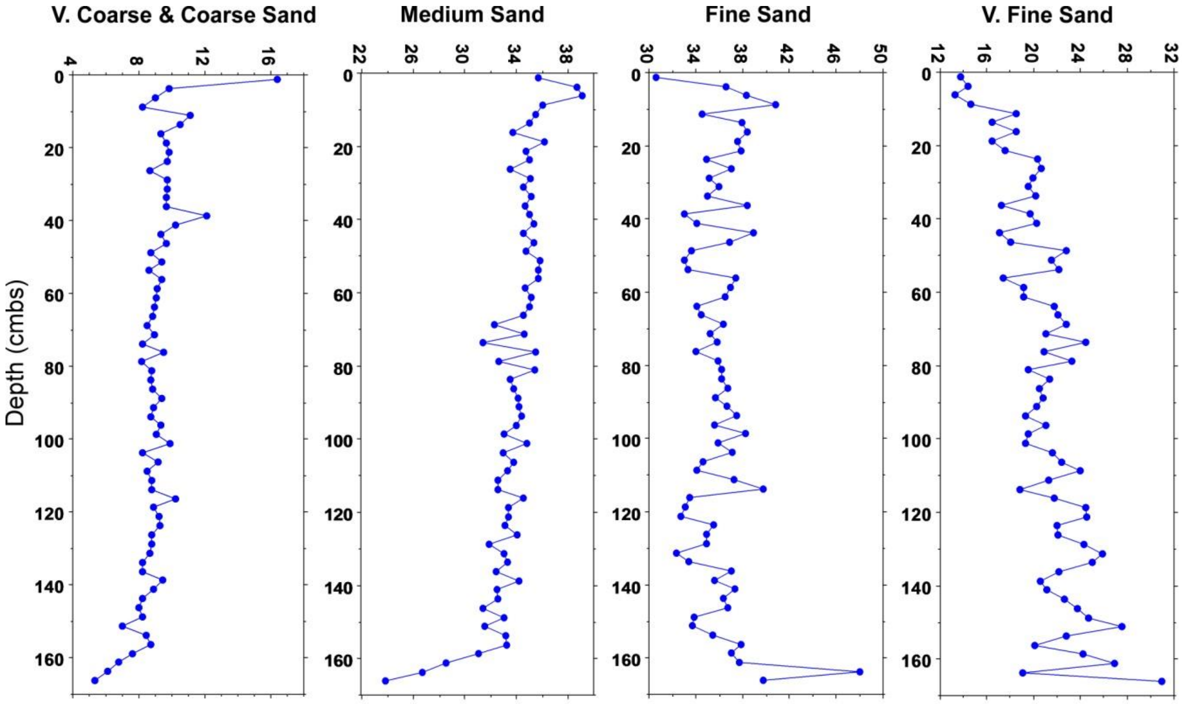

Supplementary Figure 8. Sand fraction data for PU1.

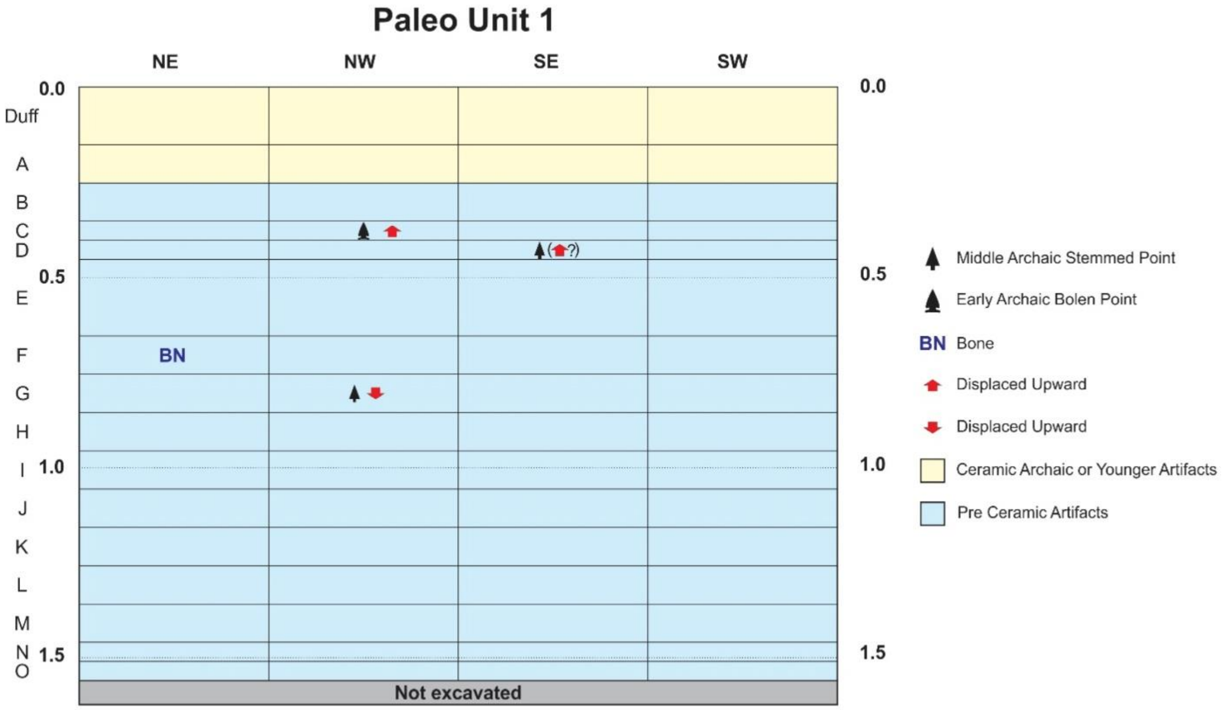

Supplementary Figure 9. Diagnostic artifacts for Paleo Unit 1 (PU1) showing vertical displacement direction of temporally-diagnostic artifacts.

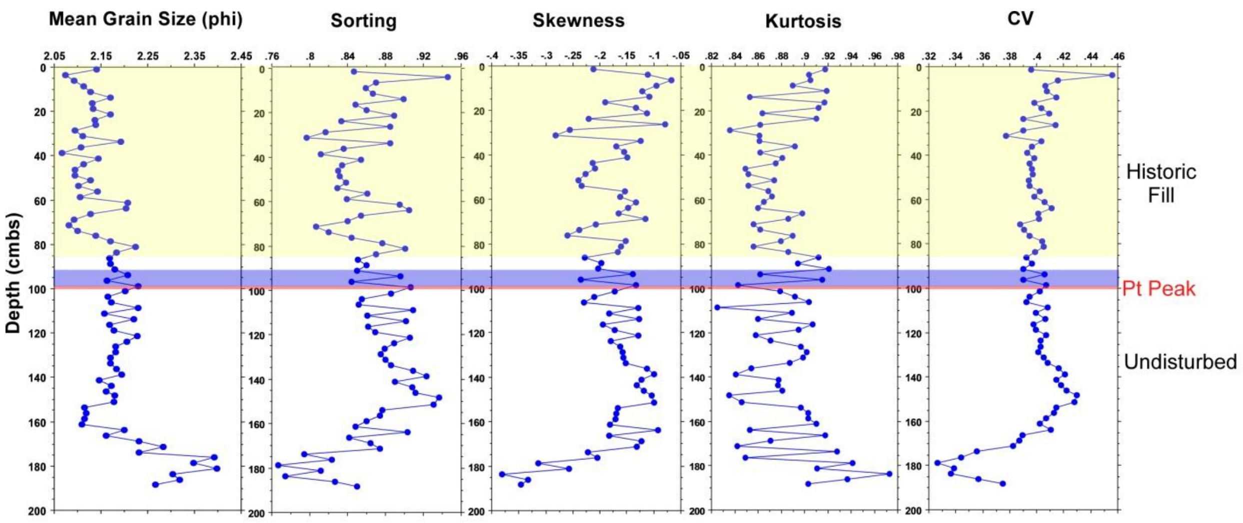

Supplementary Figure 10. PU23 sand fraction statistical parameters, Pt peak (red line), and spherule peak zone (light blue bar). Historic lodge fill is shown in light yellow.

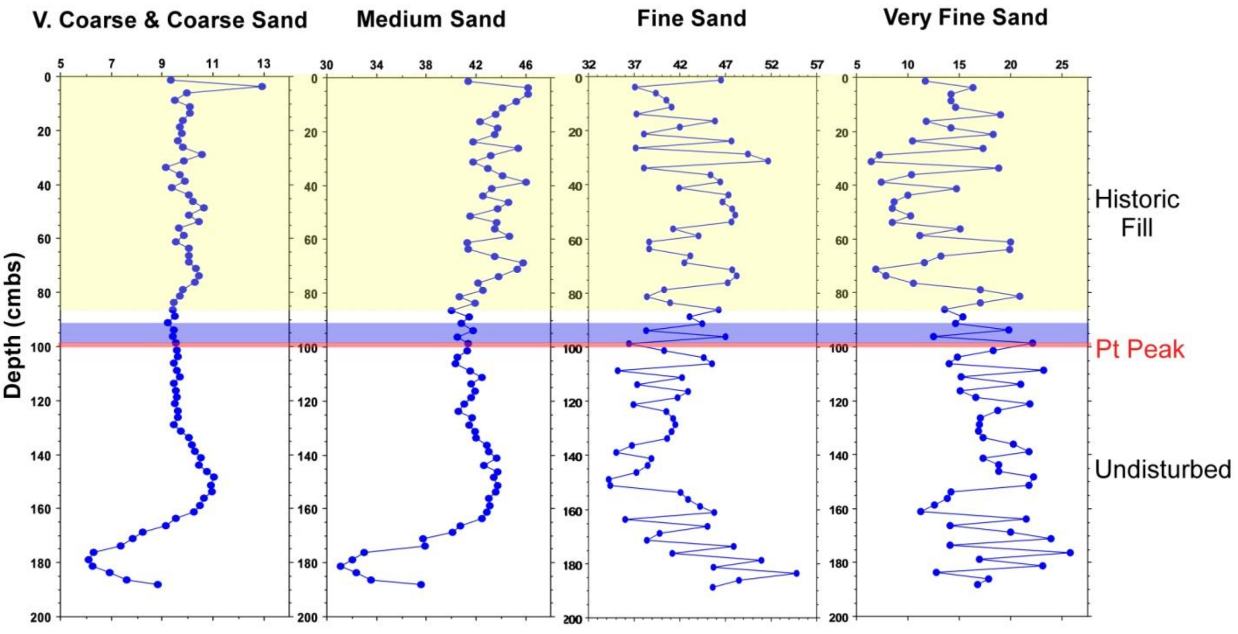

Supplementary Figure 11. PU 23 sand fraction data, Pt peak (red line), and spherule peak zone (light blue bar). Historic lodge fill is shown in light yellow.

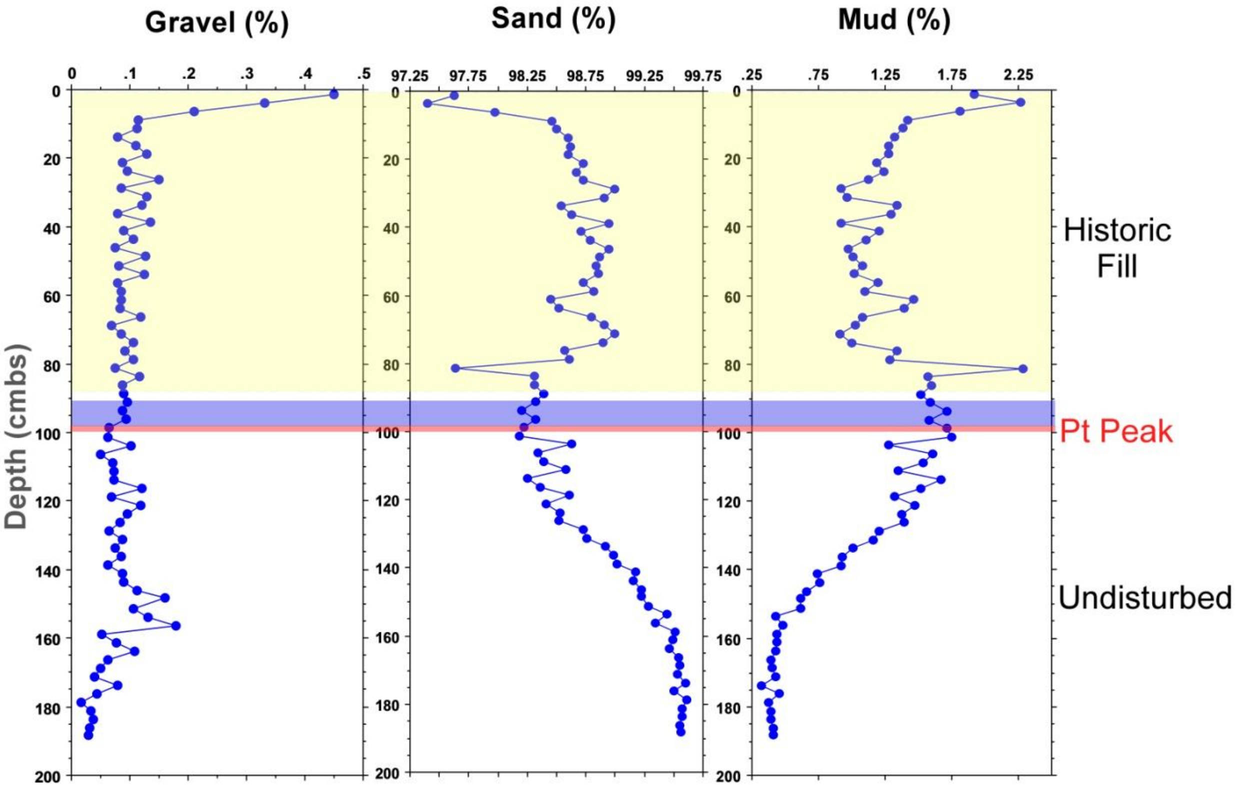

Supplementary Figure 12. PU 23 gravel, sand, and mud percentages, Pt anomaly (red line), and spherule peak zone (light blue bar). Historic lodge fill is shown in light yellow.

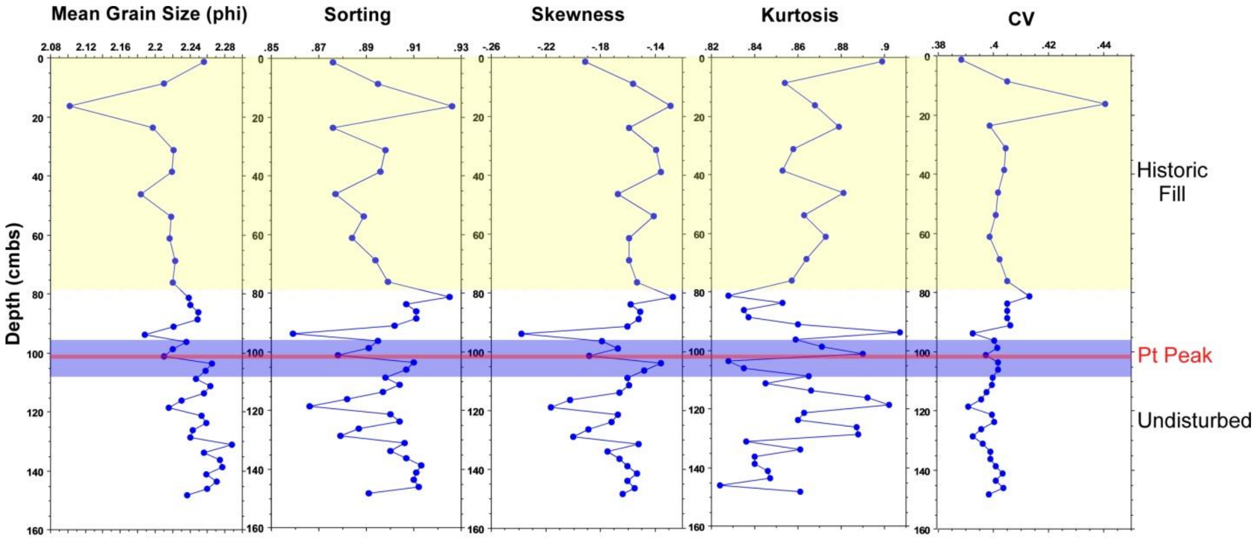

Supplementary Figure 13. PU25 sand fraction statistical parameters, Pt peak, and spherule peak zone (light blue bar). Historic lodge fill is shown in light yellow.

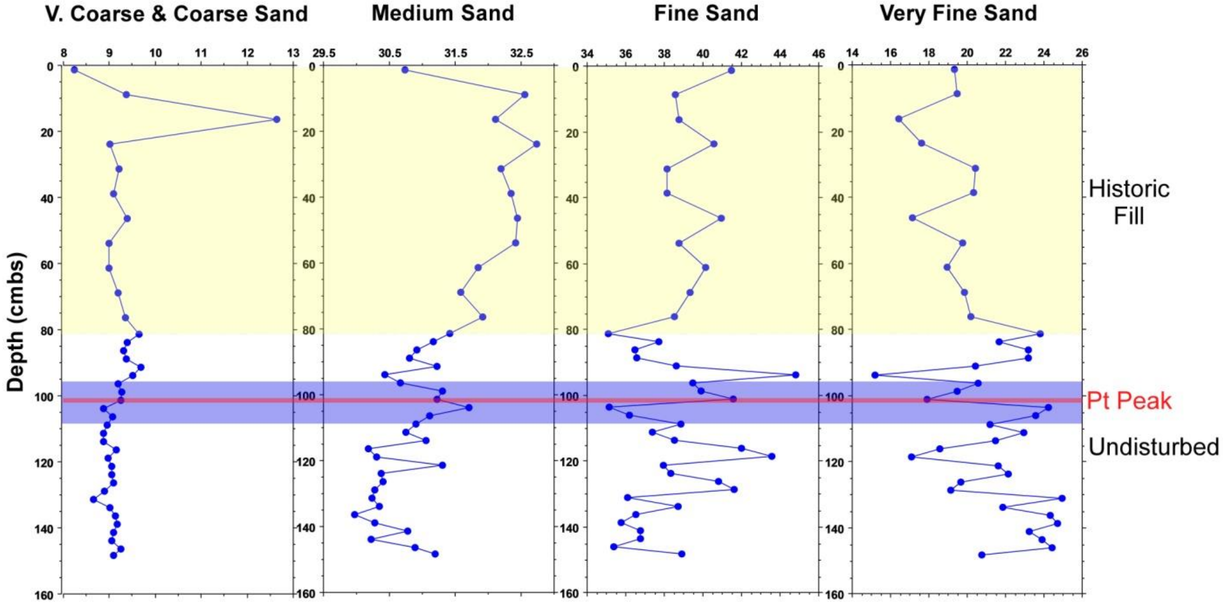

Supplementary Figure 14. PU 25 sand fraction data, Pt peak (red line), and spherule peak zone (light blue bar). Historic lodge fill is shown in light yellow.

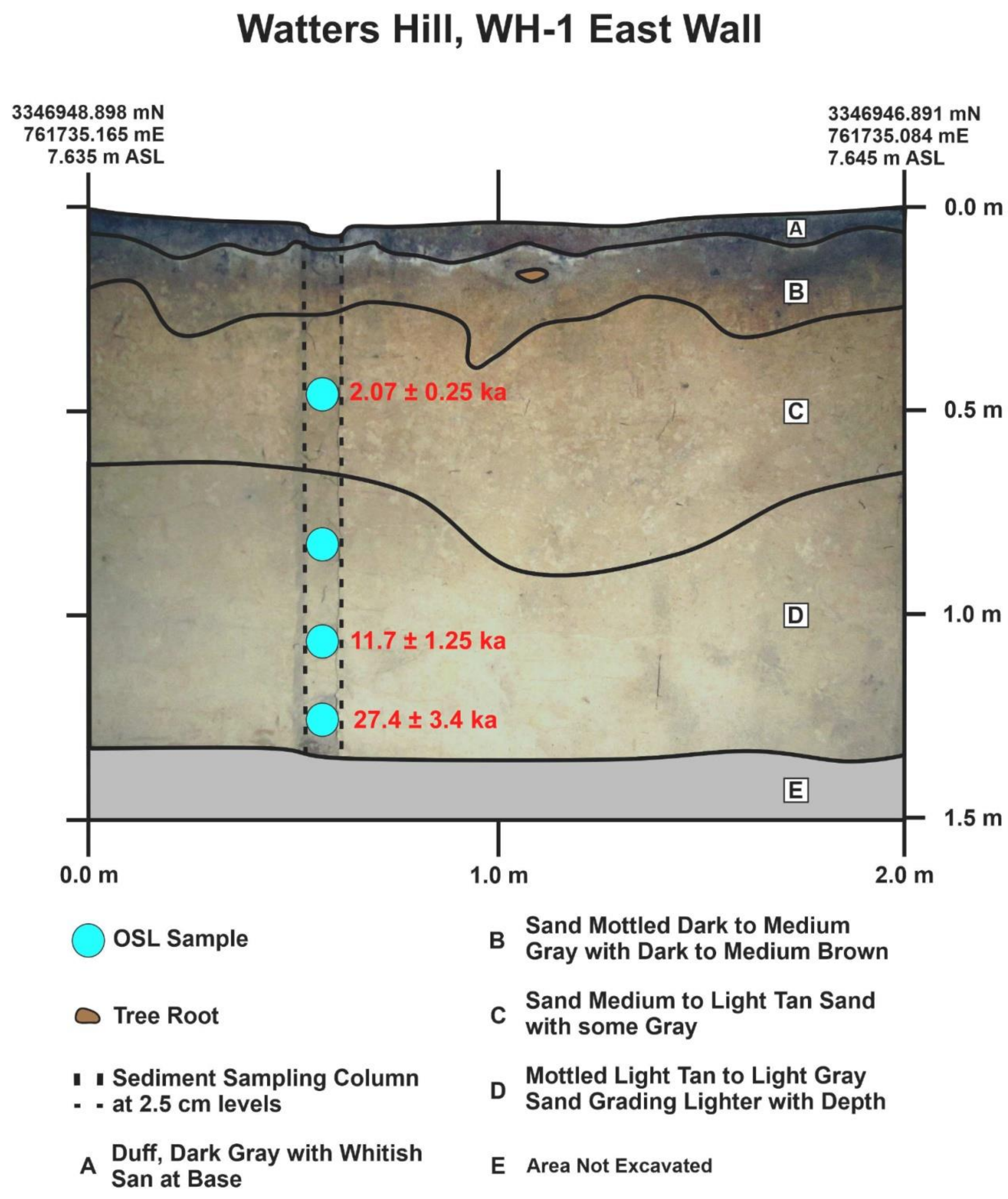

Supplementary Figure 15. Watters Hill 1 (WH1) profile at site 8WA1221 showing OSL age estimates (Table 1 in main paper).

Microspherule EDS Data

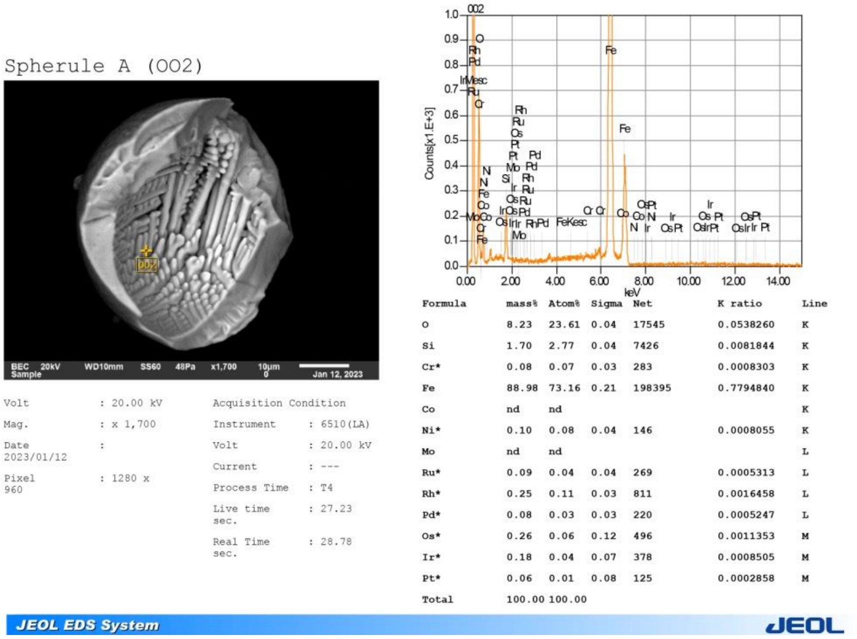

Formula	mass%	Atom%	Sigma	Net	K ratio	Line
O	8.23	23.61	0.04	17545	0.0538260	K
Si	1.70	2.77	0.04	7426	0.0081844	K
Cr*	0.08	0.07	0.03	283	0.0008303	K
Fe	88.98	73.16	0.21	198395	0.7794840	K
Co	nd	nd				K
Ni*	0.10	0.08	0.04	146	0.0008055	K
Mo	nd	nd				L
Ru*	0.09	0.04	0.04	269	0.0005313	L
Rh*	0.25	0.11	0.03	811	0.0016458	L
Pd*	0.08	0.03	0.03	220	0.0005247	L
Os*	0.26	0.06	0.12	496	0.0011353	M
Ir*	0.18	0.04	0.07	378	0.0008505	M
Pt*	0.06	0.01	0.08	125	0.0002858	M
Total	100.00	100.00				

Supplementary Figure 16. Scanning electron microscopy (SEM) and energy-dispersive x-ray spectroscopy (EDS) for spherule A from PU25 showing major and platinum-group elements (PGEs). Note oxygen depletion for spot EDS (002) inside of spherule.

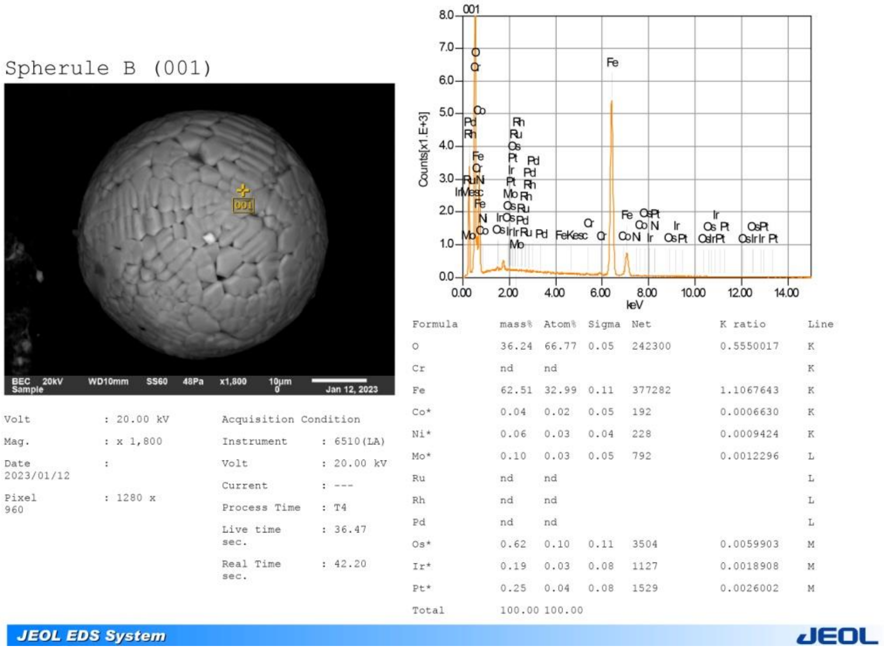

Formula	mass%	Atom%	Sigma	Net	K ratio	Line
O	36.24	66.77	0.05	242300	0.5550017	K
Cr	nd	nd				K
Fe	62.51	32.99	0.11	377282	1.1067643	K
Co*	0.04	0.02	0.05	192	0.0006630	K
Ni*	0.06	0.03	0.04	228	0.0009424	K
Mo*	0.10	0.03	0.05	792	0.0012296	L
Ru	nd	nd				L
Rh	nd	nd				L
Pd	nd	nd				L
Os*	0.62	0.10	0.11	3504	0.0059903	M
Ir*	0.19	0.03	0.08	1127	0.0018908	M
Pt*	0.25	0.04	0.08	1529	0.0026002	M
Total	100.00	100.00				

Supplemental Figure 17. Scanning electron microscopy (SEM) and energy-dispersive x-ray spectroscopy (EDS) showing major and platinum-group elements (PGE) composition for spherule B from PU25 (Figure 14).

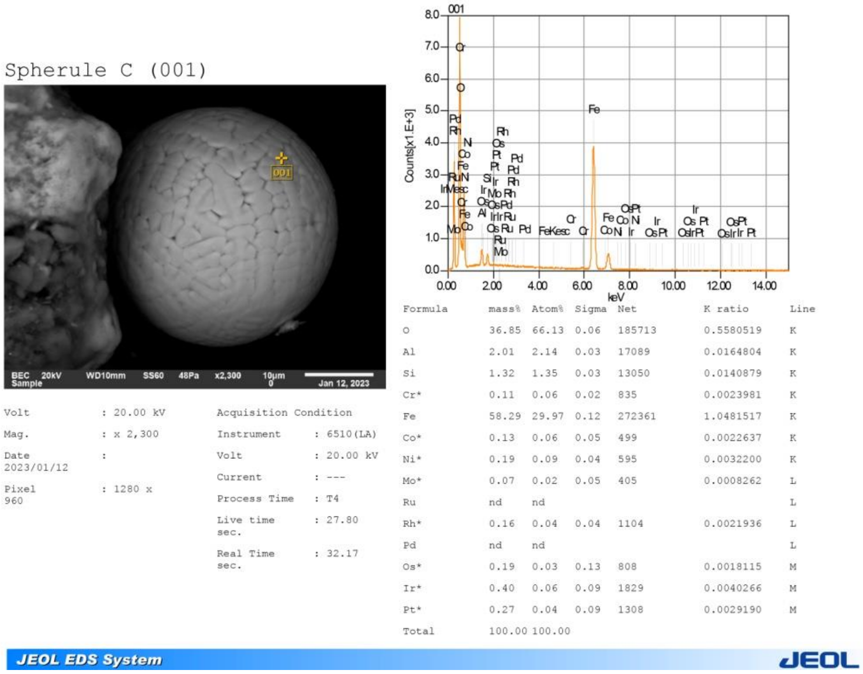

Supplementary Figure 18. Scanning electron microscopy (SEM) and energy-dispersive x-ray spectroscopy (EDS) showing major and platinum-group elements (PGE) composition for spherule C from PU25 in Figure 14.

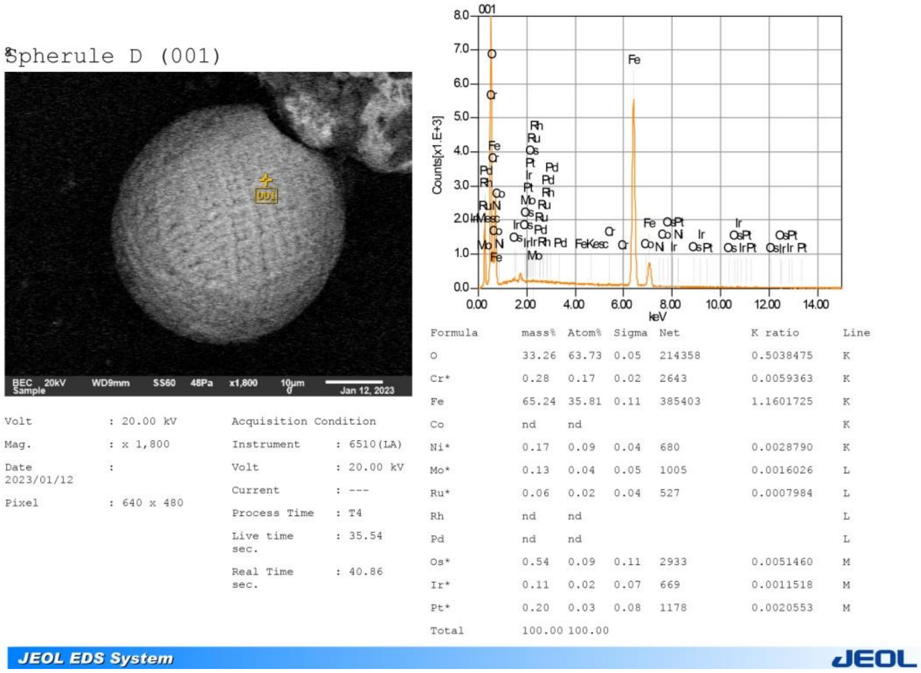

Supplementary Figure 19. Scanning electron microscopy (SEM) and energy-dispersive x-ray spectroscopy (EDS) showing major and platinum-group elements (PGE) composition for spherule D from PU25 in Figure 14.

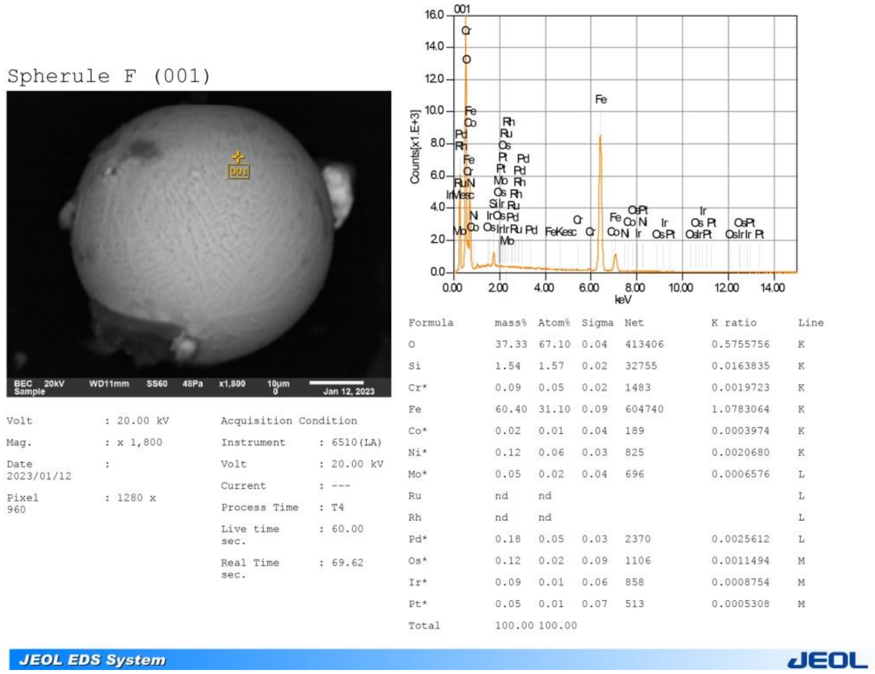

Formula	mass%	Atom%	Sigma	Net	K ratio	Line
O	37.33	67.10	0.04	413406	0.5755756	K
Si	1.54	1.57	0.02	32755	0.0163835	K
Cr*	0.09	0.05	0.02	1483	0.0019723	K
Fe	60.40	31.10	0.09	604740	1.0783064	K
Co*	0.02	0.01	0.04	189	0.0003974	K
Ni*	0.12	0.06	0.03	825	0.0020680	K
Mo*	0.05	0.02	0.04	696	0.0006576	L
Ru	nd	nd				L
Rh	nd	nd				L
Pd*	0.18	0.05	0.03	2370	0.0025612	L
Os*	0.12	0.02	0.09	1106	0.0011494	M
Ir*	0.09	0.01	0.06	858	0.0008754	M
Pt*	0.05	0.01	0.07	513	0.0005308	M
Total	100.00	100.00				

Supplementary Figure 20. Scanning electron microscopy (SEM) and energy-dispersive x-ray spectroscopy (EDS) showing major and platinum-group elements (PGE) composition for spherule F from PU25 in Figure 14.

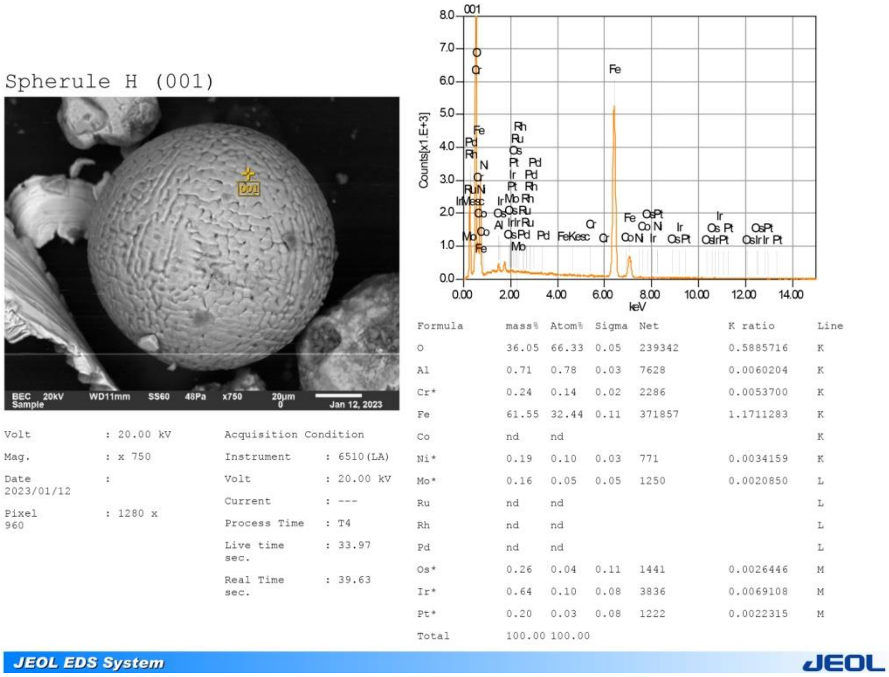

Formula	mass%	Atom%	Sigma	Net	K ratio	Line
O	36.05	66.33	0.05	239342	0.5885716	K
Al	0.71	0.78	0.03	7628	0.0060204	K
Cr*	0.24	0.14	0.02	2286	0.0053700	K
Fe	61.55	32.44	0.11	371857	1.1711283	K
Co	nd	nd				K
Ni*	0.19	0.10	0.03	771	0.0034159	K
Mo*	0.16	0.05	0.05	1250	0.0020850	L
Ru	nd	nd				L
Rh	nd	nd				L
Pd	nd	nd				L
Os*	0.26	0.04	0.11	1441	0.0026446	M
Ir*	0.64	0.10	0.08	3836	0.0069108	M
Pt*	0.20	0.03	0.08	1222	0.0022315	M
Total	100.00	100.00				

Supplementary Figure 21. Scanning electron microscopy (SEM) and energy-dispersive x-ray spectroscopy (EDS) showing major and platinum-group elements (PGE) composition for spherule H from PU25 in Figure 14.

Spherule I (001)

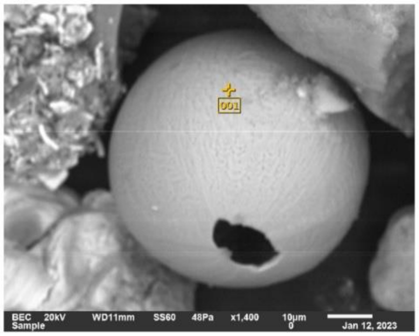

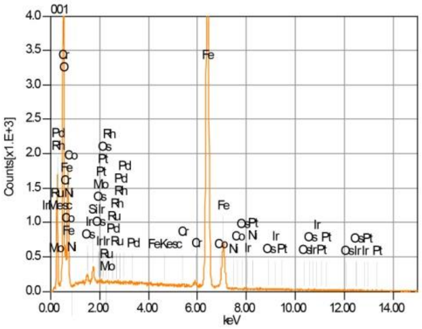

Volt	: 20.00 kV	Acquisition Condition		
Mag.	: x 1,400	Instrument	: 6510(LA)	
Date 2023/01/12	:	Volt	: 20.00 kV	
Pixel 960	: 1280 x	Current	: ---	
		Process Time	: T4	
		Live time sec.	: 31.54	
		Real Time sec.	: 35.62	

Formula	mass%	Atom%	Sigma	Net	K ratio	Line
O	26.72	55.81	0.05	136356	0.3611502	K
Si	0.85	1.01	0.03	8392	0.0079851	K
Cr*	0.24	0.15	0.02	1817	0.0045972	K
Fe	71.71	42.92	0.13	346471	1.1752490	K
Co	nd	nd				K
Ni	nd	nd				K
Mo*	0.14	0.05	0.05	847	0.0015219	L
Ru	nd	nd				L
Rh	nd	nd				L
Pd	nd	nd				L
Os	nd	nd				M
Ir*	0.25	0.04	0.07	1193	0.0023152	M
Pt*	0.09	0.02	0.07	444	0.0008734	M
Total	100.00	100.00				

JEOL EDS System JEOL

Supplementary Figure 22. Scanning electron microscopy (SEM) and energy-dispersive x-ray spectroscopy (EDS) showing major and platinum-group elements (PGE) composition for spherule I from PU25 in Figure 14.

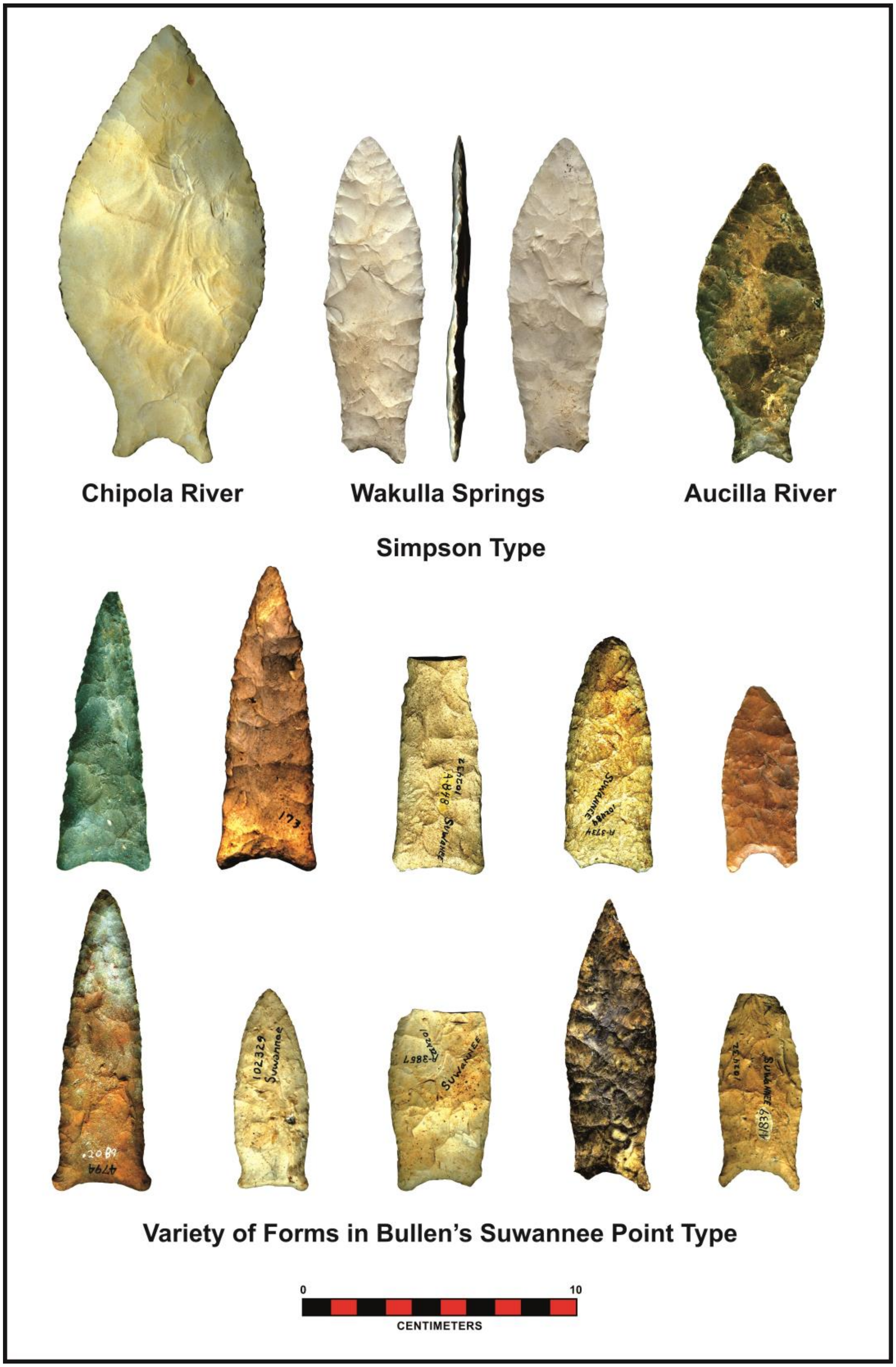

Supplementary Figure 23. Examples of various Simpson and Suwannee Paleoamerican point types common in Florida.

OSL Radial Graphs

Radial graphs for each OSL equivalent dose are given below for each unit measured: 1) reference with the yellow shading is the average age for the most abundant component of the finite mixture model; 2) reference with the blue shading is the average age for the second most abundant component; 3) the red line is the equivalent dose from the central age model. In some cases, the red line is not visible because the central age dose is close to one of the FMM doses, and the line is, therefore, obscured shading. Green shading is obtained where the blue and yellow shading overlap. Shaded areas encompass all points within two standard errors of the reference.

Radial graphs for Unit PU1

UW3689 (90 cmbs)

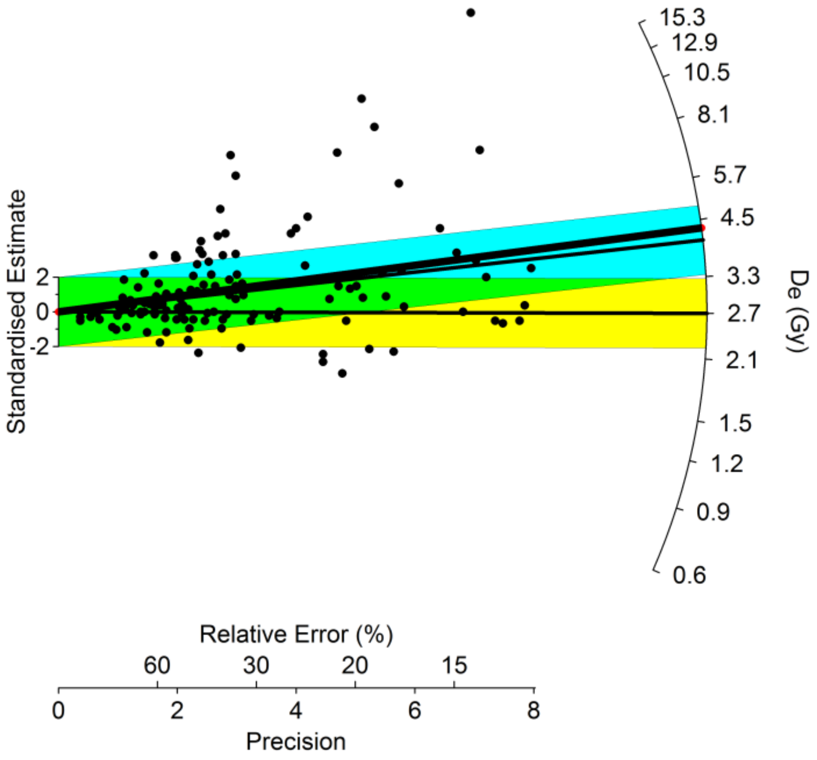

Supplementary Figure 24. Radial graph for UW3689 (90 cmbs) for PU1.

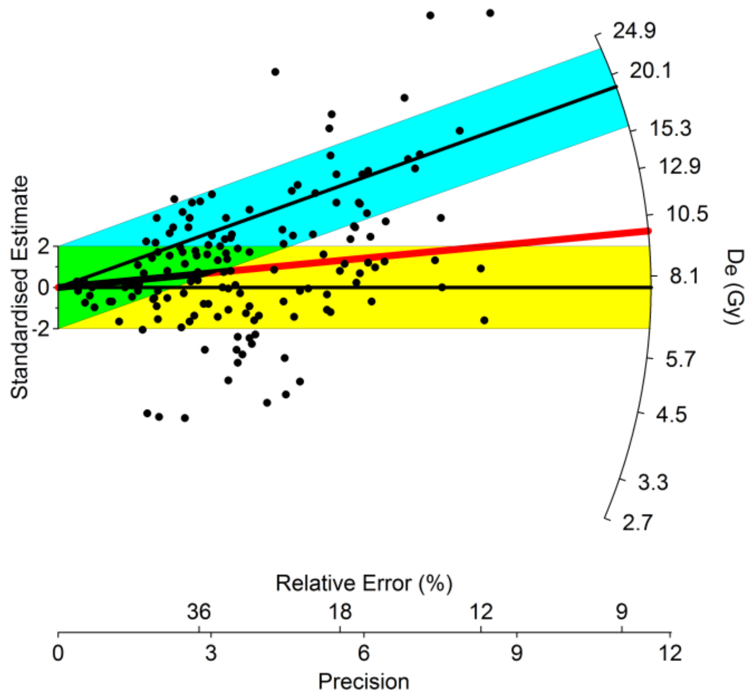

Supplementary Figure 25. Radial graph for UW3630 (130 cmbs) for PU1.

Radial graphs for KU4

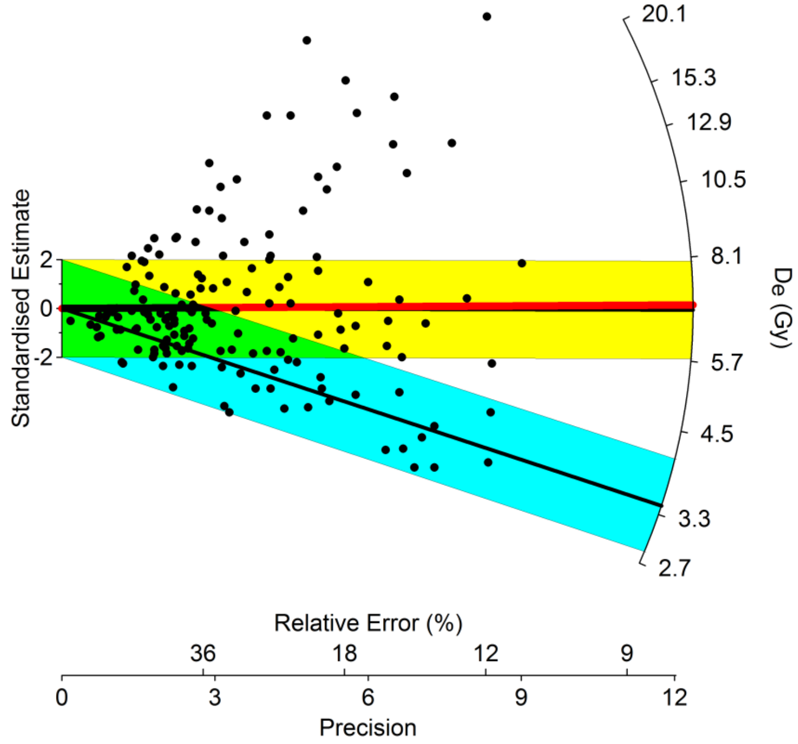

Supplementary Figure 26. Radial graph for UW3691 (90 cmbs) for KU4.

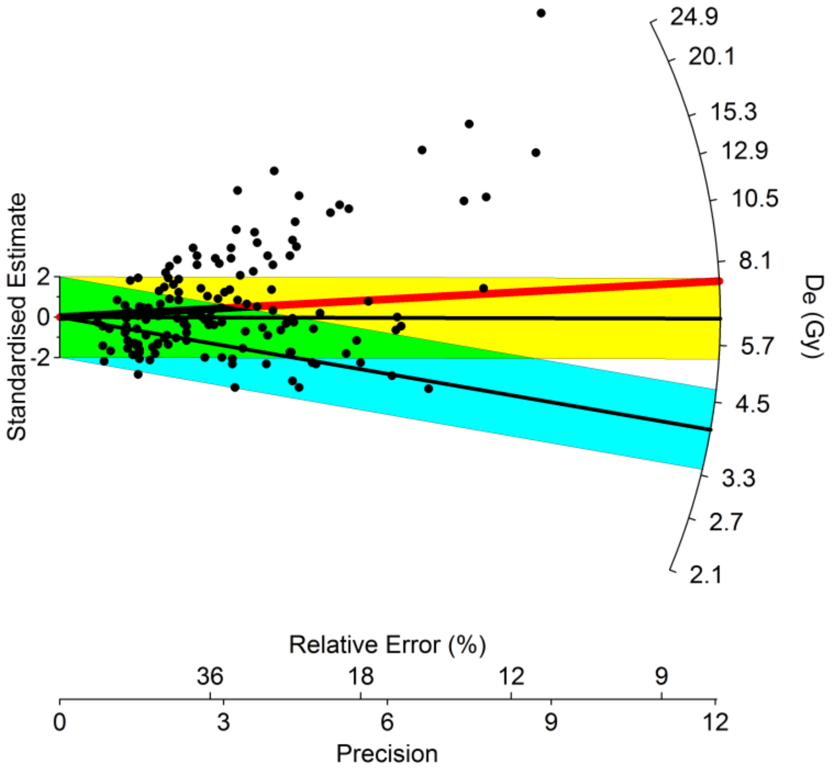

Supplementary Figure 27. Radial graph for UW3689 (96 cmbs) for KU4.

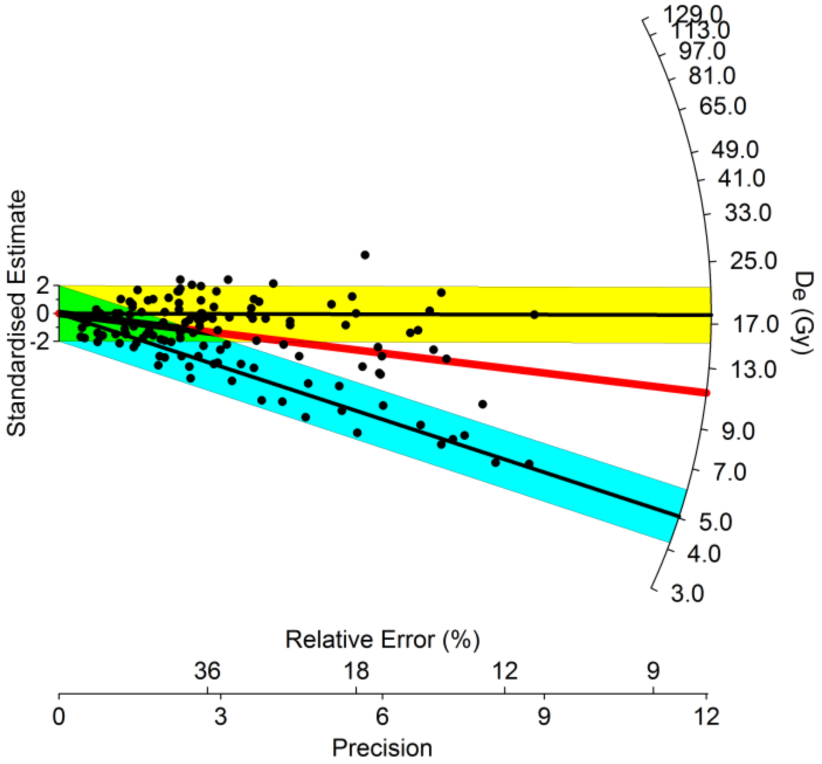

Supplementary Figure 28. Radial graph for UW3693 (111 cmbs) for KU4.

Radial graphs for PU15

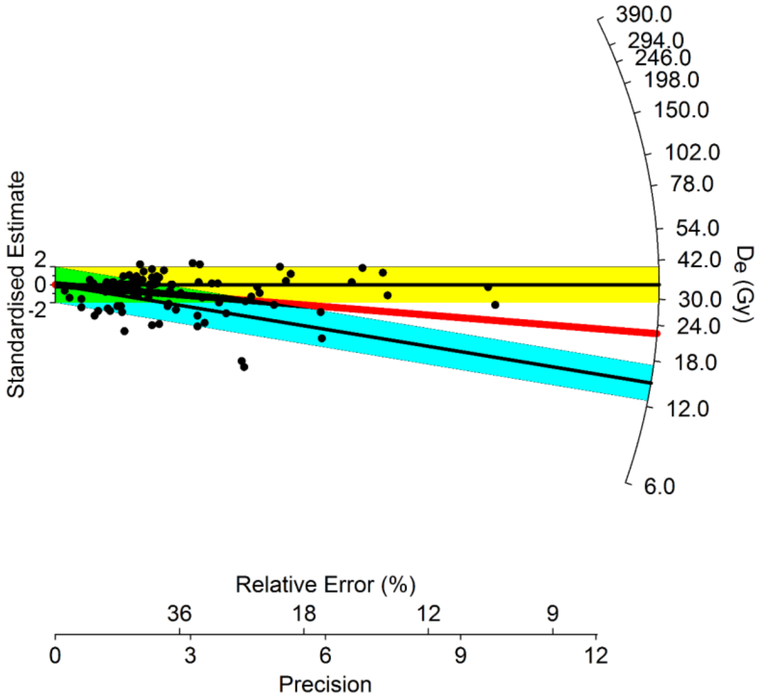

Supplementary Figure 29. Radial graph for UW3787 (102.5 cmbs) for PU15.

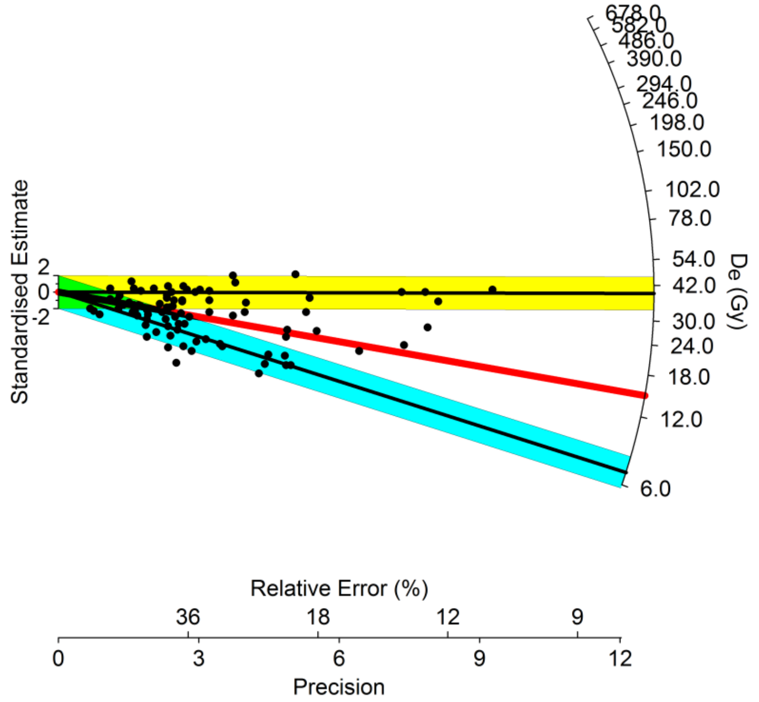

Supplementary Figure 30. Radial graph for UW3786 (120 cmbs) for PU15.

Radial graphs for PU7

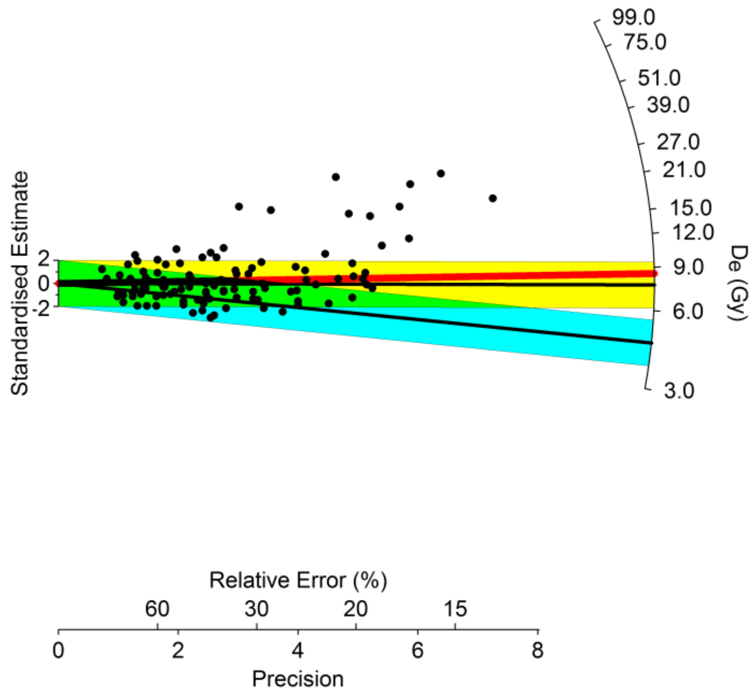

Supplementary Figure 31. Radial graph for UW3789 (63 cmbs) for PU7.

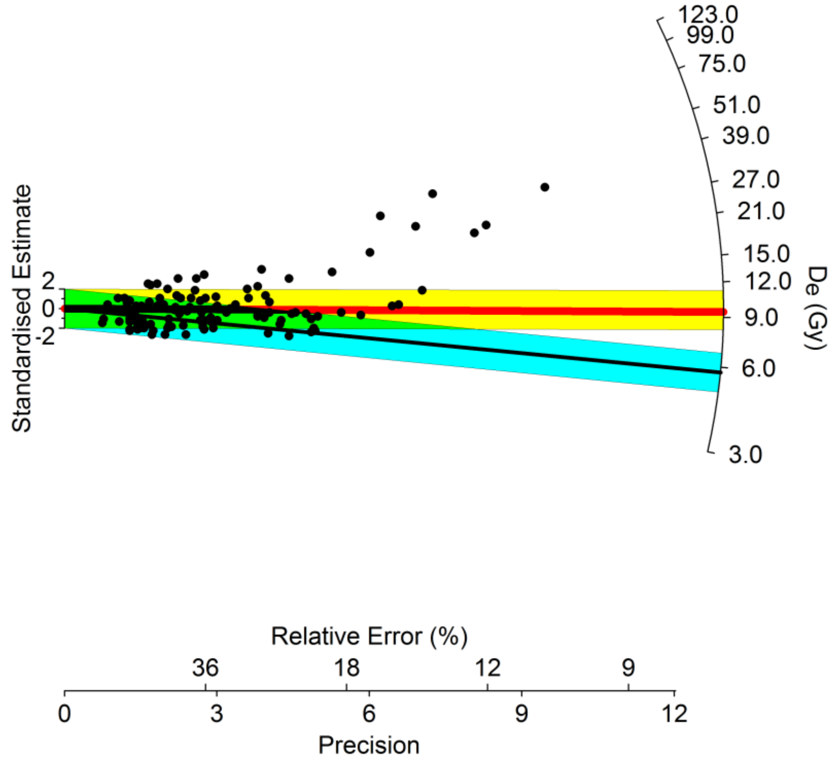

Supplementary Figure 32. Radial graph for UW3790 (80 cmbs) for PU7.

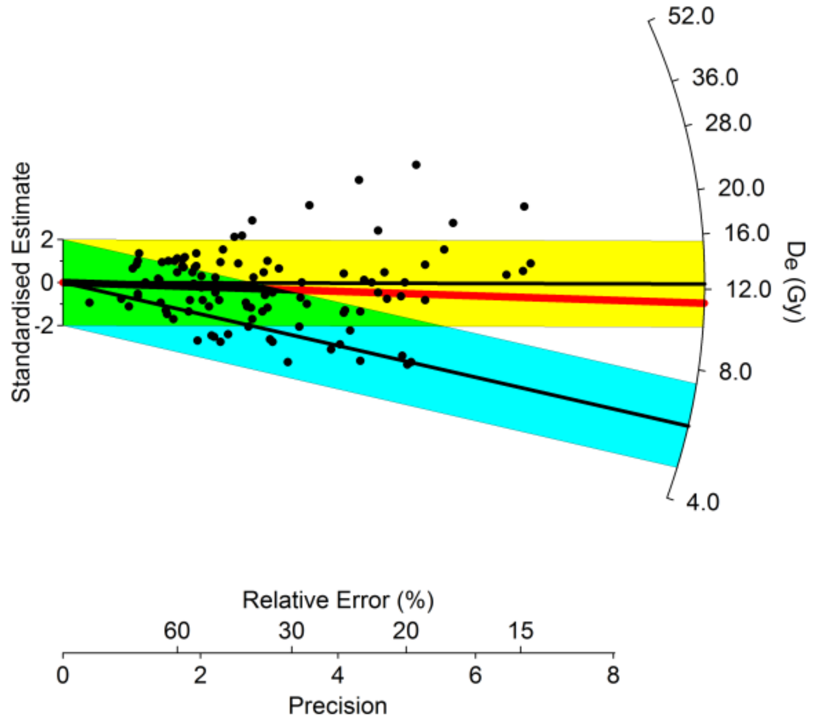

Supplementary Figure 33. Radial graph for UW3788 (103 cmbs) for PU7.

Radial graphs for PU23

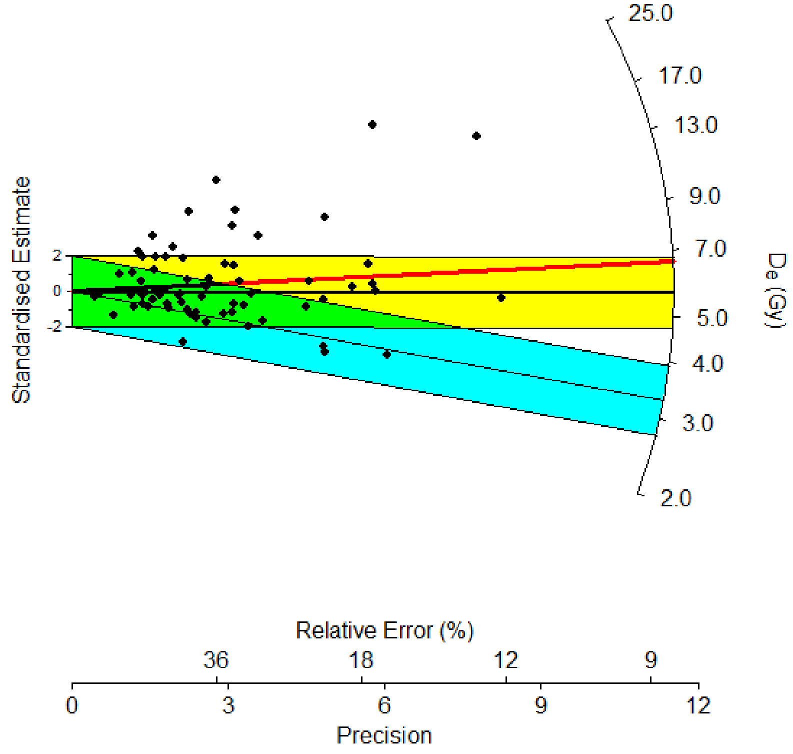

Supplementary Figure 34. Radial graph for UW3813 (90 cmbs) for PU23.

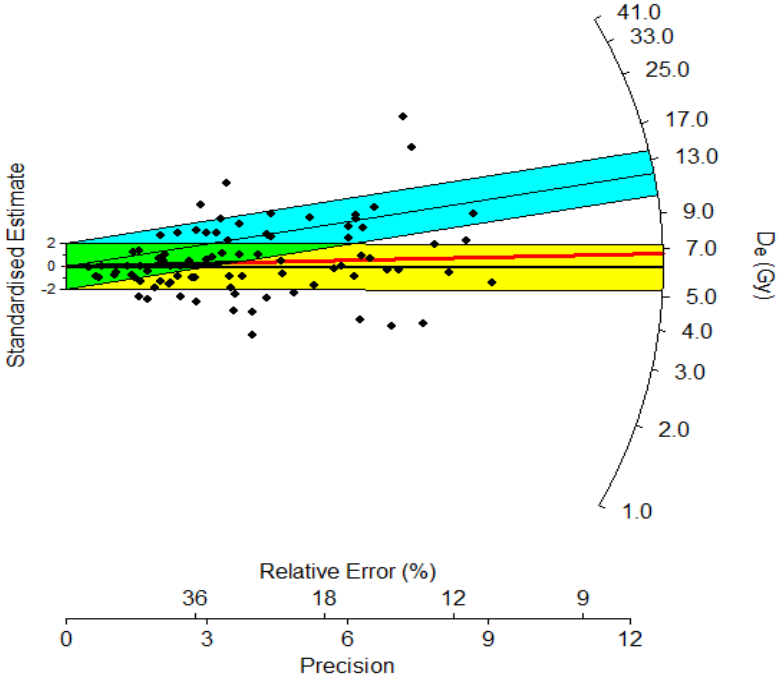

Supplementary Figure 35. Radial graph for UW3814 (103 cmbs) for PU23.

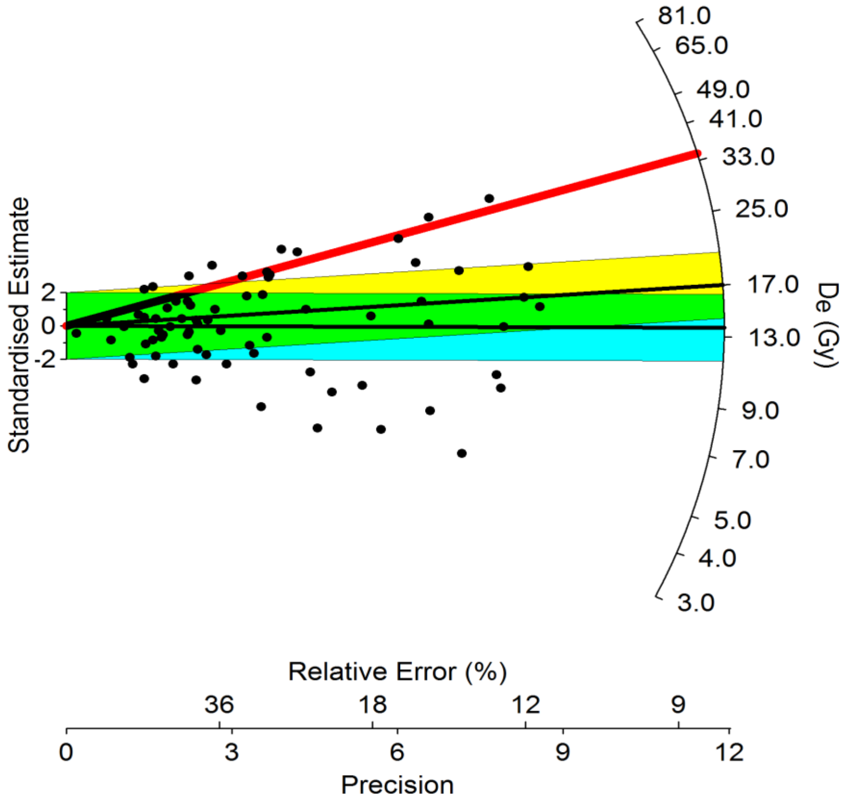

Supplementary Figure 36. Radial graph for UW3815 (120 cmbs) for PU23.

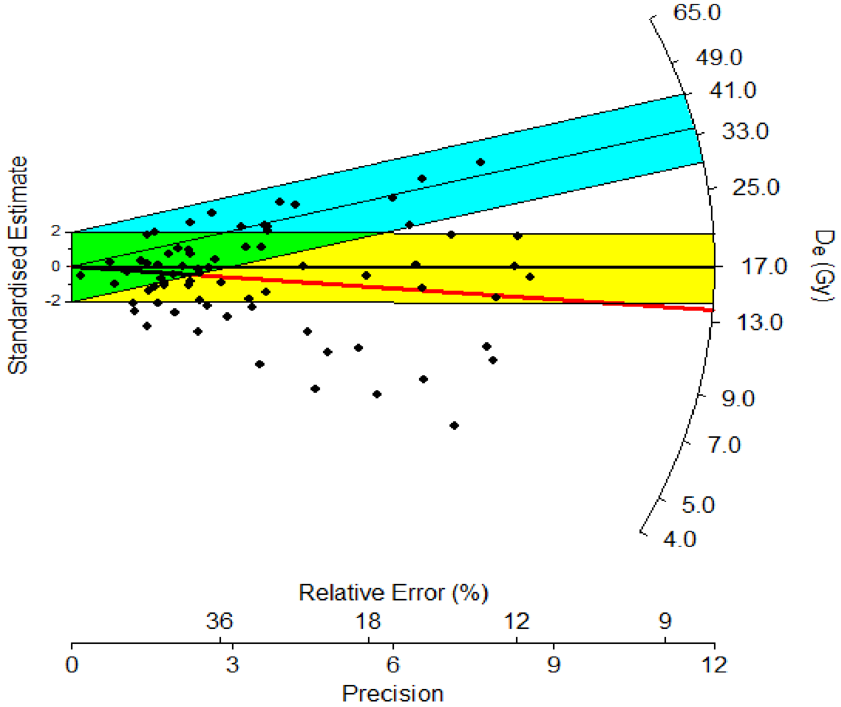

Supplementary Figure 37. Radial graph for UW3816 (185 cmbs) for PU23.

Radial graphs for WH1

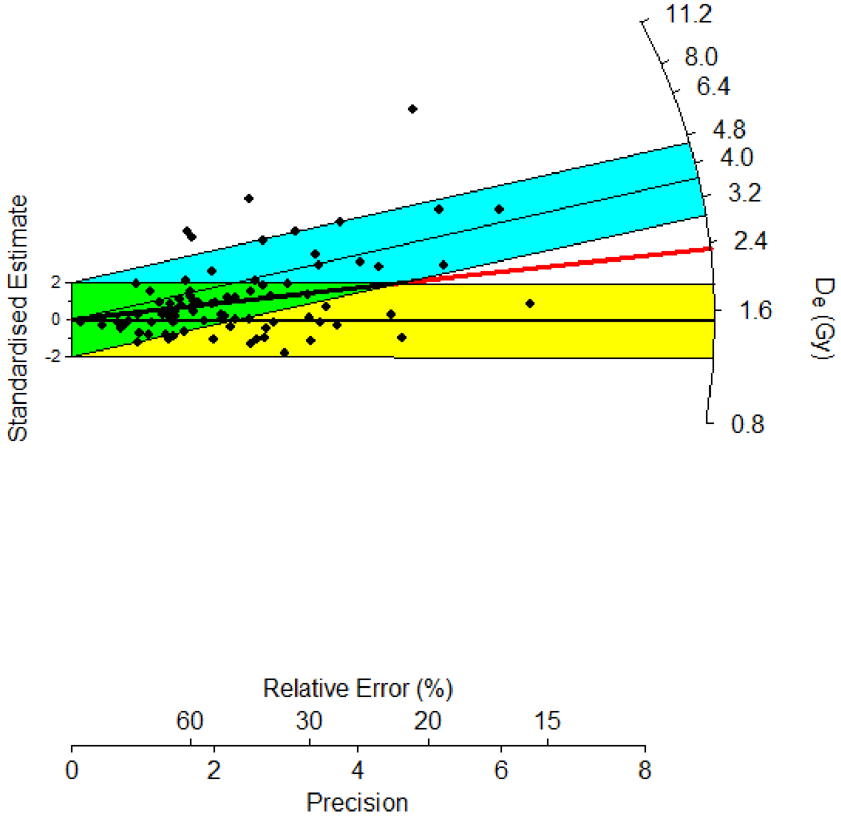

Supplementary Figure 38. Radial graph for UW3821 (35 cmbs) for WH1.

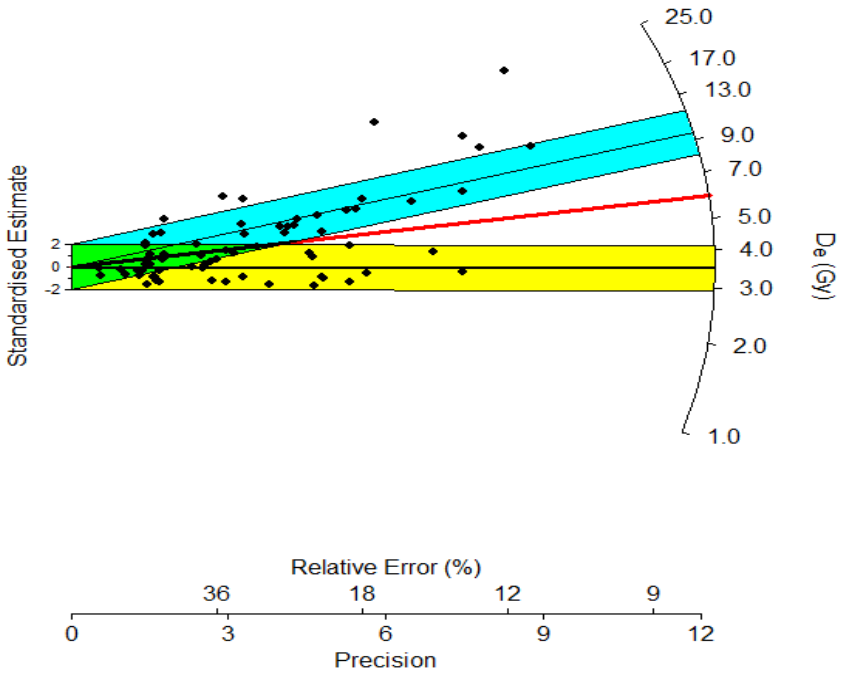

Supplementary Figure 39. Radial graph for UW3819 (100 cmbs) for WH1.

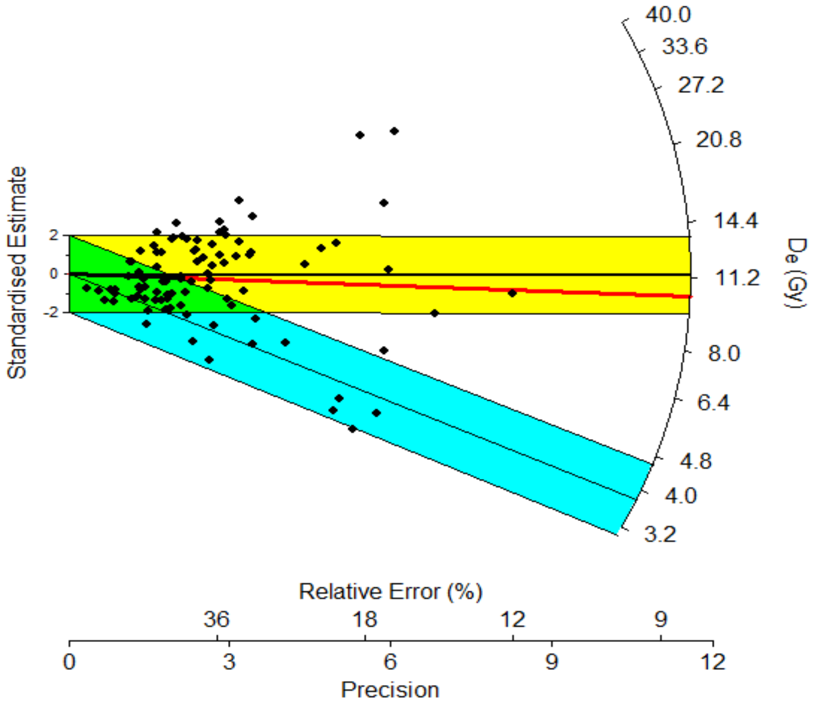

Supplementary Figure 40. Radial graph for UW3820 (140 cmbs) for WH1.

Supplemental Tables

Supplementary Table 1. List of important papers on the YDIH.

PROXY	PROPONENTS	INDEPENDENT WORKERS	CRITICS
**Cosmic impact spherules**	Firestone 2007, 2009, 2010; Bunch 2012; Israde 2012; Wittke 2013; LeCompte 2017; Pino 2018	Mahaney 2008, 2010a, 2010b, 2011a, 2011b, 2012; Redmond and Tankersley 2012; Fayek 2012; LeCompte 2012; Andronikov, 2016b	Surovell 2009; Haynes 2010; Pinter 2011; Pigati 2012; Boslough 2012; Holliday 2016; 2023
**Meltglass (scoria-like objects)**	Bunch 2012	Mahaney 2008, 2010a, 2010b, 2011a, 2011b, 2012; Fayek 2012	--
**Carbon sperules, glass-like carbon, aciniform carbon, PAHs, fullerenes**	Firestone 2007, 2009, 2010; Israde 2012; Maiorana-Boutilier 2016	Baker 2008; Redmond and Tankersley 2012; Mahaney 2010a, 2010b, 2011a, 2011b, 2012, 2013	Scott 2010; Pinter 2011; van Hoesel 2012, 2014; Boslough 2012
**Nanodiamonds**	Firestone 2007; Kennett 2009a, 2009b; Kurbatov 2010; Israde 2012; Kinzie 2014	Baker 2008; Tian 2010; Redmond and Tankersley 2012; Bement 2014	Daulton 2010; Pinter 2011; van Hoesel 2012, 2014; Boslough 2012
**Iridium**	Firestone 2007, 2009, 2010	Andronikov 2011; Marshall 2011; Andronikov 2016a	Paquay 2009; Haynes 2010; Pinter 2011; Pigati 2012; Boslough 2012
**Platinum**	Mahaney 2016; Moore 2017; LeCompte 2017; Pino 2018	Petaev 2013	Holliday 2023
**Osmium**	--	Beets 2008; Sharma 2009; Wu 2013	Paquay 2009
**Nickel, cobalt, chromium, thorium, ^{14}C, ^{10}Be, ^{26}Al, REEs**	Firestone 2007, 2009, 2010	Melott 2009; Andronikov 2014, 2015, 2016b, 2016c	--
**Impact-related biomass burning**	Firestone 2007; Wolbach 2018a, 2018b; Pino 2018	--	--
**Extinctions, human population declines**	Firestone 2007, 2009, 2010; Anderson 2011; Pino 2018	--	Marlon 2009

Full citations for papers are available in Kennett *et al.*[13] and Wolbach *et al.*[14].

Supplementary Table 2. Geochemical abundance data for PU1 (Au, Pt, and Pd).

Analyte Symbol	Au	Pt	Pd
Unit Symbol	ppb	ppb	ppb
Detection Limit	1	0.1	0.1
Analysis Method	FA-MS	FA-MS	FA-MS
Depth (cmbs)			
90-92.5	< 1	0.1	0.1
92.5-95	< 1	0.1	0.1
95-97.5	2	0.1	0.3
97.5-100	< 1	< 0.1	0.1
100-102.5	< 1	0.1	0.1
102.5-105	< 1	0.1	0.2
105-107.5	< 1	0.1	0.2
[a]107.5-110	8	0.7	1.1
110-112.5	< 1	< 0.1	0.1
112.5-115	3	< 0.1	< 0.1
115-117.5	< 1	< 0.1	0.1
117.5-120	2	< 0.1	0.1
120-122.5	< 1	0.1	0.1
122.5-125	5	< 0.1	< 0.1
125-127.5	< 1	0.1	0.1
127.5-130	< 1	0.1	0.1
130-132.5	3	< 0.1	0.1
132.5-135	< 1	0.1	0.1
135-137.5	< 1	0.1	0.1
137.5-140	4	0.2	0.2

[a]Sample with Pt peak for PU1.

Supplementary Table 3. Geochemical abundance data for PU7 (Au, Pt, and Pd).

Analyte Symbol	Au	Pt	Pd
Unit Symbol	ppb	ppb	ppb
Detection Limit	1	0.1	0.1
Analysis Method	FA-MS	FA-MS	FA-MS
Depth (cmbs)			
40-42.5	13	1.2	1.9
60-62.5	11	0.8	1.2
80-82.5	9	0.8	1.4
95-97.5	10	0.7	1.4
97.5-100	8	0.8	1.3
100-102.5	8	0.7	1.2
[a]102.5-105	15	6	9.6
105-107.5	9	0.6	1.3
107.5-110	27	1.1	3.4
110-112.5	9	0.7	1.4
112.5-115	9	0.7	1.5
115-117.5	11	0.9	2
117.5-120	8	0.7	1.3
120-122.5	9	0.7	1.6
122.5-125	8	0.7	1.2
125-127.5	12	0.8	1.4
127.5-130	17	1.5	1.9

[a]Sample with Pt peak for PU7

Supplementary Table 4. Geochemical abundance data for KU4 samples (Au, Pt, and Pd).

Analyte Symbol	Au	Pt	Pd	Pt/Pd
Unit Symbol	ppb	ppb	ppb	
Detection Limit	1	0.1	0.1	
Analysis Method	FA-MS	FA-MS	FA-MS	
Depth (cmbs)				
75-77.5	132	0.4	1.2	0.33
90-92.5	147	0.7	1.1	0.64
92.5-95	122	0.7	1.2	0.58
95-97.5	115	1.1	1.3	0.85
97.5-100	120	0.8	1.3	0.62
100-102.5	199	1.2	1.3	0.92
102.5-105	69	0.6	1.4	0.43
[a]105-107.5	171	1.3	1.2	1.08
107.5-110	118	0.4	1.2	0.33
110-112.5	141	0.5	0.8	0.63
115-117.5	123	0.5	1.1	0.45

[a]Sample with Pt and Pt/Pd peak for KU4.

Supplementary Table 5. Geochemical abundance data for PU15 (Au, Pt, and Pd).

Analyte Symbol	Au	Pt	Pd
Unit Symbol	ppb	ppb	ppb
Detection Limit	1	0.1	0.1
Analysis Method	FA-MS	FA-MS	FA-MS
Depth (cmbs)			
40-42.5	6	< 0.1	< 0.1
50-52.5	88	0.8	1.3
70-72.5	20	0.7	1.3
90-92.5	29	0.7	1.3
92.5-95	17	0.7	4.2
95-97.5	20	0.8	1.3
[a]97.5-100	28	1.1	3.8
100-102.5	45	0.8	1.1
102.5-105	22	0.6	1.1
105-107.5	34	1	1.7
107.5-110	50	0.9	1.2
110-112.5	18	0.7	2.3
112.5-115	32	0.8	2
115-117.5	22	0.6	1.2
117.5-120	18	0.9	1.5

[a]Sample with Pt peak for PU15.

Supplementary Table 6. Geochemical abundance data for PU23 samples (Au, Pt, and Pd).

Analyte Symbol	Au	Pt	Pd	Pt/Pd
Unit Symbol	ppb	ppb	ppb	
Detection Limit	1	0.1	0.1	
Analysis Method	FA-MS	FA-MS	FA-MS	
Depth (cmbs)				
90 to 92.5 cm	24	0.6	0.7	0.9
92.5 to 95 cm	76	0.5	0.9	0.6
95 to 97.5 cm	42	0.4	0.7	0.6
[a]97.5 to 100 cm	27	0.9	0.7	1.3
100 to 102.5 cm	25	0.7	0.8	0.9
102.5 to 105 cm	58	0.4	0.6	0.7
105 to 107.5 cm	87	0.3	0.6	0.5
107.5 to 110 cm	171	0.5	0.7	0.7
110 to 112.5 cm	78	0.4	0.7	0.6
112.5 to 115 cm	97	0.6	0.6	1.0
115 to 117.5 cm	60	0.5	0.7	0.7
117.5 to 120 cm	39	0.5	0.6	0.8
120 to 122.5 cm	29	0.6	0.8	0.8
122.5 to 125 cm	59	0.4	0.8	0.5
130 to 132.5 cm	73	0.6	0.6	1.0
140 to 142.5 cm	63	0.5	1.2	0.4

[a]Pt and Pt/Pd peak anomaly for PU23.

Supplementary Table 7. Geochemical abundance data for PU25 (Au, Pt, and Pd).

Analyte Symbol	Au	Pt	Pd	Pt/Pd
Unit Symbol	ppb	ppb	ppb	
Detection Limit	1	0.1	0.1	
Analysis Method	FA-MS	FA-MS	FA-MS	
Depth (cmbs)				
80 to 82.5 cm	26	0.7	0.7	1.0
82.5 to 85 cm	15	0.8	0.5	1.6
85 to 87.5 cm	17	0.4	0.7	0.6
87.5 to 90 cm	16	0.6	0.7	0.9
90 to 92.5 cm	17	0.4	0.9	0.4
92.5 to 95 cm	22	0.5	0.8	0.6
95 to 97.5 cm	13	0.5	0.4	1.3
97.5 to 100 cm	17	0.6	0.6	1.0
100 to 102.5 cm	19	0.9	0.7	1.3
102.5 to 105 cm	16	0.5	0.6	0.8
105 to 107.5 cm	15	0.4	0.6	0.7
107.5 to 110 cm	39	0.4	0.9	0.4
110 to 112.5 cm	16	0.7	0.6	1.2
112.5 to 115 cm	18	0.6	0.8	0.8
115 to 117.5 cm	15	0.4	0.5	0.8
117.5 to 120 cm	31	0.5	0.8	0.6
120 to 122.5 cm	19	0.6	0.8	0.8
122.5 to 125 cm	17	0.5	0.7	0.7
130 to 132.5 cm	16	0.5	1.7	0.3
140 to 142.5 cm	17	0.7	0.6	1.2
147.5 to 150 cm	17	0.4	0.7	0.6

[a]Pt peak anomaly sample for PU25

Supplementary Table 8. Geochemical abundance data for WH1 (Au, Pt, and Pd).

Analyte Symbol	Au	Pt	Pd
Unit Symbol	ppb	ppb	ppb
Detection Limit	1	0.1	0.1
Analysis Method	FA-MS	FA-MS	FA-MS
Depth (cmbs)			
10 to 12.5 cm	32	1	1.3
30 to 32.5 cm	34	0.7	1.3
50 to 52.5 cm	607	0.6	1.4
70 to 72.5 cm	41	0.8	1.6
90 to 92.5 cm	46	0.7	0.8
95 to 97.5 cm	35	0.7	1.2
97.5 to 100 cm	25	0.7	1.1
100 to 102.5 cm	54	0.7	0.9
102.5 to 105 cm	21	0.8	0.9
105 to 107.5 cm	43	0.5	0.9
107.5 to 110 cm	23	0.7	0.9
110 to 112.5 cm	19	0.8	1
112.5 to 115 cm	27	0.5	0.8
115 to 117.5 cm	25	0.5	0.6
117.5 to 120 cm	31	0.8	1.1
120 to 122.5 cm	27	0.4	0.8
122.5 to 125 cm	27	0.7	0.8
125 to 127.5 cm	21	0.4	0.7
127.5 to 130 cm	31	0.5	1

Supplementary Table 9. Spherules and spherules/kilogram for PU23.

Sample Depth	Sample Mass (g)	Spherules	Spherules/Kg
87.5 to 90	362	30	82.9
90 to 92.5	368	30	81.5
92.5 to 95	409	51	124.7
95 to 97.5	397	27	68.0
[a]97.5 to 100	399	40	100.3
100 to 102.5	373	6	16.1
102.5 to 105	421	6	14.3
105 to 107.5	415	14	33.7
110 to 112.5	372	8	21.5
120 to 122.5	359	15	41.8
130 to 132.5	414	14	33.8
135 to 137.5	402	10	24.9

[a]Sample with Pt peak for PU23.

Supplementary Table 10. Spherules and spherules/kilogram for PU25.

Sample Depth	Sample Mass (g)	Spherules	Spherules/Kg
80 to 82.5	318	16	50.3
90 to 92.5	416	11	26.4
92.5 to 95	370	4	10.8
95 to 97.5	372	6	16.1
97.5 to 100	399	28	70.2
[a]100 to 102.5	407	7	17.2
102.5 to 105	350	32	91.4
105 to 107.5	341	9	26.4
107.5 to 110	507	6	11.8
110 to 112.5	460	4	8.7
112.5 to 115	410	4	9.8
120 to 122.5	372	1	2.7
127.5 to 130	459	4	8.7
130 to 132.5	298	12	40.3
132.5 to 135	513	5	9.7
140 to 142.5	393	9	22.9

[a]Sample with Pt peak for PU25.

Supplementary Table 11. High-temperature minerals on microspherules from Wakulla Springs.

Mineral	Spherule name	Laboratory	C (wt%)	O	Al	Si	Ti	Cr	Fe	Co	Ni	Mo	Ru (ppm)	Rh	Pd	Os	Ir	Pt
Co-Ni-Fe oxide	PU1-107_site1(5)_pt1	U. of Oregon	30.17	29.92	1.11	2.36	0	0	35.30	0.1	0.25	0	800	800	0	400	100	900
Co-Ni-Fe oxide	PU1-107_site1(5)_pt2	U. of Oregon	38.94	34.77	2.41	2.17	0	0	20.31	0.03	0.07	0	1200	0	0	0	1300	300
Cr-Fe oxide	PU1-107_site1(2)_pt1	U. of Oregon	1.92	24.9	0.56	0.82	0	0.16	70.72	0	0	0	500	0	0	0	800	0
Cr-Fe oxide	PU1-107_site1(4)_pt2	U. of Oregon	1.55	24.25	0.24	0.28	0	0.16	72.60	0	0	0	0	0	0	400	0	1300
Cr-Fe oxide	PU1-155-site 1(1)_pt1	U. of Oregon	0	29.13	0.4	0.44	0.09	0.14	68.18	0	0	0	500	500	100	2700	3700	1000
Cr-Fe oxide	PU1-155-site 1(1)_pt3	U. of Oregon	0	25.8	0.32	0.33	0	0.06	70.89	0	0	0.06	900	0	1300	0	8700	5600
Cr-Fe oxide	PU7-102-site 3 (1)_pt2	U. of Oregon	3.45	26.22	0.37	0.33	0.11	0.07	68.73	0	0	0	300	100	200	0	0	0
Cr-Fe oxide	PU7-102-site 6 (1)_pt1	U. of Oregon	0	28.2	0.46	0.52	0	0.03	69.76	0	0	0.06	1100	0	600	0	0	0
Cr-Fe oxide	PU7-132_(2)_pt1	U. of Oregon	1.46	27.18	0.31	0.36	0	0.07	70.1	0	0	0	0	0	0	0	0	0
Cr-Fe oxide	PU7-132-site 1 particle(1)_pt1	U. of Oregon	0	27.99	0.32	0.52	0	0.09	69.43	0	0	0	0	1100	0	0	0	0
Cr-Fe oxide	PU7-132-site 1 particle(1)_pt2	U. of Oregon	0	25.34	0.22	0.27	0	0.14	73.32	0	0	0	0	0	0	0	5100	2000
Cr-FeO (wustite)	PU7-102-site 4 (1)_pt1	U. of Oregon	0	21.18	0.25	0.26	0	0.05	76.24	0	0	0	800	0	0	0	0	2000
Cr-FeO (wustite)	PU7-102-site 6 (1)_pt3	U. of Oregon	0	19.71	0.26	0.27	0	0.07	78.20	0	0	0.01	700	0	0	900	0	0
Cr-FeO (wustite)	PU7-102-sphere1(1)_pt1	U. of Oregon	2.22	19.63	0.24	0.28	0	0.02	75.94	0	0	0.07	800	0	0	900	2700	4000
Cr-Ni-Co-Fe oxide	PU1-107-site 2(1)_pt1	U. of Oregon	0	29.25	0.79	0.79	0	0.13	67.99	0.04	0.15	0	0	0	0	0	0	0
Cr-Ni-Fe oxide	PU1-155-site 1(2)_pt1	U. of Oregon	0	25.67	0.66	0.83	0	0.08	71.23	0	0.06	0.13	500	200	300	4900	0	0
Cr-Ni-Fe oxide	PU7-102-site 3 (1)_pt1	U. of Oregon	2.15	24.83	0.28	0.28	0.08	0.11	71.11	0	0.05	0	0	900	0	3100	0	0
Cr-Ni-Fe oxide	PU7-102-sphere2L(1)_pt1	U. of Oregon	1.68	22.88	0.28	0.65	0	0.33	72.82	0	0.12	0.05	0	400	200	0	0	2500
Cr-Ni-Fe, native	PU1-155-site 1(2)_pt2	U. of Oregon	0	1.58	0.03	0.06	0	0.16	96.54	0	0.05	0.05	300	0	200	1300	4100	700
Cr-Ni-FeO (wustite)	PU1-155-site 1(1)_pt2	U. of Oregon	0	20.69	0.22	0.23	0	0.09	76.20	0	0.09	0	500	300	0	6700	1700	0
Cr-Ni-FeO (wustite)	PU7-102-site 5 (1)_pt1	U. of Oregon	0	13.87	0.23	0.39	0	0.29	82.54	0	0.22	0	0	0	0	1400	3600	6500
Cr-Ni-FeO (wustite)	PU7-102-sphere2R(1)_pt1	U. of Oregon	1.95	21.84	0.32	3.82	0	0.23	70.66	0	0.04	0	0	0	0	0	0	0
Cr-Ni-FeO (wustite)	PU7-132_(2)_pt2	U. of Oregon	1.96	20.55	0.23	0.18	0	0.4	76.16	0	0.07	0	0	0	0	1300	600	0
Fe oxide	PU1-107_site1(1)_pt1	U. of Oregon	1.5	28.62	0.27	0.32	0	0	68.01	0	0	0	500	0	500	0	800	0
Fe oxide	PU1-107_site1(3)_pt1	U. of Oregon	1.83	27.16	0.74	1.29	0	0	67.98	0	0	0	700	0	700	0	400	0
Fe oxide	PU1-107_site1(5)_pt3	U. of Oregon	1.41	29.4	1.64	2.41	0	0	64.33	0	0	0	400	0	0	0	1000	0
Fe oxide	PU7-102-site 3 (1)_pt3	U. of Oregon	1.28	24.48	0.19	0.4	0.17	0	71.68	0	0	0	0	0	0	4000	2700	2200
Cr-Ni-Fe oxide	PU1-155-site 1(1)_pt5	U. of Oregon	0	7.43	0.53	2.03	0.29	17.5	62.46	0	8.41	0.2	600	500	0	0	0	0
Cr-Ni-Fe oxide	PU7 132_(2)_pt3	U. of Oregon	2.48	2.57	0.26	0.47	0	19.21	63.8	0	9.78	0.14	0	0	0	200	300	1200
Ni-Co-Fe oxide	PU1-155-site 1(1)_pt4	U. of Oregon	0	7.98	0.99	0.96	0	5.79	80.06	0.11	2.77	0.05	1900	0	0	0	0	0
Ni-Co-Fe oxide	PU7-132-site 1 particle(1)_pt4	U. of Oregon	0	33.36	3.21	0.97	0.88	29.56	29.40	0.06	0.14	0.06	200	600	0	0	0	300
Ni-Fe oxide	PU1-107_site1(4)_pt1	U. of Oregon	2.17	29.31	0.16	0.32	0	0	66.91	0	0.21	0	200	0	1000	0	600	0
Ni-Fe oxide	PU7-102_(1)_pt1	U. of Oregon	1.98	25.53	1.54	1.6	0	0	68.63	0	0.04	0	0	200	300	0	0	0
Ni-Fe oxide	PU7-132_(1)_pt1	U. of Oregon	2.26	27.46	0.26	0.2	0	0	68.59	0	0.04	0	0	0	300	800	0	1100
Co-Ni-Fe oxide	230112_P25_Sph_05_45um_PEDS_ALL_El01	Elizabeth City St. U.	--	36.24	0	0	0	0	62.51	0.04	0.06	0.1	0	0	0	6200	1900	2500
Cr-Co-Fe oxide	230112_P25_Sph_01_51um_PEDS_ALL_El06	Elizabeth City St. U.	--	34.34	0	1.47	0	0.07	62.93	0.4	0	0	1100	400	900	2900	600	2000
Cr-Co-Ni-Fe oxide	230112_P25_Sph_04_35um_PEDS_All_El01	Elizabeth City St. U.	--	36.85	2.01	1.32	0	0.11	58.29	0.13	0.19	0.07	0	1600	0	1900	4000	2700
Cr-Co-Ni-Fe oxide	230112_P25_Sph_01_51um_PEDS_ALL_El07	Elizabeth City St. U.	--	24.94	0	1.99	0	0.1	72.54	0.07	0.06	0	0	0	0	1300	1800	0
Cr-Co-Ni-Fe oxide	230112_P25_Sph_07_48um_PEDS_ALL_El01	Elizabeth City St. U.	--	37.33	0	1.54	0	0.09	60.4	0.02	0.12	0.05	0	0	1800	1200	900	500
Cr-Co-Ni-titanomagnetite	230119_P23_Ovoid_01_48um_zm01_AEDS_ALL_El01_Ir_0,9	Elizabeth City St. U.	--	24.46	0	0	18.87	7.28	40.73	0.04	5.56	1.02	0	0	500	7600	8700	3600
Cr-Co-Ni-titanomagnetite	230119_P23_Ovoid_01_90um_zm02A_PEDS_ALL_El01_Os_0,6	Elizabeth City St. U.	--	42.89	0	0	32.74	0.08	22.01	0.03	0.07	0	0	0	200	6100	2600	4300
Cr-Fe oxide	230112_P25_Sph_09_58um_PEDS_ALL_El01	Elizabeth City St. U.	--	26.72	0	0.85	0	0.24	71.71	0	0	0.14	0	0	0	0	2500	900
Cr-Fe oxide	230119_P23_Sph_03_55um_AEDS_ALL_El01	Elizabeth City St. U.	--	36.02	0	1.26	0	0.11	62.03	0	0	0.14	0	700	0	0	3800	0
Cr-Ni-Fe oxide	230119_P23_Sph_04_58um_PEDS_ALL_El01_Os_0,7	Elizabeth City St. U.	--	34.07	0	0	0	0.14	63.92	0	0.17	0	0	0	700	7400	4100	4700
Cr-Ni-Fe oxide	230112_P25_Sph_02_109um_PEDS_ALL_El01	Elizabeth City St. U.	--	35.11	0	0	0	0.15	64.2	0	0.03	0	800	0	0	1700	2000	600
Cr-Ni-Fe oxide	230119_P23_Sph_01_54um_PEDS_ALL_El01	Elizabeth City St. U.	--	36.49	0	1.38	0	0.41	60.97	0	0.34	0.18	0	0	500	1000	900	0
Cr-Ni-Fe oxide	230112_P25_Sph_03_40um_PEDS_ALL_El01	Elizabeth City St. U.	--	33.26	0	0	0	0.28	65.24	0	0.17	0.13	600	0	0	5400	1100	2000
Cr-Ni-Fe oxide	230119_P23_Sph_02_48um_zm01_AEDS_ALL_El01	Elizabeth City St. U.	--	29.56	0	1.41	0	0.12	68.35	0	0.09	0.06	300	0	1000	900	1900	0
Cr-Ni-Fe oxide	230112_P25_Sph_01_51um_PEDS_ALL_El01	Elizabeth City St. U.	--	33.65	0	1.4	0	0.1	64.1	0	0.18	0.03	0	0	1000	2200	2100	0
Cr-Ni-Fe oxide	230119_P23_Sph_05_46um_PEDS_ALL_El01	Elizabeth City St. U.	--	37.81	0	0.99	0	0.07	60.76	0	0.19	0.12	0	0	0	0	600	0
Cr-Ni-FeO (wustite)	230112_P25_Obj_01_300um_PEDS_El01	Elizabeth City St. U.	--	11.44	0	0	0	0.37	86.24	0	0.24	0.14	0	0	0	8300	2400	4900
Cr-Ni-FeO (wustite)	230119_P23_Sph_05_46um_PEDS_ALL_El02_w C	Elizabeth City St. U.	--	8.14	0.51	1.72	0	0.23	88.9	0	0.2	0	0	0	400	1200	1400	0
Cr-Ni-FeO (wustite)	230112_P25_Sph_01_51um_PEDS_ALL_El05	Elizabeth City St. U.	--	9.08	0	1.44	0	0.1	88.7	0	0.16	0.13	800	0	100	1900	0	900
Cr-Ni-FeO (wustite)	230112_P25_Sph_01_51um_PEDS_ALL_El02	Elizabeth City St. U.	--	8.23	0	1.7	0	0.08	88.98	0	0.1	0	900	2500	800	2600	1800	600
Cr-Ni-FeO (wustite)	230112_P25_Sph_01_51um_PEDS_ALL_El04	Elizabeth City St. U.	--	9.33	0	1.74	0	0.02	88.6	0	0.05	0	0	1000	400	300	800	200
Cr-Ni-titanomagnetite	230119_P23_Ovoid_01_48um_zm01_AEDS_ALL_El01_Os_1,0	Elizabeth City St. U.	--	22.8	0	0	17.82	7.66	42.22	0	5.85	1.15	0	800	400	10300	7500	6000
Ni-Fe oxide	230112_P25_Sph_08_107um_PEDS_ALL_El01	Elizabeth City St. U.	--	36.05	0.71	0.24	0	0	61.55	0	0.19	0.16	0	0	0	2600	6400	2000
Ni-FeO (wustite)	230112_P25_Sph_01_51um_PEDS_ALL_El03	Elizabeth City St. U.	--	8.51	0	1.61	0	0	89.5	0	0.11	0	800	0	0	100	1700	100
	**TOTALS**		**3.01**	**24.86**	**0.41**	**0.85**	**1.23**	**1.60**	**67.45**	**0.02**	**0.63**	**0.08**	**322**	**217**	**248**	**1760**	**1719**	**1209**
	**RATIOS**		**13/34**	**58/58**	**37/58**	**50/58**	**7/58**	**47/58**	**58/58**	**12/58**	**39/58**	**24/58**	**27/58**	**17/58**	**25/58**	**35/58**	**50/58**	**27/58**

Supplementary Table 12. Melting points for high-temperature minerals.

Phase	Formula	~Equilib Melt T (°C)
**Chromite**	$(Fe)Cr_2O_4$	2265
**Chromium**	Cr2FeO4	1907
**Cobalt**	Co	1495
**Cr-magnetite**	Cr_2FeO_4	1670
**Fe oxide (magnetite)**	Fe_3O_4	1590
**Fe, native**	Fe	1538
**Iridium**	Ir	2466
**Nickel**	Ni	1455
**Nickel iron**	NiFe	1430
**Platinum**	Pt	1768
**Quartz**	SiO_2	1720
**Sediment**	Si-Ca-rich	1250
**Spherules, Fe-rich**	Fe + Fe oxides	1420
**Spherules, Si-rich**	Si, Ca, Al, Fe oxides	1250
**Titanomagnetite**	$TiFe_2O_4$	1625

Bayesian Data

Supplementary Table 13. KU4 results from Bayesian analysis.

Name	Unmodelled (BP)						Modelled (BP)	Amodel 70.1; Aoverall 69.7					Indices		
	mu	sigma	from 68.3	to 68.3	from 95.4	to 95.4	mu	sigma	from 68.3	to 68.3	from 95.4	to 95.4	A	C	C
Boundary							1660	850	2580	910	3345	-85			98.2
C_Date Woodland point	1800	700	2505	1095	3205	400	1660	850	2580	910	3345	-85	100.1		98.2
C_Date Archaic stemmed point	6750	200	6955	6545	7155	6350	6545	485	7070	6335	7345	5430	100.5		98.4
C_Date UW3691	11935	1290	13225	10640	14515	9350	11460	705	12355	10955	12680	10065	121.9		98.3
C_Date UW3692	9595	1010	10605	8580	11615	7570	9590	1005	10630	8570	11605	7590		5.7	98
C_Date Clovis knife	13050	200	13255	12845	13455	12650	12250	485	12800	12045	12940	11185	84		99.3
C_Date Pt Anomaly	12785	50	12840	12730	12890	12685	12840	200	13050	12715	13225	12410	98.5		99.3
C_Date UW3693	20435	1880	22315	18550	24195	16670	15625	2100	16960	12840	19835	12570	37.9		95.8
Boundary							15625	2100	16960	12840	19835	12570			95.8
P_Sequence Wakulla, KU4															
U(0,3)	1.52	0.87	0.00	3.00	0.00	3.00	2.46	0.68	2.49	3.00	0.00	3.00	100		99.4
Exp(1,-10,0.5)	-0.55	1.00	-0.66	0.45	-2.69	0.45	-0.75	1.27							99.9
Outlier_Model General							-400	835	-475	310	-2035	440			99.7

Supplementary Table 14. PU1 results from Bayesian analysis.

Name	Unmodelled (BP)						Modelled (BP)		Amodel 89.1; Aoverall 88.6				Indices	
	mu	sigma	from 68.3	to 68.3	from 95.4	to 95.4	mu	sigma	from 68.3	to 68.3	from 95.4	to 95.4	A	C
Boundary							9975	1145	11150	8785	12615	7815		98.5
C_Date UW3689	10235	1200	11435	9030	12635	7830	9975	1145	11150	8785	12615	7815	102.6	98.5
C_Date Pt Anomaly	12785	50	12840	12730	12890	12685	12760	135	12860	12700	13030	12410	100	99.8
C_Date UW3690	20535	2740	23275	17790	26015	15050	17805	2665	20075	14510	22660	...	76.5	96.4
Boundary							17805	2665	20075	14510	22660	...		96.4
P_Sequence Wakulla, PU1														
U(0,3)	1.52	0.87	0.00	3.00	0.00	3.00	1.47	0.86	0.00	2.30	0.00	2.84	100	99.8
Exp(1,-10,0.5)	-0.55	1.00	-0.66	0.45	-2.69	0.45	-0.54	0.99						100
Outlier_Model General							-65	265	-70	40	-580	280		100

Supplementary Table 15. PU7 results from Bayesian analysis.

Name	Unmodelled (BP)						Modelled (BP)	Amodel 110.1; Aoverall 110.4					Indices		
	mu	sigma	from 68.3	to 68.3	from 95.4	to 95.4	mu	sigma	from 68.3	to 68.3	from 95.4	to 95.4	A	P	C
Boundary							10925	1095	12360	10125	12765	8805		97.6	
C_Date UW3789	11235	1430	12665	9800	14095	8370	10925	1095	12360	10125	12765	8805	112.9	97.6	
C_Date Pt Anomaly	12785	50	12840	12730	12890	12685	12730	175	12860	12695	13010	12290	100	99.6	
C_Date UW3790	12435	1200	13635	11230	14835	10030	13025	455	13195	12660	14235	12235	118.9	98.6	
C_Date UW3788	22335	2370	24705	19960	27075	17590	21315	2625	24185	18770	26475	16025	92.9	95.5	
Boundary							21315	2625	24185	18770	26475	16025		95.5	
P_Sequence Wakulla, PU7															
U(0,3)	1.52	0.87	0.00	3.00	0.00	3.00	1.46	0.85	0.00	2.30	0.00	2.81	100	98.8	
Exp(1,-10,0.5)	-0.55	1.00	-0.66	0.45	-2.69	0.45	-0.54	0.98						99.9	
Outlier_Model General							-65	240	-65	40	-570	260		100	

Supplementary Table 16. PU15 results from Bayesian analysis.

Name	Unmodelled (BP)						Modelled (BP)	Amodel 125.9; Aoverall 127.6					Indices	
	mu	sigma	from 68.3	to 68.3	from 95.4	to 95.4	mu	sigma	from 68.3	to 68.3	from 95.4	to 95.4	A	C
Boundary							11120	475	11620	10665	12080	10160		97.7
C_Date Bolen point-2	10950	450	11405	10495	11855	10050	11120	475	11620	10665	12080	10160	94.8	97.7
C_Date Paleoindian scraper	12375	875	13255	11495	14130	10625	11825	465	12375	11410	12700	10930	111.2	99.4
C_Date Pt Anomaly	12785	50	12840	12730	12890	12685	12715	195	12860	12695	12975	12155	100	99
C_Date UW3787	12735	1850	14585	10880	16435	9030	12820	300	12965	12665	13510	12045	138.9	99.2
C_Date UW3786	12835	1350	14185	11480	15535	10130	13295	600	13585	12725	14720	12235	124	95.1
Boundary							13295	600	13585	12725	14720	12235		95.1
P_Sequence Wakulla, PU15														
U(0,3)	1.52	0.87	0.00	3.00	0.00	3.00	1.45	0.84	0.00	2.48	0.00	2.84	100	97.7
Exp(1,-10,0.5)	-0.55	1.00	-0.66	0.45	-2.69	0.45	-0.54	0.97						99.9
Outlier_Model General							-60	200	-65	35	-565	230		100

Supplementary Table 17. PU23 results from Bayesian analysis.

Name	Unmodelled (BP)						Modelled (BP)			Amodel 91.5; Aoverall 90.2			Indices	
	mu	sigma	from 68.3	to 68.3	from 95.4	to 95.4	mu	sigma	from 68.3	to 68.3	from 95.4	to 95.4	A	C
Boundary							9470	975	10665	8735	11220	7465		99
C_Date UW3813	9025	1190	10215	7830	11405	6640	9470	975	10665	8735	11220	7465	101.9	99
C_Date Bolen point	10950	450	11405	10495	11855	10050	10875	455	11335	10440	11785	9970	102.3	99.7
C_Date C_Date Pt Anomaly	12785	50	12840	12730	12890	12685	12795	85	12870	12735	12960	12620	97.3	99.3
C_Date Paleo scraper-graver	13095	195	13295	12895	13490	12705	12895	140	12980	12740	13230	12670	87.7	97.8
C_Date Paleo turtle-back scraper	13095	195	13295	12895	13490	12705	12925	150	13020	12755	13270	12685	93.1	98
C_Date UW3814	12535	1550	14085	10980	15635	9430	13865	730	14195	12940	15380	12790	95.5	99.3
C_Date UW3815	25635	3440	29075	22190	32515	18750	23955	3415	27395	20470	30630	17130	94.5	96.8
Boundary							23955	3415	27395	20470	30630	17130		96.8
P_Sequence Wakulla, PU23														
U(0,3)	1.52	0.87	0.00	3.00	0.00	3.00	1.36	0.78	0.08	2.31	0.00	2.62	100	99.8
Exp(1,-10,0.5)	-0.55	1.00	-0.66	0.45	-2.69	0.45	-0.53	0.97						100
Outlier_Model General							-40	140	-50	30	-355	150		100

KU4 Bayesian coding

```
Options()
 {
 Resolution=5;
 Curve="IntCal20";
 BCAD=FALSE;
 kIterations=100;
 };
 Plot()
 {
Outlier_Model("General",Exp(1,-10,0.5),U(0,3),"t");
P_Sequence("Wakulla, KU4",0.2,2)
 {
Boundary();

C_Date("UW3693",calBP(2018-20500),1880){z=111.0;Outlier("General",1);};
C_Date("Pt Anomaly",calBP(1950-12785),50){z=106.8;Outlier("General",1);};
C_Date("Clovis knife",calBP(1950-13050),200){z=96.0;Outlier("General",1);color="orange";};
C_Date("UW3692",calBP(2018-9660),1010){z=96.0;Outlier();color="red";};
C_Date("UW3691",calBP(2018-12000),1290){z=90.0;Outlier("General",1);};
```

```
C_Date("Archaic stemmed point",calBP(1950-
6750),200){z=59.0;Outlier("General",1);color="orange";};
C_Date("Woodland point",calBP(1950-1800),700){z=28.0;Outlier("General",1);color="orange";};

Boundary();
 };
 };
```

PU1 Bayesian coding

```
Options()
 {
 Resolution=5;
 Curve="IntCal20";
 BCAD=FALSE;
 kIterations=100;
 };
 Plot()
 {
Outlier_Model("General",Exp(1,-10,0.5),U(0,3),"t");
P_Sequence("Wakulla, PU1",0.3,2)
 {
Boundary();

C_Date("UW3690",calBP(2018-20600),2740){z=130.0;Outlier("General",1);};
C_Date("Pt Anomaly",calBP(1950-12785),50){z=108.8;Outlier("General",1);};
C_Date("UW3689",calBP(2018-10300),1200){z=90.0;Outlier("General",1);};

Boundary();
 };
 };
```

PU7 Bayesian coding

```
Options()
 {
 Resolution=5;
 Curve="IntCal20";
 BCAD=FALSE;
 kIterations=100;
 };
 Plot()
 {
Outlier_Model("General",Exp(1,-10,0.5),U(0,3),"t");
P_Sequence("Wakulla, PU7",0.1,2)
 {
```

```
Boundary();

C_Date("UW3788",calBP(2018-22400),2370){z=133.0;Outlier("General",1);};
C_Date("UW3790",calBP(2018-12500),1200){z=107.0;Outlier("General",1);};
C_Date("Pt Anomaly",calBP(1950-12785),50){z=103.8;Outlier("General",1);};
C_Date("UW3789",calBP(2018-11300),1430){z=90.0;Outlier("General",1);};

Boundary();
 };
 };
```

PU15 Bayesian coding

```
Options()
 {
 Resolution=5;
 Curve="IntCal20";
 BCAD=FALSE;
 kIterations=100;
 };
 Plot()
 {
Outlier_Model("General",Exp(1,-10,0.5),U(0,3),"t");
P_Sequence("Wakulla, PU15",0.05,2)
 {
Boundary();

C_Date("UW3786",calBP(2018-12900),1350){z=120.0;Outlier("General",1);};
C_Date("UW3787",calBP(2018-12800),1850){z=103.0;Outlier("General",1);};
C_Date("Pt Anomaly",calBP(1950-12785),50){z=98.8;Outlier("General",1);};
C_Date("Paleoindian scraper",calBP(1950-12375),875){z=66.0;Outlier("General",1);color="orange";};
C_Date("Bolen point-2",calBP(1950-10950),450){z=48.0;Outlier("General",1);color="orange";};

Boundary();
 };
 };
```

PU23 Bayesian coding

```
Options()
 {
 Resolution=5;
 Curve="IntCal20";
 BCAD=FALSE;
 kIterations=100;
 };
 Plot()
```

```
 {
Outlier_Model("General",Exp(1,-10,0.5),U(0,3),"t");
P_Sequence("Wakulla, PU23",0.3,2)
 {
Boundary();

C_Date("UW3815",calBP(2018-25700),3440){z=120.0;Outlier("General",1);};
C_Date("UW3814",calBP(2018-12600),1550){z=103.0;Outlier("General",1);};
C_Date("Paleo turtle-back scraper",calBP(1950-13095),195){z=99;
Outlier("General",1);color="orange";};
C_Date("Paleo scraper-graver",calBP(1950-13095),195){z=99; Outlier("General",1);color="orange";};
C_Date("Pt Anomaly",calBP(1950-12785),50){z=98.8; Outlier("General",1);};
C_Date("Bolen point",calBP(1950-10950),450){z=93.0; Outlier("General",1);color="orange";};
C_Date("UW3813",calBP(2018-9090),1190){z=90.0;Outlier("General",1);};

Boundary();
 };
 };
```

References

1. Allen, W. J. *Soil Survey of Wakulla County, Florida.* United States Department of Agriculture, Washington, DC (1991).

2. Ahlbrandt, T. S., and Fryberger, S. G., Eolian Deposits, *in* Scholle P. A., and Spearing D., eds., Sandstone Depositional Environments: Tulsa, Oklahoma, The American Association of Petroleum Geologists, p.11-47 (1982).

3. Davis, R. A., Jr., Depositional Systems: A Genetic Approach to Sedimentary Geology: Englewood Cliffs, New Jersey, Prentice-Hall, Inc., 669 p. (1983)

4. Folk, R.L. *Petrology of Sedimentary Rocks*. Hemphill Publ., Austin TX (1974).

5. Moore, C. R. *et al*. Widespread platinum anomaly documented at the Younger Dryas onset in North American sedimentary sequences. *Scientific Reports* **7,** (44031), https://doi.org/10.1038/srep44031 (2017).

6. Moore, C. R. *et al*. Regional Manifestations of Late Quaternary Climate Change and Archaeological Site Burial along the South Atlantic Coastal Plain. In *Early Human Life on the Southeastern Coastal Plain*, edited by Albert C. Goodyear and Christopher R. Moore, pp. 234-259. University of Florida Press, Gainesville (2018).

7. Dunbar, J. S. *et al.* The Wakulla Springs Lodge Site (8WA329) Final Report of Investigations. Submitted to Florida Master Site File manuscript 25374 (2018).

8. Dunbar, J. S. *et al.* The Wakulla Springs Mysterious Waters: a report of archaeological investigations. DHR Grants Office by the Aucilla Research Institute for DHR Grant 18.h.sc.300.041. Florida Master Site File manuscript 26243, Tallahassee, Florida (2019).

9. Feathers, James K. Use of Luminescence Dating in Archaeology. *Measurement and Science Technology* **14**:1493–1509 (2003).

10. Murray, A. S., and A. G. Wintle Luminescence Dating of Quartz Using an Improved Single-Aliquot Regenerative-Dose Protocol. *Radiation Measurements* **32**:57-73 (2000).

11. Wintle, A. G., & Murray, A. S., A review of Quartz Optically Stimulated Luminescence Characteristics and Their Relevance in Single-Aliquot Regeneration Dating Protocols. *Radiation Measurements* **41**:369-391 (2006).

12. Galbraith, R.F. and Roberts, R.G. Statistical aspects of equivalent dose and error calculation and display in OSL dating: An overview and some recommendations, *Quaternary Geochronology* **11** (2012), https://doi.org/10.1016/j.quageo.2012.04.020.

13. Kennett, J. P. *et al.* Bayesian chronological analyses consistent with synchronous age of 12,835-12,735 Cal B.P. for Younger Dryas boundary on four continents. *Proc Nat Acad Sci* **112**, E4344-4353 (2015).

14. Wolbach, W. S. *et al.* Extraordinary biomass-burning episode and impact winter triggered by the Younger Dryas cosmic impact <12,800 years ago. 1. Ice cores and glaciers. *J Geol* **126**, 165–184 (2018a).
